# The causal associations of 25(OH)D and its metabolites with oropharyngeal cancer risk: a Mendelian randomization study

**DOI:** 10.2340/aos.v84.44053

**Published:** 2025-07-17

**Authors:** YaoHui Yu, Yu Zhou

**Affiliations:** a Department of Children Stomatology and Oral Prevention, School and Hospital of Stomatology, Wenzhou Medical University, Wenzhou, Zhejiang, P.R. China; b Department of Orthodontics, School and Hospital of Stomatology, Wenzhou Medical University, Wenzhou, Zhejiang, P.R. China

**Keywords:** oropharyngeal cancer, mendelian randomization, 25(OH)D, 25(OH)D3, C3-epi-25(OH)D3

## Abstract

**Background:**

Previous studies have suggested that there are distinct correlations of 25-hydroxyvitamin D (25(OH)D) and its metabolites with the risk of developing health conditions and cancer; however, the precise nature of these associations in patients with oropharyngeal cancer (OPC) is unknown. Our primary objective was to evaluate the causal impact of 25(OH)D and its metabolites, including 25(OH)D3 and its epimer C3-epi-25(OH)D3, on susceptibility to OPC through the use of Mendelian randomization (MR) methodology.

**Methods:**

Mendelian randomization analysis was performed on data from 291 patients with OPC from Europe, North America, and South America using genetic variant strongly related to C3-epi-25(OH)D3, 25(OH)D, and 25(OH)D3 exposure. The primary analytical method for two-sample MR analysis was inverse-variance weighting (IVW); supplemental analyses (weighted median [WM], MR–Egger) were also conducted. Leave-one-out and Cochran’s Q tests were concurrently used as sensitivity analyses to test and adjust for pleiotropy.

**Results:**

Our MR analysis provided evidence suggesting that greater 25(OH)D3 levels are causally associated with a decreased risk of developing OPC within the European population (WM OR = 0.47, 95% CI = 0.24–0.91, *p* = 0.03). Only one of the 21 MR analyses yielded significant results; for this MR analysis, the IVW results were significant, but subsequent leave-one-out analyses revealed instability in the causal association. However, the association was significant when rs9304669 was excluded (OR = 0.51, 95% CI = 0.28–0.91, *p* = 0.02), whereas the other results were not statistically significant. The sensitivity analysis indicated that the results were reliable, with no observed heterogeneity or pleiotropy.

**Conclusions:**

There was no evidence that 25(OH)D, 25(OH)D3 or C3-epi-25(OH)D3 levels are associated with OPC risk or that 25OHD supplementation in the general population prevents OPC.

The registration number is INPLASY202490081.

## Introduction

Recently, a growing body of research has increased interest in the intricate interplay between the levels of 25-hydroxyvitamin D (25(OH)D) and its metabolites, including both 25(OH)D3 and its epimer C3-epi-25(OH)D3, in various cancers and chronic diseases [1–4]. Observational research has revealed a connection between increased cancer risk and reduced serum 25(OH)D levels; moreover, vitamin D receptors are expressed in diverse tissues, suggesting that vitamin D pathways may influence carcinogenesis [1, 5–7].

However, hypercalcemia severely limits the clinical application of vitamin D [8]. The safety assessment of vitamin D supplementation is particularly important because patients with head and neck squamous cell carcinoma (HNSCC) often have abnormal renal function or bone metastases, which increases the risk of hypercalcemia. Therefore, it remains unknown whether there are causal associations (and, if so, their direction) between cancer and lifelong exposure to 25(OH)D, C3-epi-25(OH)D3, and 25(OH)D3 [1, 7]; in the context of this study, these three variables are referred to as the ‘Vit D set’.

There has been a notable focus on HNSCC, which includes oral cavity cancer and oropharyngeal cancer (OPC), in the context of vitamin D research. Head and neck squamous cell carcinoma represents a significant global health concern, with a substantial number of cases and fatalities annually [9, 10]. The lack of a correlation between lifelong exposure to 25(OH)D3 and its metabolites and OPC risk in recent analytical studies adds complexity to the understanding of the relationship between vitamin D and cancer. The absence of a correlation in specific types of cancer emphasizes the need for nuanced investigations, as different cancers may have distinct etiological factors and pathways.

Mendelian randomization (MR) is used in epidemiology and genetic research to investigate causal relationships between modifiable exposures (such as vitamin D levels) and outcomes (such as cancer) while minimizing biases from measurement errors, confounding factors, and reverse causality. Because genetic variants are randomly assigned at conception and are not affected by disease states, MR can be used to avoid reverse causality, which is common in observational studies [11, 12].

In the context of vitamin D and cancer, MR studies using genetic variants associated with vitamin D levels can provide valuable insights into the potential causal relationship between vitamin D and cancer risk. These studies can help overcome some of the limitations of observational research and contribute to a more robust understanding of the role of vitamin D in cancer prevention. The objective of this study was to employ MR to investigate the underlying mechanism of and causal connection of 25(OH)D3 and its metabolites with OPC risk in 96 Europeans, 97 North Americans and 98 South Americans [2–4]. To date, there have been no reports of studies addressing these problems via MR analysis.

## Methods

The current MR study was conducted according to the Preferred Reporting Items for Systematic Reviews and Meta-analyses (PRISMA) statement. The initial screening was independently completed by two authors. The study was designed to carefully examine the causal relationships of the levels of 25(OH)D3 and its metabolites with and OPC risk. We screened PubMed for meta-analyses or systematic reviews dated from inception to February 2024 that investigated correlations between exposure to 25(OH)D, C3-epi-25(OH)D3, and 25(OH)D3 and OPC in European, North American, and South American populations. In addition, we manually searched related references in the identified literature for other potentially relevant articles.

### Inclusion and exclusion criteria

The inclusion criteria for the studies were as follows: (1) a genome-wide association study (GWAS) based on the large UK Biobank (UKB) dataset and the Carolina Head and Neck Cancer Study [2–4]; (2) exposure to 25(OH)D, C3-epi-25(OH)D3, and 25(OH)D3; (3) OPC; and (4) independent single-nucleotide polymorphisms (SNPs) (*p* < 5 × 10^−8^).

The exclusion criteria of the studies were as follows: (1) SNPs with linkage disequilibrium *r*^2^ < 0.001 and a clumping distance of 10,000 kb; (2) an F-statistic threshold of F < 10; and (3) SNPs strongly correlated with the outcome.

### Data extraction method

Two authors independently extracted the data according to the inclusion and exclusion criteria.

### Sensitivity analyses

If genetic instruments are ineffective or independent solely in the absence of horizontal pleiotropy or in the presence of equilibrium, the IVW estimation method may be susceptible to bias [13].

Therefore, we performed additional analyses via the weighted median (WM) and MR–Egger methods to identify imbalanced horizontal pleiotropy; these methods are described in detail elsewhere [14]. In short, the WM specifies that at least 50% of the weights in the analysis come from effective tools [15].

Even if the mutation is invalid, the MR–Egger approach provides credible effect calculations, which can be adjusted and used to test for directional pleiotropy [16]. To further evaluate the stability of the results, we (1) scrutinized the potential for ineffective instrumental variables, particularly in cases of horizontal pleiotropy, to assess heterogeneity among individual genetic variables via Cochran’s Q statistic; (2) utilized the Egger intercept test to detect pleiotropy; and (3) generated scatter plots and performed leave-one-out tests to investigate whether the IVW method was influenced by specific SNPs.

**Figure 1:**
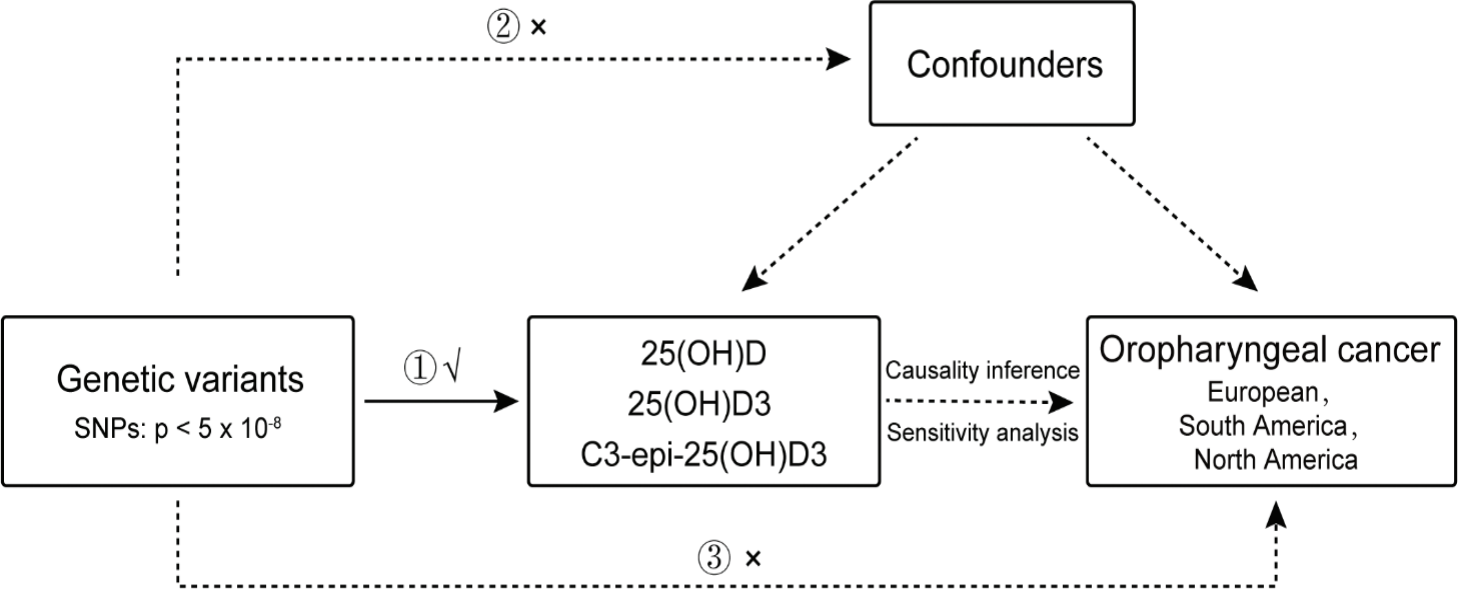
Overview of the design of the present study. a Design of the Mendelian randomization (MR) analysis of putative causal associations of 25-hydroxyvitamin D (25(OH)D) metabolites, 25 hydroxyvitamin D concentration and the epimeric form (C3-epi-25(OH)D3) with risk of the oral cavity and oropharynx (OPC), 96 participants in European, 97 participants in North America and 98 participants in South America. Genetic variants can act as proxies or instruments to investigate if an exposure (25(OH)D , 25(OH)D3 and C3-epi-25(OH)D3) is associated with a disease outcome (the oral cavity and oropharynx [OPC]). Causal inference can be made between exposure and outcome if the following conditions are upheld. (1) The genetic variants which make up the instrument are valid and reliably associated with the exposure (i.e. the ‘relevance assumption’); (2) There is no measured or unmeasured confounding of the association between the genetic instrument and the outcome (i.e. the ‘exchangeability’ assumption); (3) There is no independent pathway between the genetic instrument and the outcome, except through the exposure (i.e. the ‘exclusion restriction principle’).

To further support our analysis, we performed sensitivity analyses with the MR–Egger and WM approaches. These methodologies are instrumental for detecting horizontal or uncorrelated pleiotropy, in which genetic variants impact both the exposure (Vit D set) and the outcome (OPC) through distinct mechanisms. In contrast, correlated pleiotropy can evoke spurious associations in MR. In such cases, genetic variants influence both the exposure and the outcome via shared heritable factors. It is imperative to consider the possibility of correlated pleiotropy within the instruments related to 25(OH)D3 and its metabolites, as its detection is crucial to prevent false-positive results (Figure 1).

All analyses were conducted within the R programming environment (version 4.1.3) with the TwoSampleMR package (version 0.5.6).

## Results

Following the removal of SNPs above the significance threshold of *p* < 5 × 10^−8^ and those exhibiting low linkage disequilibrium (LDr2 < 0.001), a clumping distance of 10,000 kb, and a low F statistic (F < 10) (Supplementary Data 1 and 2), a total of 291 independent SNPs (*p* < 5 × 10^−8^) displayed independent and robust correlations with the exposure variable, namely, 25(OH)D3 and its metabolites. Among these, 96 SNPs originated from the European population, 97 from the North American population, and 98 from the South American population [2–4]. To elucidate the potential risk factors for OPC, a dual-sample MR analysis was conducted. This approach involves the selection of relevant SNPs to identify risk factors associated with OPC [2–4]. We subsequently calculated SNP-specific Wald estimates (obtained by dividing the SNP result estimate by the SNP exposure estimate) and applied the IVW method to conduct a meta-analysis of these estimates. This allowed us to derive an estimate of the impact of these risk factors on OPC risk.

**Figure 2:**
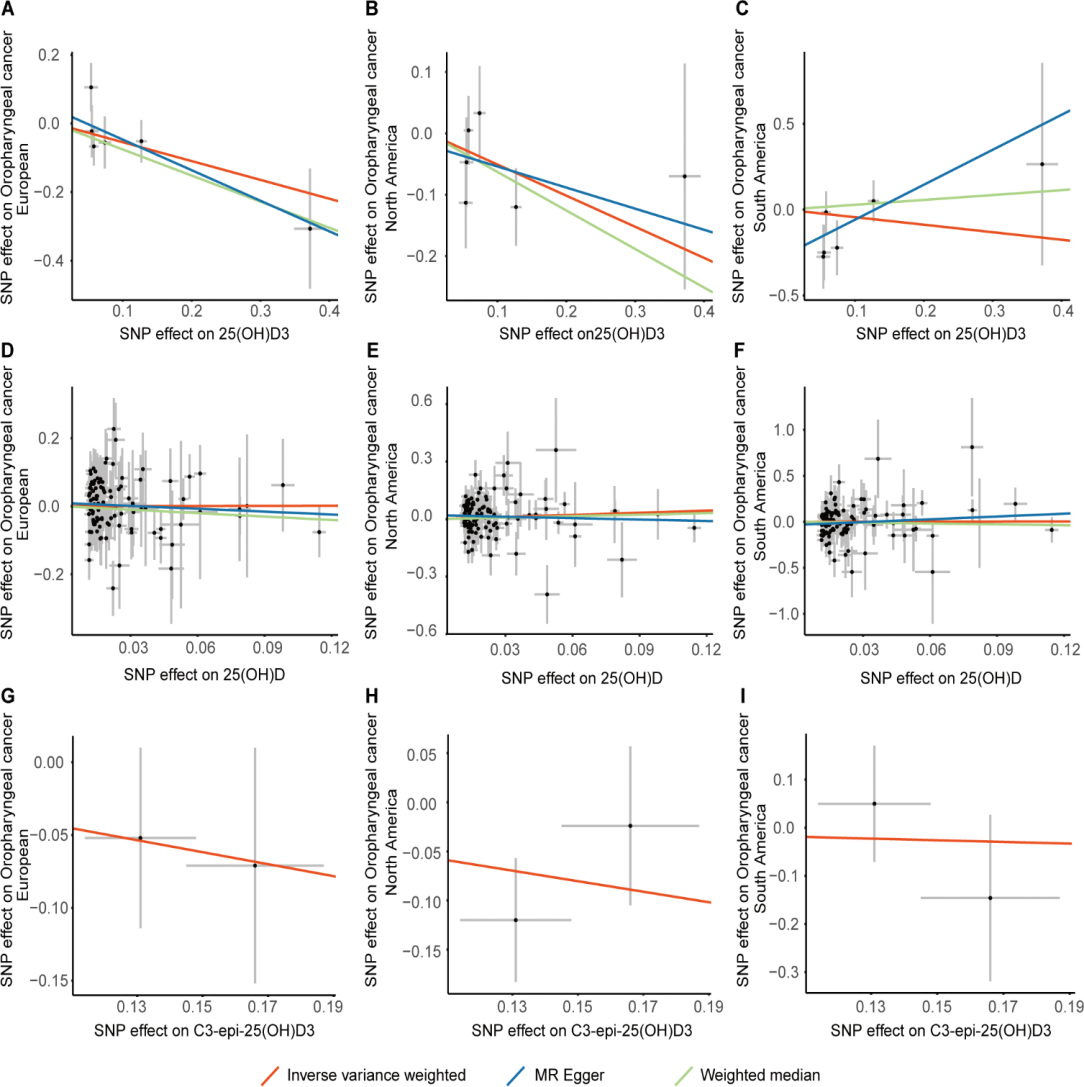
Mendelian randomization results of 25(OH)D , 25(OH)D3 and C3-epi-25(OH)D3 with risk of OPC. A, B, C showed that SNP effect on 2(OH)D3 from European North America and South America. D, E, F showed that SNP effect on 25(OH)D from European North America and South America. G, H, I showed that SNP effect on C3-epi-25(OH)D3 from European North America and South America..

Our analysis, which used the IVW, WM, and MR–Egger methods, produced compelling evidence indicating that (25(OH)D, 25(OH)D3 and C3-epi-25(OH)D3 have no discernible effect on the risk of developing OPC among European, North American and South American populations (Table 1 and Figure 2). Intriguingly, within the European population, we observed that a greater 25(OH)D3 level is causally associated with a decreased risk of developing OPC, with a WM odds ratio (OR) of 0.47 (95% CI = 0.24–0.91, *p* = 0.03). Only one of the 21 MR analyses produced significant results (Table 1). The IVW results were significant, but subsequent leave-one-out analyses revealed instability in the negative causal association.

**Table 1:** MR analysis.

Outcomes	Exposure	Methods	nSNP	OR (95% CI)	*p*
Oropharyngeal cancer (European)	25(OH)D	IVW	109	1.01 (0.61, 1.67)	0.96
		WM	109	0.71 (0.31, 1.66)	0.43
		MR Egger	109	0.76 (0.33, 1.73)	0.51
	25(OH)D3	IVW	6	0.58 (0.33, 1.02)	0.06
		WM	6	0.47 (0.24, 0.91)	**0.03**
		MR Egger	6	0.41 (0.15, 1.13)	0.16
	C3-epi-25(OH)D3	IVW	2	0.66 (0.34, 1.29)	0.23
Oropharyngeal cancer (North America)	25(OH)D	IVW	110	1.44 (0.84, 2.49)	0.19
		WM	110	1.29 (0.61, 2.74)	0.5
		MR Egger	110	0.78 (0.32, 1.92)	0.59
	25(OH)D3	IVW	6	0.60 (0.34, 1.08)	0.09
		WM	6	0.53 (0.26, 1.11)	0.09
		MR Egger	6	0.71 (0.25, 2.03)	0.56
	C3-epi-25(OH)D3	IVW	2	0.59 (0.27, 1.25)	0.16
Oropharyngeal cancer (South Ame rica)	25(OH)D	IVW	109	1.04 (0.37, 2.88)	0.95
		WM	109	0.74 (0.15, 3.62)	0.71
		MR Egger	109	2.76 (0.53, 14.30)	0.23
	25(OH)D3	IVW	6	0.65 (0.14, 2.96)	0.57
		WM	6	1.33 (0.28, 6.37)	0.72
		MR Egger	6	7.67 (0.44, 133.20)	0.23
	C3-epi-25(OH)D3	IVW	2	0.84 (0.22, 3.26)	0.8

IVW: inverse variance weighted; WM: weighted median; 25(OH)D: 25-hydroxyvitamin D; SNP: single-nucleotide polymorphisms.

### Sensitivity analyses

**Figure 3:**
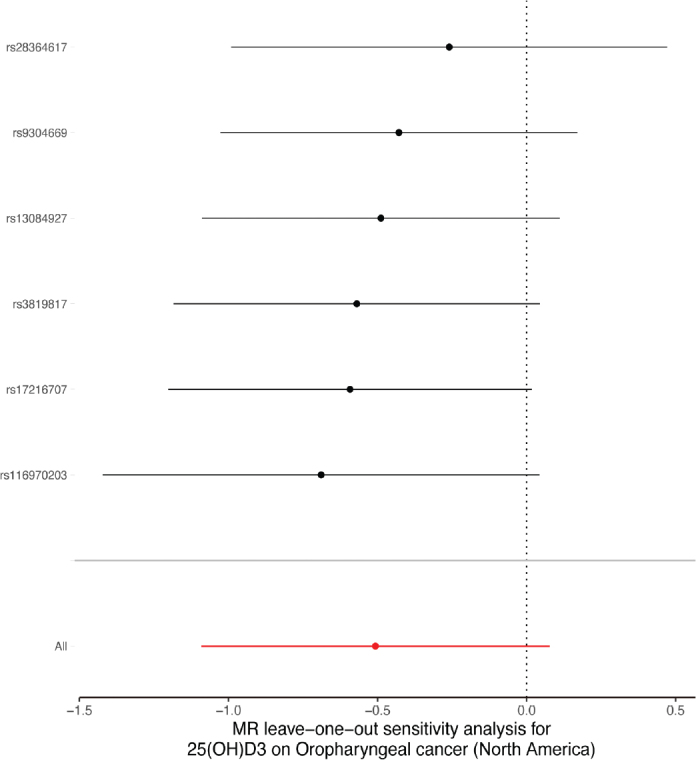
Scatter and leave-one-out plots demonstrating influential outliers in univariable MR of 25(OH)D3 and oral and oropharyngeal cancer risk (North America).

**Figure 4:**
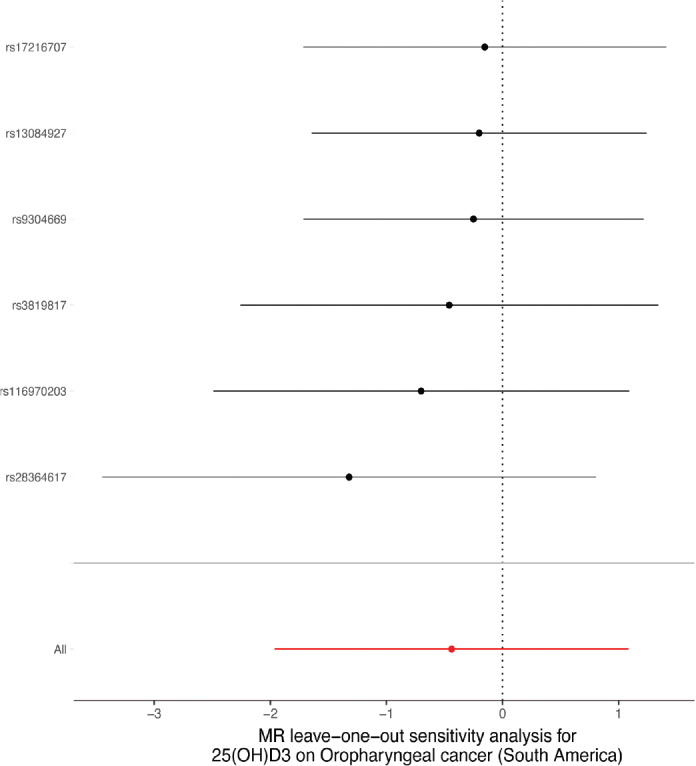
Scatter and leave-one-out plots demonstrating influential outliers in univariable MR of 25(OH)D3 and oral and oropharyngeal cancer risk (South America).

**Figure 5:**
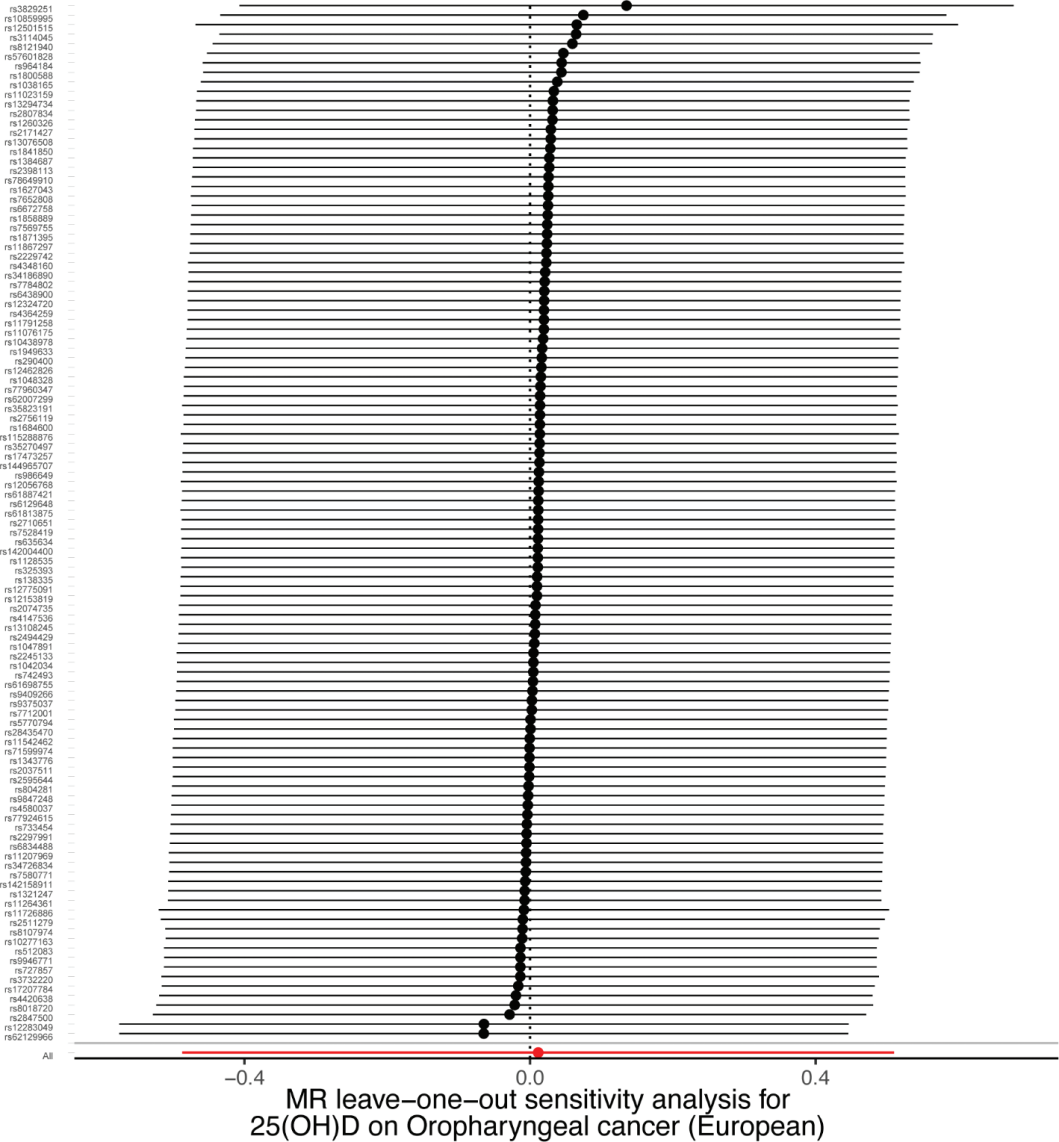
Scatter and leave-one-out plots demonstrating influential outliers in univariable MR of 25(OH)D and oral and oropharyngeal cancer risk (European).

**Figure 6:**
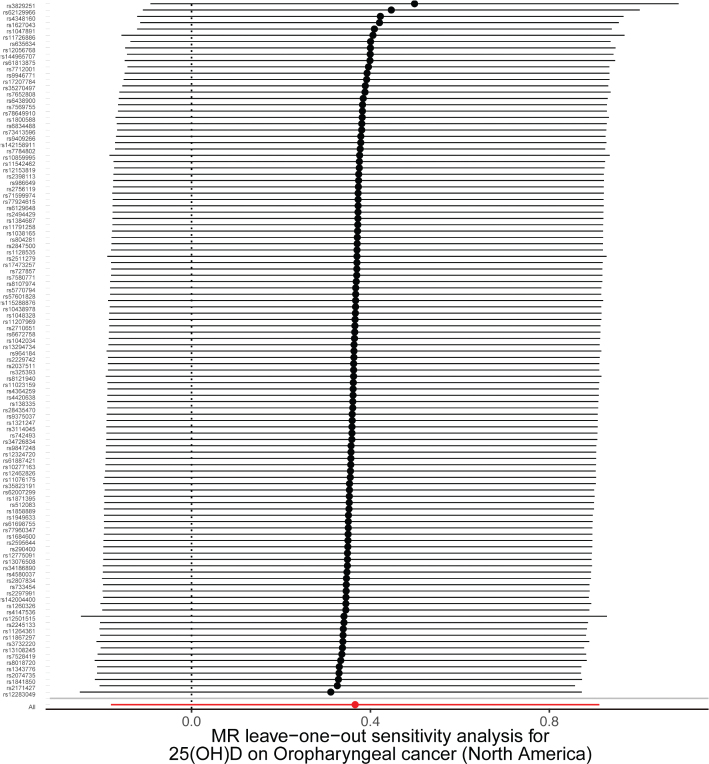
Scatter and leave-one-out plots demonstrating influential outliers in univariable MR of 25(OH)D and oral and oropharyngeal cancer risk (North America).

**Figure 7:**
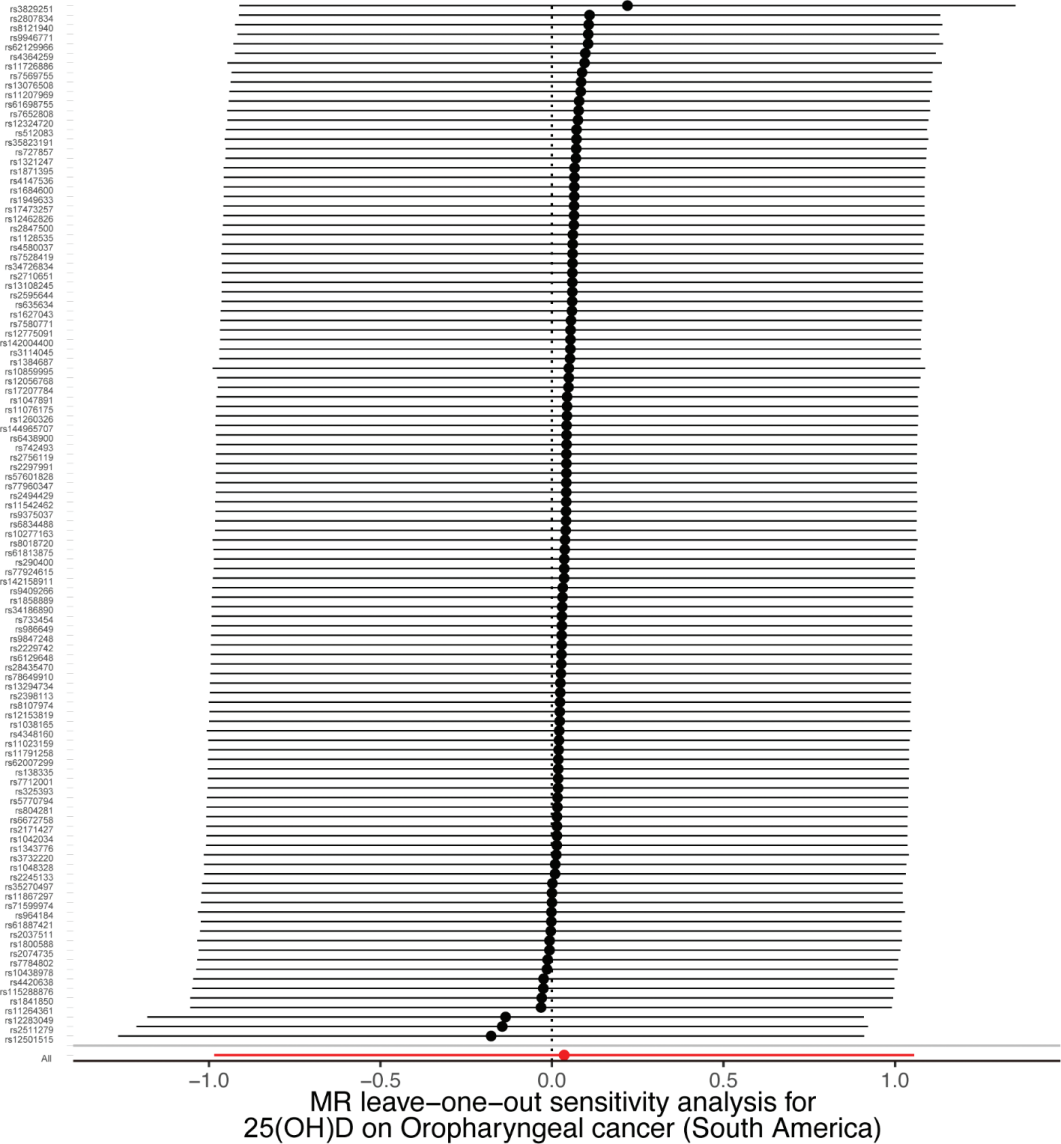
Scatter and leave-one-out plots demonstrating influential outliers in univariable MR of 25(OH)D and oral and oropharyngeal cancer risk (South America).

**Figure 8:**
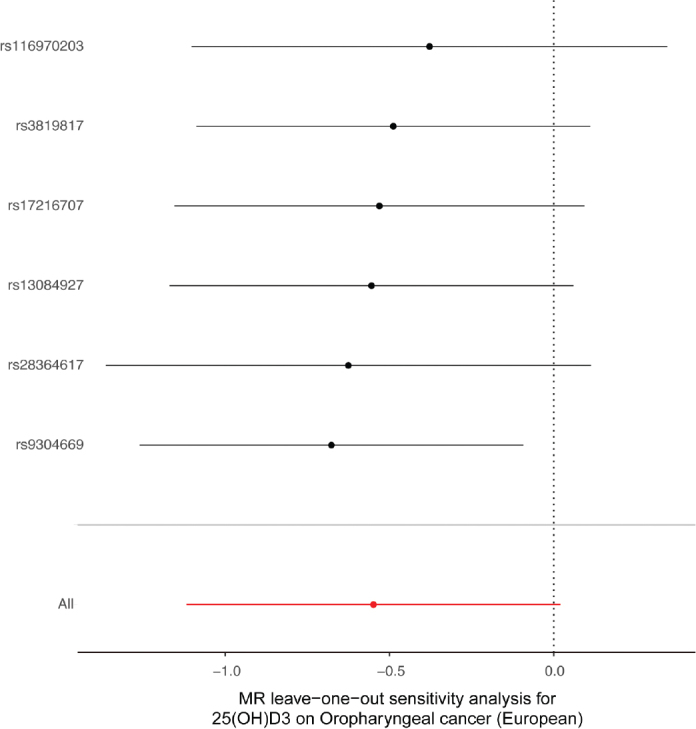
Scatter and leave-one-out plots demonstrating influential outliers in univariable MR of 25(OH)D3 and oral and oropharyngeal cancer risk (European). The results became positive when this variant (rs9304669) was excluded from the analysis.

A sensitivity analysis involving various MR methods revealed that the primary MR results were reliable, with no evidence of heterogeneity or pleiotropy (Figures 2–7, Table 2). In contrast, the scatter plot and leave-one-out test revealed instability in the negative result obtained through the primary IVW method (Figure 8). However, after an outlier (the genetic variant rs9304669) was excluded, the outcomes shifted in a positive direction (OR = 0.51, 95% CI = 0.28–0.91, *p* = 0.02). Notably, the remaining results continued to indicate a negative association between 25(OH)D3 levels and OPC risk (Table 2).

**Table 2:** Sensitivity analysis.

Outcome	Exposure	Heterogeneity		Pleiotropy	
		Cochran’s Q	*p*	Egger intercept	*p*
Oropharyngeal cancer (European)	25(OH)D	102.02	0.64	0.01	0.39
	25(OH)D3	4.47	0.48	0.04	0.47
	C3-epi-25(OH)D3	0.002	0.96	NA	NA
Oropharyngeal cancer (North America)	25(OH)D	133.5	0.06	0.02	0.1
	25(OH)D3	3.82	0.58	–0.02	0.73
	C3-epi-25(OH)D3	1.27	0.26	NA	NA
Oropharyngeal cancer (South America)	25(OH)D	94.26	0.82	–0.03	0.14
	25(OH)D3	6.46	0.26	–0.26	0.12
	C3-epi-25(OH)D3	0.82	0.37	NA	NA

25(OH)D: 25-hydroxyvitamin D.

## Discussion

This study is the first MR investigation aimed at discerning the potential bidirectional associations of the levels of 25(OH)D3 and its metabolites with OPC risk. However, the findings indicate an absence of any discernible impact of 25(OH)D3 and its metabolites on OPC risk. This finding is in agreement with a previous investigation [17], whose results suggest that the causal relationship between 25(OH)D levels and the risk of cancer morbidity or mortality is not supported. This conclusion is attributed to the limited statistical power of current OPC GWASs, which are not sufficient for comprehensively elucidating the extent to which 25(OH)D3 and its metabolites contributes to OPC risk. Although modest effects were detected, specifically regarding the influence of 25(OH)D3 level on OPC risk within the European population, the effects remained statistically significant when subjected to sensitivity analyses. Accounting for the level of pleiotropy, the outcomes of the studies were largely reliable.

Previous studies [18–20, 22–24] have revealed certain correlations between the levels of vitamin D and its metabolites and several health outcomes. These associations can be explained by a single genetic variant with implications for various health conditions, including mental illnesses and cancer. In the context of oncology, vitamin D attenuates tumor invasiveness and metastasis, thereby reducing the cancer mortality rate [20].

Keum et al. [21] reported that elevated 25(OH)D levels in cancer patients during diagnosis or treatment are closely linked with prolonged survival. Observational studies [22, 23] have posited that vitamin D might be most protective against cancer-related mortality during the initial stages of clinically detectable cancer, with the most robust inverse correlations observed in the context of colorectal cancer. A previous study on site-specific cancers revealed a notable connection between 25(OH)D levels and melanoma risk. This highlights the need for a balanced and personalized approach to sun exposure and vitamin D supplementation, especially in high-risk populations [24]. However, the risk of cancer of the digestive system cannot be mitigated simply by increasing vitamin D intake, as there is no relationship between vitamin D levels and digestive system cancers [25].

Earlier studies have produced conflicting results, and there is considerable controversy about the impact of 25(OH)D concentrations on cancer risk or mortality. Ong et al. [26] revealed a lack of genetic evidence for an association between vitamin D concentrations and the overall prognosis of cancer. However, another study showed [27] that an increase in the plasma vitamin D concentration may reduce the risk of ovarian cancer in Europeans. Different results have been reported for studies in colorectal cancer. Katagiri et al. [28] reported that no notable linkage was observed between 25(OH)D levels and colorectal cancer among individuals of European ancestry and Asian populations. McCullough et al. [29] reported that higher circulating 25(OH)D concentrations (75–100 nmol/L) are associated with a reduced risk of developing colorectal cancer. However, Yejin Kim et al. [30] reported that low or deficient 25(OH)D may increase the risk of developing adenocarcinoma and colon cancer. Although the apparent effect of the 25(OH)D concentration decreases with time, the available data [31] support that vitamin D plays an important role in protecting against several types of cancer. Chandler PD et al. [32] reported that vitamin D3 supplementation reduces the incidence of advanced (metastatic or fatal) cancers, especially among participants with a BMI < 25 kg/m^2^, and decreases the overall incidence of cancer. Nevertheless, another investigation [33] revealed that vitamin D supplementation did not reduce the incidence of invasive cancer or cardiovascular events compared with a placebo.

Most randomized controlled trials (RCT) have been designed to investigate pharmaceutical drugs rather than nutrients, and studies using such an approach have shown the benefits of vitamin D in reducing the risk of cancer. However, vitamin D levels are usually too low [34, 35]. Low vitamin D levels may be associated with an increased risk of various cancers, such as those of the breast, colon and prostate [36]. These findings agree with those from a previous study [37] that highlighted vitamin D levels as a potential determinant of cancer immunity and the success of immunotherapy.

In the context of vitamin D and calcium supplementation, the findings of Lappe et al. [38] suggest that such interventions do not significantly lower the cancer risk among post-menopausal women over a span of 4 years. Nonetheless, a separate study conducted by Lappe and colleagues [39] showed that supplementation with vitamin D and calcium is associated with a reduction in cancer risk among older postmenopausal women.

One ecological report [40] revealed that solar UVB radiation is directly linked to oral cavity and pharynx cancer, indicating that adequate vitamin D levels do not prevent this process. However, smoking may have inffuenced the results of this previous analysis. Vitamin D supplementation is ineffective [8] because organs impacted by cancer can generate calcitriol from circulating 25(OH)D and do not need to use circulating calcitoriol.

A certain degree of correlation has been observed between human papilloma virus (HPV) and OPC. Nevertheless, a recent study [41] revealed that HPV status and vitamin D adequacy are not notably associated within a predominantly male cohort of OPC patients. The findings from this investigation indicate that vitamin D status has potential as a prognostic biomarker in patients with OPC. However, additional research is imperative to validate this association. Moreover, Avila et al. [42]. reported that maintaining optimal vitamin D levels may yield favorable effects within the initial stages of cervical cancer by potentially mitigating its onset and development.

However, our OPC study has several limitations. The fact that only one of the 21 MR analyses was significant indicates that the MR analysis results are weak. This could be due to the low number of OPC cases. The *p* value of 0.03 indicates that the findings were near the threshold for being considered random. In addition, considering the long incubation period of cancer development, prolonged follow-up is necessary to fully ascertain the potential impact of any factor on cancer risk. In addition, our study was unable to detect very small impacts, as included data from a very large genetic epidemiological network were included. Further research is necessary to assess the possible role of increasing the levels of 25(OH)D3 and its metabolites in preventing OPC.

In our study, although the independent effects of 25(OH)D3 and its metabolites indicate potential underestimation of the random double-blind estimates owing to methodological variation and the need to interpret estimates, these effects cannot be directly compared. Mendelian randomization analysis potentially reflects the impact of lifelong exposure to the Vit D set. Univariable MR enables the assessment of the autonomous influence of exposure on OPC risk, without any confounding factors. Nonetheless, it lacks the capacity to gauge the extent to which this influence is modified by confounding variables, such as exposure concentration or duration. A more granular analysis of individual-level data is necessary for estimating the effects of such confounding factors. This study may have other limitations, and our conclusions may not be applicable to other racial groups, mainly because the participants were from Europe and the Americas. Moreover, owing to the insufficient sample size, the current OPC sample was only sufficiently powered to detect an effect with an OR > 1.25 and may have missed a small effect of 25(OH)D3 and its metabolites.

Although there is still a lack of RCT data, some clinical, preclinical, epidemiological, and in vitro experimental data indicate that vitamin D signaling may be beneficial for preventing and treating various cancers [7, 19–24]. Giammanco et al. [43] reported that several interventions targeting vitamin D metabolism or activity disorders have been used for cancer treatment. Although our data on the effects of 25(OH)D3 and its metabolites on OPC are limited, they provide some evidence from MR analysis that could impact innovative approaches targeting vitamin D or its metabolites for the effective management of OPC. Further well-designed clinical trials should be conducted to better understand the role of vitamin D in OPC therapy.

## Conclusions

Our study does not support the observational association of the levels of 25(OH)D3 and its metabolites and the risk of developing OPC and is consistent with evidence that a causal, clinically relevant protective effect of greater 25(OH)D levels against the development of OPC is unlikely.

## Supplementary Material





## Data Availability

All the data generated or analyzed during this study are included in this published article and its supplementary information files.
