# Supplementary material for: The causal associations of 25(OH)D and its metabolites with oropharyngeal cancer risk: a Mendelian randomization study
Source: Acta Odontol Scand. 2025 Jul 17;84:44053. doi: 10.2340/aos.v84.44053 (PMC12362939; doi:10.2340/aos.v84.44053)
Supplement: Supplementary file 1 [file AOS-84-44053-s1.pdf]

Supplementary+Data+1

	SNP	effect_allele.exposure	other_allele.exposure	effect_allele.outcome	other_allele.outcome	beta.exposure	beta.outcome	eaf.exposure	eaf.outcome	remove	palindromic	ambiguous	id.outcome	chr	pos	se.outcome	samplesize.outcome	pval.outcome	outcome	originalname.outcome	outcome.deprecated
1	rs116970203	G	A	G	A	0.372	-0.307	0.98	0.98	FALSE	FALSE	FALSE	ieu-b-96	11	14876718	0.175	4018	0.0790133	Oropharyngeal cancer || id:ieu-b-96	Oropharyngeal cancer	Oropharyngeal cancer || ||
2	rs13084927	C	A	C	A	0.055	-0.023	0.83	0.824	FALSE	FALSE	FALSE	ieu-b-96	3	72709792	0.076	4018	0.76476	Oropharyngeal cancer || id:ieu-b-96	Oropharyngeal cancer	Oropharyngeal cancer || ||
3	rs17216707	T	C	T	C	0.074	-0.056	0.8	0.787	FALSE	FALSE	FALSE	ieu-b-96	20	52732362	0.076	4018	0.4545	Oropharyngeal cancer || id:ieu-b-96	Oropharyngeal cancer	Oropharyngeal cancer || ||
4	rs28364617	G	T	G	T	0.127	-0.052	0.71	0.722	FALSE	FALSE	FALSE	ieu-b-96	11	71159764	0.062	4018	0.400214	Oropharyngeal cancer || id:ieu-b-96	Oropharyngeal cancer	Oropharyngeal cancer || ||
5	rs3819817	C	T	C	T	0.058	-0.067	0.45	0.474	FALSE	FALSE	FALSE	ieu-b-96	12	96378771	0.056	4018	0.228443	Oropharyngeal cancer || id:ieu-b-96	Oropharyngeal cancer	Oropharyngeal cancer || ||
6	rs9304669	T	C	T	C	0.054	0.105	0.16	0.169	FALSE	FALSE	FALSE	ieu-b-96	19	48384385	0.071	4018	0.140563	Oropharyngeal cancer || id:ieu-b-96	Oropharyngeal cancer	Oropharyngeal cancer || ||
7	rs10277163	G	A	G	A	-0.0143336	-0.102	0.254715	0.246	FALSE	FALSE	FALSE	ieu-b-96	7	21569089	0.065	4018	0.114338	Oropharyngeal cancer || id:ieu-b-96	Oropharyngeal cancer	Oropharyngeal cancer || ||
8	rs1038165	T	C	T	C	0.0115149	-0.112	0.579451	0.546	FALSE	FALSE	FALSE	ieu-b-96	12	68665940	0.056	4018	0.0437472	Oropharyngeal cancer || id:ieu-b-96	Oropharyngeal cancer	Oropharyngeal cancer || ||
9	rs1042034	T	C	T	C	-0.0151254	-0.03	0.792138	0.777	FALSE	FALSE	FALSE	ieu-b-96	2	21226560	0.066	4018	0.643324	Oropharyngeal cancer || id:ieu-b-96	Oropharyngeal cancer	Oropharyngeal cancer || ||
10	rs10438978	C	T	C	T	-0.0172243	0.035	0.820277	0.849	FALSE	FALSE	FALSE	ieu-b-96	18	47158186	0.076	4018	0.642808	Oropharyngeal cancer || id:ieu-b-96	Oropharyngeal cancer	Oropharyngeal cancer || ||
11	rs1047891	A	C	A	C	-0.0133984	-0.022	0.317078	0.301	FALSE	FALSE	FALSE	ieu-b-96	2	211540507	0.059	4015	0.7103	Oropharyngeal cancer || id:ieu-b-96	Oropharyngeal cancer	Oropharyngeal cancer || ||
12	rs1048328	A	G	A	G	0.0313497	-0.017	0.0797967	0.09	FALSE	FALSE	FALSE	ieu-b-96	19	51527364	0.098	4014	0.8635	Oropharyngeal cancer || id:ieu-b-96	Oropharyngeal cancer	Oropharyngeal cancer || ||
13	rs10859995	C	T	C	T	-0.0436264	0.067	0.579841	0.526	FALSE	FALSE	FALSE	ieu-b-96	12	96378771	0.056	4018	0.228443	Oropharyngeal cancer || id:ieu-b-96	Oropharyngeal cancer	Oropharyngeal cancer || ||
14	rs11023159	C	T	C	T	0.0482117	-0.183	0.0325143	0.036	FALSE	FALSE	FALSE	ieu-b-96	11	14262063	0.162	4018	0.258803	Oropharyngeal cancer || id:ieu-b-96	Oropharyngeal cancer	Oropharyngeal cancer || ||
15	rs11076175	G	A	G	A	0.0229033	-0.028	0.175666	0.174	FALSE	FALSE	FALSE	ieu-b-96	16	57006378	0.073	4018	0.692786	Oropharyngeal cancer || id:ieu-b-96	Oropharyngeal cancer	Oropharyngeal cancer || ||
16	rs11207969	G	A	G	A	0.0209396	0.045	0.351365	0.314	FALSE	FALSE	FALSE	ieu-b-96	1	62911751	0.06	4018	0.451376	Oropharyngeal cancer || id:ieu-b-96	Oropharyngeal cancer	Oropharyngeal cancer || ||
17	rs11264361	G	T	G	T	0.0174875	0.071	0.251408	0.232	FALSE	FALSE	FALSE	ieu-b-96	1	155289545	0.065	4018	0.277025	Oropharyngeal cancer || id:ieu-b-96	Oropharyngeal cancer	Oropharyngeal cancer || ||
18	rs1128535	T	C	T	C	0.0164131	0.002	0.500049	0.488	FALSE	FALSE	FALSE	ieu-b-96	3	49866392	0.055	4018	0.976018	Oropharyngeal cancer || id:ieu-b-96	Oropharyngeal cancer	Oropharyngeal cancer || ||
19	rs115288876	A	G	A	G	0.0788065	-0.009	0.0433211	0.035	FALSE	FALSE	FALSE	ieu-b-96	1	152000117	0.152	4018	0.953919	Oropharyngeal cancer || id:ieu-b-96	Oropharyngeal cancer	Oropharyngeal cancer || ||
20	rs11542462	A	G	A	G	-0.0247803	-0.05	0.133511	0.131	FALSE	FALSE	FALSE	ieu-b-96	16	82033810	0.082	4016	0.5447	Oropharyngeal cancer || id:ieu-b-96	Oropharyngeal cancer	Oropharyngeal cancer || ||
21	rs11726886	A	C	A	C	-0.0536726	-0.021	0.290783	0.284	FALSE	FALSE	FALSE	ieu-b-96	4	72822599	0.061	4015	0.7204	Oropharyngeal cancer || id:ieu-b-96	Oropharyngeal cancer	Oropharyngeal cancer || ||
22	rs11791258	A	G	A	G	0.0140808	-0.053	0.191072	0.184	FALSE	FALSE	FALSE	ieu-b-96	9	107632644	0.078	4018	0.491585	Oropharyngeal cancer || id:ieu-b-96	Oropharyngeal cancer	Oropharyngeal cancer || ||
23	rs11867297	T	C	T	C	0.0135432	-0.043	0.38533	0.41	FALSE	FALSE	FALSE	ieu-b-96	17	66433493	0.056	4018	0.438995	Oropharyngeal cancer || id:ieu-b-96	Oropharyngeal cancer	Oropharyngeal cancer || ||
24	rs12056768	G	T	G	T	-0.0231961	0.001	0.584044	0.591	FALSE	FALSE	FALSE	ieu-b-96	8	116988527	0.055	4018	0.979847	Oropharyngeal cancer || id:ieu-b-96	Oropharyngeal cancer	Oropharyngeal cancer || ||
25	rs12153819	T	C	T	C	-0.0178178	-0.011	0.123154	0.118	FALSE	FALSE	FALSE	ieu-b-96	6	83773049	0.084	4018	0.895779	Oropharyngeal cancer || id:ieu-b-96	Oropharyngeal cancer	Oropharyngeal cancer || ||
26	rs12283049	G	A	G	A	-0.0564566	-0.087	0.234433	0.249	FALSE	FALSE	FALSE	ieu-b-96	11	14690192	0.066	4016	0.1908	Oropharyngeal cancer || id:ieu-b-96	Oropharyngeal cancer	Oropharyngeal cancer || ||
27	rs12324720	A	G	A	G	-0.0149159	0.044	0.174629	0.192	FALSE	FALSE	FALSE	ieu-b-96	15	64092140	0.072	4018	0.537464	Oropharyngeal cancer || id:ieu-b-96	Oropharyngeal cancer	Oropharyngeal cancer || ||
28	rs12462826	A	G	A	G	-0.0132119	0.016	0.369566	0.367	FALSE	FALSE	FALSE	ieu-b-96	19	11957001	0.056	4018	0.781712	Oropharyngeal cancer || id:ieu-b-96	Oropharyngeal cancer	Oropharyngeal cancer || ||
29	rs12501515	A	G	A	G	-0.078957	0.028	0.58966	0.57	FALSE	FALSE	FALSE	ieu-b-96	4	72592838	0.056	4018	0.615191	Oropharyngeal cancer || id:ieu-b-96	Oropharyngeal cancer	Oropharyngeal cancer || ||
30	rs1260326	C	T	C	T	0.0197194	-0.05	0.603925	0.574	FALSE	FALSE	FALSE	ieu-b-96	2	27730940	0.057	4018	0.372317	Oropharyngeal cancer || id:ieu-b-96	Oropharyngeal cancer	Oropharyngeal cancer || ||
31	rs12775091	T	C	T	C	0.0155618	0.008	0.213441	0.205	FALSE	FALSE	FALSE	ieu-b-96	10	91543551	0.068	4018	0.911142	Oropharyngeal cancer || id:ieu-b-96	Oropharyngeal cancer	Oropharyngeal cancer || ||
32	rs13076508	C	T	C	T	0.0250544	-0.174	0.0535034	0.059	FALSE	FALSE	FALSE	ieu-b-96	3	52407805	0.127	4018	0.170482	Oropharyngeal cancer || id:ieu-b-96	Oropharyngeal cancer	Oropharyngeal cancer || ||
33	rs13108245	G	A	G	A	-0.0122246	-0.017	0.386601	0.403	FALSE	FALSE	FALSE	ieu-b-96	4	57790205	0.056	4018	0.761083	Oropharyngeal cancer || id:ieu-b-96	Oropharyngeal cancer	Oropharyngeal cancer || ||
34	rs1321247	T	A	T	A	-0.0221847	-0.124	0.101578	0.085	FALSE	TRUE	FALSE	ieu-b-96	6	25662873	0.097	4018	0.199556	Oropharyngeal cancer || id:ieu-b-96	Oropharyngeal cancer	Oropharyngeal cancer || ||
35	rs13294734	T	C	T	C	0.012568	-0.076	0.466128	0.469	FALSE	FALSE	FALSE	ieu-b-96	9	80710910	0.055	4018	0.167487	Oropharyngeal cancer || id:ieu-b-96	Oropharyngeal cancer	Oropharyngeal cancer || ||
36	rs1343776	A	G	A	G	0.0180762	0.046	0.22133	0.222	FALSE	FALSE	FALSE	ieu-b-96	1	41757718	0.066	4018	0.484424	Oropharyngeal cancer || id:ieu-b-96	Oropharyngeal cancer	Oropharyngeal cancer || ||
37	rs138335	G	C	G	C	-0.0137663	-0.006	0.658587	0.648	FALSE	TRUE	FALSE	ieu-b-96	22	41227086	0.058	4018	0.922777	Oropharyngeal cancer || id:ieu-b-96	Oropharyngeal cancer	Oropharyngeal cancer || ||
38	rs1384687	A	G	A	G	-0.0168656	0.093	0.132271	0.142	FALSE	FALSE	FALSE	ieu-b-96	8	61525963	0.081	4018	0.252178	Oropharyngeal cancer || id:ieu-b-96	Oropharyngeal cancer	Oropharyngeal cancer || ||
39	rs142004400	C	A	C	A	-0.0310034	-0.008	0.0342066	0.035	FALSE	FALSE	FALSE	ieu-b-96	14	50829560	0.157	4018	0.960844	Oropharyngeal cancer || id:ieu-b-96	Oropharyngeal cancer	Oropharyngeal cancer || ||
40	rs142158911	A	G	A	G	0.026284	0.083	0.111826	0.113	FALSE	FALSE	FALSE	ieu-b-96	19	11190534	0.088	4018	0.344115	Oropharyngeal cancer || id:ieu-b-96	Oropharyngeal cancer	Oropharyngeal cancer || ||
41	rs144965707	A	G	A	G	-0.0348143	0.009	0.0618296	0.075	FALSE	FALSE	FALSE	ieu-b-96	11	14059511	0.11	4018	0.934741	Oropharyngeal cancer || id:ieu-b-96	Oropharyngeal cancer	Oropharyngeal cancer || ||
42	rs1627043	C	G	C	G	-0.0486441	0.113	0.0332414	0.035	FALSE	TRUE	FALSE	ieu-b-96	11	71110175	0.159	4018	0.478105	Oropharyngeal cancer || id:ieu-b-96	Oropharyngeal cancer	Oropharyngeal cancer || ||
43	rs1684600	T	C	T	C	-0.0125301	0.011	0.298695	0.301	FALSE	FALSE	FALSE	ieu-b-96	16	4594671	0.063	4018	0.85898	Oropharyngeal cancer || id:ieu-b-96	Oropharyngeal cancer	Oropharyngeal cancer || ||
44	rs17207784	C	T	C	T	-0.0134939	-0.111	0.324195	0.342	FALSE	FALSE	FALSE	ieu-b-96	6	22768668	0.059	4018	0.0609649	Oropharyngeal cancer || id:ieu-b-96	Oropharyngeal cancer	Oropharyngeal cancer || ||
45	rs17473257	A	G	A	G	-0.0611372	0.017	0.0172466	0.02	FALSE	FALSE	FALSE	ieu-b-96	11	14283186	0.197	4018	0.931549	Oropharyngeal cancer || id:ieu-b-96	Oropharyngeal cancer	Oropharyngeal cancer || ||
46	rs1800588	T	C	T	C	-0.0305021	0.077	0.214964	0.205	FALSE	FALSE	FALSE	ieu-b-96	15	58730498	0.069	4018	0.265656	Oropharyngeal cancer || id:ieu-b-96	Oropharyngeal cancer	Oropharyngeal cancer || ||
47	rs1841850	C	A	C	A	0.0304395	-0.067	0.117085	0.123	FALSE	FALSE	FALSE	ieu-b-96	20	52718555	0.089	4018	0.452123	Oropharyngeal cancer || id:ieu-b-96	Oropharyngeal cancer	Oropharyngeal cancer || ||
48	rs1858889	C	A	C	A	0.0134514	-0.046	0.502532	0.508	FALSE	FALSE	FALSE	ieu-b-96	7	107117447	0.055	4018	0.404257	Oropharyngeal cancer || id:ieu-b-96	Oropharyngeal cancer	Oropharyngeal cancer || ||
49	rs1871395	G	A	G	A	-0.0203733	0.054	0.152679	0.162	FALSE	FALSE	FALSE	ieu-b-96	12	21352315	0.076	4016	0.4687	Oropharyngeal cancer || id:ieu-b-96	Oropharyngeal cancer	Oropharyngeal cancer || ||
50	rs1949633	C	T	C	T	0.0114162	-0.023	0.605759	0.587	FALSE	FALSE	FALSE	ieu-b-96	3	153758806	0.056	4018	0.672433	Oropharyngeal cancer || id:ieu-b-96	Oropharyngeal cancer	Oropharyngeal cancer || ||
51	rs2037511	A	G	A	G	0.0176624	0.053	0.165924	0.188	FALSE	FALSE	FALSE	ieu-b-96	18	61366207	0.07	4016	0.4493	Oropharyngeal cancer || id:ieu-b-96	Oropharyngeal cancer	Oropharyngeal cancer || ||
52	rs2074735	C	G	C	G	0.0292667	0.023	0.0648365	0.075	FALSE	TRUE	FALSE	ieu-b-96	22	31535872	0.107	4015	0.835	Oropharyngeal cancer || id:ieu-b-96	Oropharyngeal cancer	Oropharyngeal cancer || ||
53	rs2171427	A	G	A	G	-0.0165489	0.095	0.156491	0.155	FALSE	FALSE	FALSE	ieu-b-96	12	24822366	0.076	4018	0.211672	Oropharyngeal cancer || id:ieu-b-96	Oropharyngeal cancer	Oropharyngeal cancer || ||
54	rs2229742	C	G	C	G	-0.0249837	0.057	0.104659	0.102	FALSE	TRUE	FALSE	ieu-b-96	21	16339172	0.09	4018	0.52625	Oropharyngeal cancer || id:ieu-b-96	Oropharyngeal cancer	Oropharyngeal cancer || ||
55	rs2245133	C	T	C	T	-0.0212917	-0.027	0.164339	0.155	FALSE	FALSE	FALSE	ieu-b-96	6	131931092	0.075	4018	0.710551	Oropharyngeal cancer || id:ieu-b-96	Oropharyngeal cancer	Oropharyngeal cancer || ||
56	rs2297991	C	T	C	T	0.0127547	0.071	0.718478	0.689	FALSE	FALSE	FALSE	ieu-b-96	10	113913222	0.06	4018	0.239499	Oropharyngeal cancer || id:ieu-b-96	Oropharyngeal cancer	Oropharyngeal cancer || ||
57	rs2398113	G	A	G	A	-0.0117606	0.075	0.423526	0.408	FALSE	FALSE	FALSE	ieu-b-96	10	10078742	0.061	4018	0.218808	Oropharyngeal cancer || id:ieu-b-96	Oropharyngeal cancer	Oropharyngeal cancer || ||
58	rs2494429	G	A	G	A	-0.0148459	-0.03	0.822999	0.82	FALSE	FALSE	FALSE	ieu-b-96	1	2339395	0.078	4018	0.693897	Oropharyngeal cancer || id:ieu-b-96	Oropharyngeal cancer	Oropharyngeal cancer || ||
59	rs2511279	G	C	G	C	0.0981721	0.062	0.960373	0.95	FALSE	TRUE	FALSE	ieu-b-96	11	71130419	0.136	4018	0.647558	Oropharyngeal cancer || id:ieu-b-96	Oropharyngeal cancer	Oropharyngeal cancer || ||
60	rs2595644	T	G	T	G	-0.0122625	-0.052	0.384889	0.396	FALSE	FALSE	FALSE	ieu-b-96	7	43980540	0.057	4018	0.353771	Oropharyngeal cancer || id:ieu-b-96	Oropharyngeal cancer	Oropharyngeal cancer || ||
61	rs2710651	A	G	A	G	-0.0115892	-0.001	0.526101	0.504	FALSE	FALSE	FALSE	ieu-b-96	2	63166379	0.06	4016	0.988	Oropharyngeal cancer || id:ieu-b-96	Oropharyngeal cancer	Oropharyngeal cancer || ||
62	rs2756119	A	G	A	G	0.0121434	-0.01	0.38143	0.374	FALSE	FALSE	FALSE	ieu-b-96	14	104001517	0.058	4018	0.862625	Oropharyngeal cancer || id:ieu-b-96	Oropharyngeal cancer	Oropharyngeal cancer || ||
63	rs2807834	G	T	G	T	-0.0150625	0.074	0.68514	0.678	FALSE	FALSE	FALSE	ieu-b-96	1	220970593	0.06	4018	0.216766	Oropharyngeal cancer || id:ieu-b-96	Oropharyngeal cancer	Oropharyngeal cancer || ||
64	rs28435470	A	G	A	G	-0.0118696	-0.054	0.663204	0.703	FALSE	FALSE	FALSE	ieu-b-96	12	133076439	0.062	4018	0.373249	Oropharyngeal cancer || id:ieu-b-96	Oropharyngeal cancer	Oropharyngeal cancer || ||
65	rs2847500	A	G	A	G	-0.022548	-0.227	0.123204	0.135	FALSE	FALSE	FALSE	ieu-b-96	11	120114421	0.091	4018	0.0128535	Oropharyngeal cancer || id:ieu-b-96	Oropharyngeal cancer	Oropharyngeal cancer || ||
66	rs290400	A	G	A	G	-0.0130967	0.021	0.665472	0.686	FALSE	FALSE	FALSE	ieu-b-96	20	52698179	0.06	4018	0.726718	Oropharyngeal cancer || id:ieu-b-96	Oropharyngeal cancer	Oropharyngeal cancer || ||
67	rs3114045	C	T	C	T	-0.0221737	0.241	0.866137	0.842	FALSE	FALSE	FALSE	ieu-b-96	4	100252560	0.081	4018	0.00290088	Oropharyngeal cancer || id:ieu-b-96	Oropharyngeal cancer	Oropharyngeal cancer || ||
68	rs325393	T	G	T	G	-0.0136497	-0.003	0.278218	0.274	FALSE	FALSE	FALSE	ieu-b-96	15	100229260	0.062	4018	0.961257	Oropharyngeal cancer || id:ieu-b-96	Oropharyngeal cancer	Oropharyngeal cancer || ||
69	rs34186890	G	A	G	A	-0.0156853	0.038	0.259637	0.257	FALSE	FALSE	FALSE	ieu-b-96	3	141720712	0.063	4018	0.544353	Oropharyngeal cancer || id:ieu-b-96	Oropharyngeal cancer	Oropharyngeal cancer || ||
70	rs34726834	T	C	T	C	0.014013	0.075	0.252209	0.245	FALSE	FALSE	FALSE	ieu-b-96	8	25889606	0.063	4018	0.234188	Oropharyngeal cancer || id:ieu-b-96	Oropharyngeal cancer	Oropharyngeal cancer || ||
71	rs35270497	T	C	T	C	0.0156723	-0.011	0.176236	0.152	FALSE	FALSE	FALSE	ieu-b-96	2	38259872	0.076	4018	0.885731	Oropharyngeal cancer || id:ieu-b-96	Oropharyngeal cancer	Oropharyngeal cancer || ||
72	rs35823191	C	T	C	T	-0.0232636	0.005	0.342049	0.364	FALSE	FALSE	FALSE	ieu-b-96	1	17560123	0.057	4018	0.924843	Oropharyngeal cancer || id:ieu-b-96	Oropharyngeal cancer	Oropharyngeal cancer || ||
73	rs3732220	A	G	A	G	-0.0478406	-0.074	0.0852646	0.096	FALSE	FALSE	FALSE	ieu-b-96	2	234627048	0.096	4018	0.441663	Oropharyngeal cancer || id:ieu-b-96	Oropharyngeal cancer	Oropharyngeal cancer || ||
74	rs3829251	A	G	A	G	-0.114453	0.076	0.133277	0.176	FALSE	FALSE	FALSE	ieu-b-96	11	71194559	0.074	4016	0.307	Oropharyngeal cancer || id:ieu-b-96	Oropharyngeal cancer	Oropharyngeal cancer || ||
75	rs4147536	C	A	C	A	-0.0148038	-0.021	0.788613	0.797	FALSE	FALSE	FALSE	ieu-b-96	4	100239112	0.069	4018	0.76375	Oropharyngeal cancer || id:ieu-b-96	Oropharyngeal cancer	Oropharyngeal cancer || ||
76	rs4348160	G	T	G	T	-0.0258401	0.022	0.326968	0.35	FALSE	FALSE	FALSE	ieu-b-96	4	70017531	0.058	4018	0.70928	Oropharyngeal cancer || id:ieu-b-96	Oropharyngeal cancer	Oropharyngeal cancer || ||
77	rs4364259	A	G	A	G	0.0172408	-0.038	0.198665	0.207	FALSE	FALSE	FALSE	ieu-b-96	4	15892159	0.073	4018	0.608213	Oropharyngeal cancer || id:ieu-b-96	Oropharyngeal cancer	Oropharyngeal cancer || ||
78	rs4420638	G	A	G	A	-0.0192973	-0.14	0.176831	0.167	FALSE	FALSE	FALSE	ieu-b-96	19	45422946	0.075	3998	0.0629796	Oropharyngeal cancer || id:ieu-b-96	Oropharyngeal cancer	Oropharyngeal cancer || ||
79	rs4580037	C	A	C	A	-0.0135627	-0.066	0.285578	0.288	FALSE	FALSE	FALSE	ieu-b-96	13	55702646	0.063	4018	0.287918	Oropharyngeal cancer || id:ieu-b-96	Oropharyngeal cancer	Oropharyngeal cancer || ||
80	rs512083	C	T	C	T	0.0122172	0.095	0.462488	0.458	FALSE	FALSE	FALSE	ieu-b-96	1	46027355	0.055	4018	0.0818446	Oropharyngeal cancer || id:ieu-b-96	Oropharyngeal cancer	Oropharyngeal cancer || ||
81	rs57601828	T	A	T	A	0.011542	-0.158	0.394508	0.371	FALSE	TRUE	FALSE	ieu-b-96	12	93204367	0.058	4018	0.00664385	Oropharyngeal cancer || id:ieu-b-96	Oropharyngeal cancer	Oropharyngeal cancer || ||
82	rs5770794	T	C	T	C	-0.0133141	-0.045	0.3143	0.319	FALSE	FALSE	FALSE	ieu-b-96	22	50880781	0.06	4018	0.450941	Oropharyngeal cancer || id:ieu-b-96	Oropharyngeal cancer	Oropharyngeal cancer || ||
83	rs6129648	G	A	G	A	0.0140631	0	0.379837	0.349	FALSE	FALSE	FALSE	ieu-b-96	20	39231118	0.058	4018	0.996688	Oropharyngeal cancer || id:ieu-b-96	Oropharyngeal cancer	Oropharyngeal cancer || ||
84	rs61698755	C	T	C	T	-0.011465	-0.031	0.560041	0.551	FALSE	FALSE	FALSE	ieu-b-96	17	79257880	0.056	4018	0.5707	Oropharyngeal cancer || id:ieu-b-96	Oropharyngeal cancer	Oropharyngeal cancer || ||
85	rs61813875	G	C	G	C	0.0821291	0.001	0.0248209	0.019	FALSE	TRUE	FALSE	ieu-b-96	1	152536650	0.21	4018	0.997034	Oropharyngeal cancer || id:ieu-b-96	Oropharyngeal cancer	Oropharyngeal cancer || ||
86	rs61887421	C	T	C	T	-0.036726	0.006	0.0300871	0.031	FALSE	FALSE	FALSE	ieu-b-96	11	70949673	0.17	4018	0.969943	Oropharyngeal cancer || id:ieu-b-96	Oropharyngeal cancer	Oropharyngeal cancer || ||
87	rs62007299	A	G	A	G	-0.0124205	0.011	0.712958	0.693	FALSE	FALSE	FALSE	ieu-b-96	15	77711719	0.059	4018	0.847476	Oropharyngeal cancer || id:ieu-b-96	Oropharyngeal cancer	Oropharyngeal cancer || ||
88	rs62129966	A	C	A	C	0.0611636	0.096	0.160854	0.163	FALSE	FALSE	FALSE	ieu-b-96	19	48374950	0.072	4018	0.181774	Oropharyngeal cancer || id:ieu-b-96	Oropharyngeal cancer	Oropharyngeal cancer || ||
89	rs635634	T	C	T	C	-0.0150476	-0.002	0.186573	0.198	FALSE	FALSE	FALSE	ieu-b-96	9	136155000	0.069	4018	0.98174	Oropharyngeal cancer || id:ieu-b-96	Oropharyngeal cancer	Oropharyngeal cancer || ||
90	rs6438900	G	C	G	C	0.0150486	-0.034	0.256034	0.255	FALSE	TRUE	FALSE	ieu-b-96	3	125148287	0.063	4018	0.592561	Oropharyngeal cancer || id:ieu-b-96	Oropharyngeal cancer	Oropharyngeal cancer || ||
91	rs6672758	T	C	T	C	0.0162478	-0.06	0.80023	0.794	FALSE	FALSE	FALSE	ieu-b-96	1	230303512	0.068	4018	0.380273	Oropharyngeal cancer || id:ieu-b-96	Oropharyngeal cancer	Oropharyngeal cancer || ||
92	rs6834488	T	C	T	C	-0.01445	-0.053	0.422833	0.408	FALSE	FALSE	FALSE	ieu-b-96	4	88178919	0.055	4018	0.335657	Oropharyngeal cancer || id:ieu-b-96	Oropharyngeal cancer	Oropharyngeal cancer || ||
93	rs71599974	G	A	G	A	0.0257378	0.058	0.148048	0.129	FALSE	FALSE	FALSE	ieu-b-96	4	71765339	0.089	4018	0.512675	Oropharyngeal cancer || id:ieu-b-96	Oropharyngeal cancer	Oropharyngeal cancer || ||
94	rs727857	A	G	A	G	-0.0120548	-0.104	0.61181	0.616	FALSE	FALSE	FALSE	ieu-b-96	2	58981967	0.057	4018	0.0648291	Oropharyngeal cancer || id:ieu-b-96	Oropharyngeal cancer	Oropharyngeal cancer || ||
95	rs733454	T	C	T	C	0.0188545	0.128	0.0991663	0.076	FALSE	FALSE	FALSE	ieu-b-96	11	76477721	0.099	4018	0.197334	Oropharyngeal cancer || id:ieu-b-96	Oropharyngeal cancer	Oropharyngeal cancer || ||
96	rs742493	C	T	C	T	0.0183528	0.041	0.112871	0.119	FALSE	FALSE	FALSE	ieu-b-96	6	40998167	0.084	4018	0.625492	Oropharyngeal cancer || id:ieu-b-96	Oropharyngeal cancer	Oropharyngeal cancer || ||
97	rs7528419	G	A	G	A	0.0215389	0.001	0.22441	0.218	FALSE	FALSE	FALSE	ieu-b-96	1	109817192	0.066	4006	0.9887	Oropharyngeal cancer || id:ieu-b-96	Oropharyngeal cancer	Oropharyngeal cancer || ||
98	rs7569755	A	G	A	G	0.0136395	-0.053	0.288618	0.281	FALSE	FALSE	FALSE	ieu-b-96	2	118648261	0.061	4018	0.381991	Oropharyngeal cancer || id:ieu-b-96	Oropharyngeal cancer	Oropharyngeal cancer || ||
99	rs7580771	T	G	T	G	-0.0165625	-0.08	0.175983	0.194	FALSE	FALSE	FALSE	ieu-b-96	2	101428119	0.07	4018	0.253967	Oropharyngeal cancer || id:ieu-b-96	Oropharyngeal cancer	Oropharyngeal cancer || ||
100	rs7652808	G	T	G	T	-0.0212858	0.035	0.649492	0.637	FALSE	FALSE	FALSE	ieu-b-96	3	85604320	0.059	4018	0.544899	Oropharyngeal cancer || id:ieu-b-96	Oropharyngeal cancer	Oropharyngeal cancer || ||
101	rs7712001	G	T	G	T	0.011939	0.038	0.440176	0.418	FALSE	FALSE	FALSE	ieu-b-96	5	148020950	0.057	4018	0.50375	Oropharyngeal cancer || id:ieu-b-96	Oropharyngeal cancer	Oropharyngeal cancer || ||
102	rs7784802	T	A	T	A	0.0133202	-0.04	0.358913	0.273	FALSE	TRUE	FALSE	ieu-b-96	7	64020652	0.062	4018	0.516312	Oropharyngeal cancer || id:ieu-b-96	Oropharyngeal cancer	Oropharyngeal cancer || ||
103	rs77924615	A	G	A	G	-0.0152475	-0.082	0.194374	0.194	FALSE	FALSE	FALSE	ieu-b-96	16	20392332	0.073	4018	0.261782	Oropharyngeal cancer || id:ieu-b-96	Oropharyngeal cancer	Oropharyngeal cancer || ||
104	rs77960347	G	A	G	A	-0.0525688	0.054	0.0127013	0.014	FALSE	FALSE	FALSE	ieu-b-96	18	47109955	0.246	4018	0.825918	Oropharyngeal cancer || id:ieu-b-96	Oropharyngeal cancer	Oropharyngeal cancer || ||
105	rs78649910	A	T	A	T	-0.0191377	0.094	0.105728	0.102	FALSE	TRUE	FALSE	ieu-b-96	4	3482213	0.09	4018	0.293636	Oropharyngeal cancer || id:ieu-b-96	Oropharyngeal cancer	Oropharyngeal cancer || ||
106	rs7955128	T	A	T	A	0.0130617	0.069	0.52053	0.513	FALSE	TRUE	TRUE	ieu-b-96	12	38684121	0.057	4018	0.219712	Oropharyngeal cancer || id:ieu-b-96	Oropharyngeal cancer	Oropharyngeal cancer || ||
107	rs8018720	C	G	C	G	-0.0344962	-0.078	0.823508	0.831	FALSE	TRUE	FALSE	ieu-b-96	14	39556185	0.073	4018	0.289667	Oropharyngeal cancer || id:ieu-b-96	Oropharyngeal cancer	Oropharyngeal cancer || ||
108	rs804281	G	A	G	A	0.015895	0.07	0.583498	0.578	FALSE	FALSE	FALSE	ieu-b-96	8	11611865	0.073	4018	0.334407	Oropharyngeal cancer || id:ieu-b-96	Oropharyngeal cancer	Oropharyngeal cancer || ||
109	rs8107974	T	A	T	A	0.0355672	0.109	0.0763013	0.065	FALSE	TRUE	FALSE	ieu-b-96	19	19388500	0.107	4018	0.305053	Oropharyngeal cancer || id:ieu-b-96	Oropharyngeal cancer	Oropharyngeal cancer || ||
110	rs8121940	G	C	G	C	-0.0435612	0.093	0.19762	0.221	FALSE	TRUE	FALSE	ieu-b-96	20	52742306	0.075	4018	0.21599	Oropharyngeal cancer || id:ieu-b-96	Oropharyngeal cancer	Oropharyngeal cancer || ||
111	rs9375037	C	A	C	A	0.0117059	0.037	0.443168	0.424	FALSE	FALSE	FALSE	ieu-b-96	6	121856794	0.056	4018	0.507756	Oropharyngeal cancer || id:ieu-b-96	Oropharyngeal cancer	Oropharyngeal cancer || ||
112	rs9409266	A	G	A	G	-0.0167771	-0.056	0.862222	0.883	FALSE	FALSE	FALSE	ieu-b-96	9	125824055	0.087	4018	0.522186	Oropharyngeal cancer || id:ieu-b-96	Oropharyngeal cancer	Oropharyngeal cancer || ||
113	rs964184	C	G	C	G	0.0406845	-0.078	0.867234	0.862	FALSE	TRUE	FALSE	ieu-b-96	11	116648917	0.08	4015	0.3319	Oropharyngeal cancer || id:ieu-b-96	Oropharyngeal cancer	Oropharyngeal cancer || ||
114	rs9847248	A	G	A	G	-0.0123077	-0.064	0.712969	0.696	FALSE	FALSE	FALSE	ieu-b-96	3	18804655	0.06	4018	0.285604	Oropharyngeal cancer || id:ieu-b-96	Oropharyngeal cancer	Oropharyngeal cancer || ||
115	rs986649	G	A	G	A	0.0128637	-0.004	0.321628	0.33	FALSE	FALSE	FALSE	ieu-b-96	5	118668050	0.058	4014	0.9437	Oropharyngeal cancer || id:ieu-b-96	Oropharyngeal cancer	Oropharyngeal cancer || ||
116	rs9946771	T	C	T	C	-0.0233992	-0.195	0.0663426	0.073	FALSE	FALSE	FALSE	ieu-b-96	18	28918628	0.109	4018	0.0743944	Oropharyngeal cancer || id:ieu-b-96	Oropharyngeal cancer	Oropharyngeal cancer || ||
117	rs11172066	T	A	T	A	0.166	-0.071	0.14	0.15	FALSE	TRUE	FALSE	ieu-b-96	12	57319491	0.081	4018	0.382377	Oropharyngeal cancer || id:ieu-b-96	Oropharyngeal cancer	Oropharyngeal cancer || ||
118	rs28364617	G	T	G	T	0.131	-0.052	0.72	0.722	FALSE	FALSE	FALSE	ieu-b-96	11	71159764	0.062	4018	0.400214	Oropharyngeal cancer || id:ieu-b-96	Oropharyngeal cancer	Oropharyngeal cancer || ||
119	rs10277163	G	A	G	A	-0.0143336	-0.048	0.254715	0.243	FALSE	FALSE	FALSE	ieu-b-97	7	21569089	0.063	3448	0.439048	Oropharyngeal cancer || id:ieu-b-97	Oropharyngeal cancer	Oropharyngeal cancer || ||
120	rs1038165	T	C	T	C	0.0115149	-0.019	0.579451	0.549	FALSE	FALSE	FALSE	ieu-b-97	12	68665940	0.056	3448	0.7267	Oropharyngeal cancer || id:ieu-b-97	Oropharyngeal cancer	Oropharyngeal cancer || ||
121	rs1042034	T	C	T	C	-0.0151254	-0.012	0.792138	0.778	FALSE	FALSE	FALSE	ieu-b-97	2	21226560	0.064	3448	0.854878	Oropharyngeal cancer || id:ieu-b-97	Oropharyngeal cancer	Oropharyngeal cancer || ||
122	rs10438978	C	T	C	T	-0.0172243	-0.002	0.820277	0.837	FALSE	FALSE	FALSE	ieu-b-97	18	47158186	0.073	3448	0.974982	Oropharyngeal cancer || id:ieu-b-97	Oropharyngeal cancer	Oropharyngeal cancer || ||
123	rs1047891	A	C	A	C	-0.0133984	0.171	0.317078	0.295	FALSE	FALSE	FALSE	ieu-b-97	2	211540507	0.059	3448	0.00403999	Oropharyngeal cancer || id:ieu-b-97	Oropharyngeal cancer	Oropharyngeal cancer || ||
124	rs1048328	A	G	A	G	0.0313497	0.009	0.0797967	0.09	FALSE	FALSE	FALSE	ieu-b-97	19	51527364	0.096	3448	0.9235	Oropharyngeal cancer || id:ieu-b-97	Oropharyngeal cancer	Oropharyngeal cancer || ||
125	rs10859995	C	T	C	T	-0.0436264	-0.005	0.579841	0.546	FALSE	FALSE	FALSE	ieu-b-97	12	96378771	0.056	3448	0.933661	Oropharyngeal cancer || id:ieu-b-97	Oropharyngeal cancer	Oropharyngeal cancer || ||
126	rs11023159	C	T	C	T	0.0482117	0.053	0.0325143	0.03	FALSE	FALSE	FALSE	ieu-b-97	11	14262063	0.162	3448	0.744732	Oropharyngeal cancer || id:ieu-b-97	Oropharyngeal cancer	Oropharyngeal cancer || ||
127	rs11076175	G	A	G	A	0.0229033	0.046	0.175666	0.174	FALSE	FALSE	FALSE	ieu-b-97	16	57006378	0.071	3448	0.515435	Oropharyngeal cancer || id:ieu-b-97	Oropharyngeal cancer	Oropharyngeal cancer || ||
128	rs11207969	G	A	G	A	0.0209396	0.008	0.351365	0.316	FALSE	FALSE	FALSE	ieu-b-97	1	62911751	0.059	3448	0.886956	Oropharyngeal cancer || id:ieu-b-97	Oropharyngeal cancer	Oropharyngeal cancer || ||
129	rs11264361	G	T	G	T	0.0174875	0.101	0.251408	0.241	FALSE	FALSE	FALSE	ieu-b-97	1	155289545	0.063	3448	0.113264	Oropharyngeal cancer || id:ieu-b-97	Oropharyngeal cancer	Oropharyngeal cancer || ||
130	rs1128535	T	C	T	C	0.0164131	-0.006	0.500049	0.483	FALSE	FALSE	FALSE	ieu-b-97	3	49866392	0.055	3448	0.91988	Oropharyngeal cancer || id:ieu-b-97	Oropharyngeal cancer	Oropharyngeal cancer || ||
131	rs115288876	A	G	A	G	0.0788065	0.024	0.0433211	0.036	FALSE	FALSE	FALSE	ieu-b-97	1	152000117	0.149	3448	0.874295	Oropharyngeal cancer || id:ieu-b-97	Oropharyngeal cancer	Oropharyngeal cancer || ||
132	rs11542462	A	G	A	G	-0.0247803	0.031	0.133511	0.123	FALSE	FALSE	FALSE	ieu-b-97	16	82033810	0.082	3448	0.7026	Oropharyngeal cancer || id:ieu-b-97	Oropharyngeal cancer	Oropharyngeal cancer || ||
133	rs11726886	A	C	A	C	-0.0536726	0.019	0.290783	0.287	FALSE	FALSE	FALSE	ieu-b-97	4	72822599	0.059	3448	0.748201	Oropharyngeal cancer || id:ieu-b-97	Oropharyngeal cancer	Oropharyngeal cancer || ||
134	rs11791258	A	G	A	G	0.0140808	-0.034	0.191072	0.19	FALSE	FALSE	FALSE	ieu-b-97	9	107632644	0.077	3448	0.664248	Oropharyngeal cancer || id:ieu-b-97	Oropharyngeal cancer	Oropharyngeal cancer || ||
135	rs11867297	T	C	T	C	0.0135432	0.1	0.38533	0.395	FALSE	FALSE	FALSE	ieu-b-97	17	66433493	0.055	3448	0.0726691	Oropharyngeal cancer || id:ieu-b-97	Oropharyngeal cancer	Oropharyngeal cancer || ||
136	rs12056768	G	T	G	T	-0.0231961	0.061	0.584044	0.604	FALSE	FALSE	FALSE	ieu-b-97	8	116988527	0.055	3448	0.268696	Oropharyngeal cancer || id:ieu-b-97	Oropharyngeal cancer	Oropharyngeal cancer || ||

137	rs12153819	T	C	T	C	-0.0178178	0.05	0.123154	0.118	FALSE	FALSE	FALSE	ieu-b-97	6	83773049	0.083		3448	0.550606	Oropharyngeal cancer || id:ieu-b-97	Oropharyngeal cancer	Oropharyngeal cancer || ||
138	rs12283049	G	A	G	A	-0.0564566	-0.078	0.234433	0.244	FALSE	FALSE	FALSE	ieu-b-97	11	14690192	0.063		3448	0.2134	Oropharyngeal cancer || id:ieu-b-97	Oropharyngeal cancer	Oropharyngeal cancer || ||
139	rs12324720	A	G	A	G	-0.0149159	-0.056	0.174629	0.191	FALSE	FALSE	FALSE	ieu-b-97	15	64092140	0.072		3448	0.435773	Oropharyngeal cancer || id:ieu-b-97	Oropharyngeal cancer	Oropharyngeal cancer || ||
140	rs12462826	A	G	A	G	-0.0132119	-0.044	0.369566	0.373	FALSE	FALSE	FALSE	ieu-b-97	19	11957001	0.056		3448	0.430878	Oropharyngeal cancer || id:ieu-b-97	Oropharyngeal cancer	Oropharyngeal cancer || ||
141	rs12501515	A	G	A	G	-0.078957	-0.042	0.58966	0.563	FALSE	FALSE	FALSE	ieu-b-97	4	72592838	0.055		3448	0.44558	Oropharyngeal cancer || id:ieu-b-97	Oropharyngeal cancer	Oropharyngeal cancer || ||
142	rs1260326	C	T	C	T	0.0197194	0.059	0.603925	0.571	FALSE	FALSE	FALSE	ieu-b-97	2	27730940	0.056		3448	0.295972	Oropharyngeal cancer || id:ieu-b-97	Oropharyngeal cancer	Oropharyngeal cancer || ||
143	rs12775091	T	C	T	C	0.0155618	0.086	0.213441	0.203	FALSE	FALSE	FALSE	ieu-b-97	10	91543551	0.068		3448	0.205049	Oropharyngeal cancer || id:ieu-b-97	Oropharyngeal cancer	Oropharyngeal cancer || ||
144	rs13076508	C	T	C	T	0.0250544	0.16	0.0535034	0.053	FALSE	FALSE	FALSE	ieu-b-97	3	52407805	0.118		3448	0.177835	Oropharyngeal cancer || id:ieu-b-97	Oropharyngeal cancer	Oropharyngeal cancer || ||
145	rs13108245	G	A	G	A	-0.0122246	-0.119	0.386601	0.404	FALSE	FALSE	FALSE	ieu-b-97	4	57790205	0.056		3448	0.034231	Oropharyngeal cancer || id:ieu-b-97	Oropharyngeal cancer	Oropharyngeal cancer || ||
146	rs1321247	T	A	T	A	-0.0221847	-0.051	0.101578	0.081	FALSE	TRUE	FALSE	ieu-b-97	6	25662873	0.096		3448	0.596804	Oropharyngeal cancer || id:ieu-b-97	Oropharyngeal cancer	Oropharyngeal cancer || ||
147	rs13294734	T	C	T	C	0.012568	0.014	0.466128	0.454	FALSE	FALSE	FALSE	ieu-b-97	9	80710910	0.055		3448	0.799551	Oropharyngeal cancer || id:ieu-b-97	Oropharyngeal cancer	Oropharyngeal cancer || ||
148	rs1343776	A	G	A	G	0.0180762	0.137	0.22133	0.221	FALSE	FALSE	FALSE	ieu-b-97	1	41757718	0.065		3448	0.0364141	Oropharyngeal cancer || id:ieu-b-97	Oropharyngeal cancer	Oropharyngeal cancer || ||
149	rs138335	G	C	G	C	-0.0137663	-0.026	0.658587	0.656	FALSE	TRUE	FALSE	ieu-b-97	22	41227086	0.059		3448	0.648393	Oropharyngeal cancer || id:ieu-b-97	Oropharyngeal cancer	Oropharyngeal cancer || ||
150	rs1384687	A	G	A	G	-0.0168656	0.03	0.132271	0.144	FALSE	FALSE	FALSE	ieu-b-97	8	61525963	0.079		3448	0.712397	Oropharyngeal cancer || id:ieu-b-97	Oropharyngeal cancer	Oropharyngeal cancer || ||
151	rs142004400	C	A	C	A	-0.0310034	-0.293	0.0342066	0.035	FALSE	FALSE	FALSE	ieu-b-97	14	50829560	0.164		3448	0.0742113	Oropharyngeal cancer || id:ieu-b-97	Oropharyngeal cancer	Oropharyngeal cancer || ||
152	rs142158911	A	G	A	G	0.026284	-0.046	0.111826	0.126	FALSE	FALSE	FALSE	ieu-b-97	19	11190534	0.086		3448	0.597083	Oropharyngeal cancer || id:ieu-b-97	Oropharyngeal cancer	Oropharyngeal cancer || ||
153	rs144965707	A	G	A	G	-0.0348143	0.181	0.0618296	0.071	FALSE	FALSE	FALSE	ieu-b-97	11	14059511	0.113		3448	0.106717	Oropharyngeal cancer || id:ieu-b-97	Oropharyngeal cancer	Oropharyngeal cancer || ||
154	rs1627043	C	G	C	G	-0.0486441	0.393	0.0332414	0.032	FALSE	TRUE	FALSE	ieu-b-97	11	71110175	0.153		3448	0.0102103	Oropharyngeal cancer || id:ieu-b-97	Oropharyngeal cancer	Oropharyngeal cancer || ||
155	rs1684600	T	C	T	C	-0.0125301	-0.078	0.298695	0.315	FALSE	FALSE	FALSE	ieu-b-97	16	4594671	0.061		3448	0.202045	Oropharyngeal cancer || id:ieu-b-97	Oropharyngeal cancer	Oropharyngeal cancer || ||
156	rs17207784	C	T	C	T	-0.0134939	0.096	0.324195	0.339	FALSE	FALSE	FALSE	ieu-b-97	6	22768668	0.058		3448	0.0965806	Oropharyngeal cancer || id:ieu-b-97	Oropharyngeal cancer	Oropharyngeal cancer || ||
157	rs17473257	A	G	A	G	-0.0611372	0.028	0.0172466	0.019	FALSE	FALSE	FALSE	ieu-b-97	11	14283186	0.221		3448	0.89914	Oropharyngeal cancer || id:ieu-b-97	Oropharyngeal cancer	Oropharyngeal cancer || ||
158	rs1800588	T	C	T	C	-0.0305021	0.024	0.214964	0.21	FALSE	FALSE	FALSE	ieu-b-97	15	58723939	0.066		3446	0.718701	Oropharyngeal cancer || id:ieu-b-97	Oropharyngeal cancer	Oropharyngeal cancer || ||
159	rs1841850	C	A	C	A	0.0304395	0.16	0.117085	0.114	FALSE	FALSE	FALSE	ieu-b-97	20	52718555	0.088		3448	0.0682952	Oropharyngeal cancer || id:ieu-b-97	Oropharyngeal cancer	Oropharyngeal cancer || ||
160	rs1858889	C	A	C	A	0.0134514	0.052	0.502532	0.503	FALSE	FALSE	FALSE	ieu-b-97	7	107117447	0.054		3448	0.345416	Oropharyngeal cancer || id:ieu-b-97	Oropharyngeal cancer	Oropharyngeal cancer || ||
161	rs1871395	G	A	G	A	-0.0203733	-0.064	0.152679	0.164	FALSE	FALSE	FALSE	ieu-b-97	12	21352315	0.075		3448	0.3968	Oropharyngeal cancer || id:ieu-b-97	Oropharyngeal cancer	Oropharyngeal cancer || ||
162	rs1949633	C	T	C	T	0.0114162	0.069	0.605759	0.591	FALSE	FALSE	FALSE	ieu-b-97	3	153758806	0.056		3448	0.222893	Oropharyngeal cancer || id:ieu-b-97	Oropharyngeal cancer	Oropharyngeal cancer || ||
163	rs2037511	A	G	A	G	0.0176624	0.022	0.165924	0.18	FALSE	FALSE	FALSE	ieu-b-97	18	61366207	0.072		3446	0.7583	Oropharyngeal cancer || id:ieu-b-97	Oropharyngeal cancer	Oropharyngeal cancer || ||
164	rs2074735	C	G	C	G	0.0292667	0.228	0.0648365	0.067	FALSE	TRUE	FALSE	ieu-b-97	22	31535872	0.106		3448	0.03125	Oropharyngeal cancer || id:ieu-b-97	Oropharyngeal cancer	Oropharyngeal cancer || ||
165	rs2171427	A	G	A	G	-0.0165489	-0.231	0.156491	0.175	FALSE	FALSE	FALSE	ieu-b-97	12	24822366	0.077		3448	0.00266594	Oropharyngeal cancer || id:ieu-b-97	Oropharyngeal cancer	Oropharyngeal cancer || ||
166	rs2229742	C	G	C	G	-0.0249837	-0.024	0.104659	0.102	FALSE	TRUE	FALSE	ieu-b-97	21	16339172	0.092		3448	0.796706	Oropharyngeal cancer || id:ieu-b-97	Oropharyngeal cancer	Oropharyngeal cancer || ||
167	rs2245133	C	T	C	T	-0.0212917	-0.117	0.164339	0.157	FALSE	FALSE	FALSE	ieu-b-97	6	131931092	0.077		3448	0.127448	Oropharyngeal cancer || id:ieu-b-97	Oropharyngeal cancer	Oropharyngeal cancer || ||
168	rs2297991	C	T	C	T	0.0127547	0.094	0.718478	0.682	FALSE	FALSE	FALSE	ieu-b-97	10	113913222	0.06		3448	0.118654	Oropharyngeal cancer || id:ieu-b-97	Oropharyngeal cancer	Oropharyngeal cancer || ||
169	rs2398113	G	A	G	A	-0.0117606	0.035	0.423526	0.397	FALSE	FALSE	FALSE	ieu-b-97	10	10078742	0.061		3448	0.560761	Oropharyngeal cancer || id:ieu-b-97	Oropharyngeal cancer	Oropharyngeal cancer || ||
170	rs2494429	G	A	G	A	-0.0148459	0.033	0.822999	0.81	FALSE	FALSE	FALSE	ieu-b-97	1	2339395	0.075		3448	0.658105	Oropharyngeal cancer || id:ieu-b-97	Oropharyngeal cancer	Oropharyngeal cancer || ||
171	rs2511279	G	C	G	C	0.0981721	0.025	0.960373	0.943	FALSE	TRUE	FALSE	ieu-b-97	11	71130419	0.132		3448	0.852467	Oropharyngeal cancer || id:ieu-b-97	Oropharyngeal cancer	Oropharyngeal cancer || ||
172	rs2595644	T	G	T	G	-0.0122625	-0.069	0.384889	0.403	FALSE	FALSE	FALSE	ieu-b-97	7	43980540	0.056		3448	0.215096	Oropharyngeal cancer || id:ieu-b-97	Oropharyngeal cancer	Oropharyngeal cancer || ||
173	rs2710651	A	G	A	G	-0.0115892	-0.007	0.526101	0.502	FALSE	FALSE	FALSE	ieu-b-97	2	63166379	0.06		3448	0.9131	Oropharyngeal cancer || id:ieu-b-97	Oropharyngeal cancer	Oropharyngeal cancer || ||
174	rs2756119	A	G	A	G	0.0121434	-0.026	0.38143	0.377	FALSE	FALSE	FALSE	ieu-b-97	14	104001517	0.057		3448	0.644562	Oropharyngeal cancer || id:ieu-b-97	Oropharyngeal cancer	Oropharyngeal cancer || ||
175	rs2807834	G	T	G	T	-0.0150625	-0.076	0.68514	0.685	FALSE	FALSE	FALSE	ieu-b-97	1	220970593	0.059		3448	0.194988	Oropharyngeal cancer || id:ieu-b-97	Oropharyngeal cancer	Oropharyngeal cancer || ||
176	rs28435470	A	G	A	G	-0.0118696	-0.029	0.663204	0.687	FALSE	FALSE	FALSE	ieu-b-97	12	133075523	0.059		3448	0.625235	Oropharyngeal cancer || id:ieu-b-97	Oropharyngeal cancer	Oropharyngeal cancer || ||
177	rs2847500	A	G	A	G	-0.022548	0.017	0.123204	0.124	FALSE	FALSE	FALSE	ieu-b-97	11	120114421	0.089		3448	0.85212	Oropharyngeal cancer || id:ieu-b-97	Oropharyngeal cancer	Oropharyngeal cancer || ||
178	rs290400	A	G	A	G	-0.0130967	-0.071	0.665472	0.674	FALSE	FALSE	FALSE	ieu-b-97	20	52698179	0.058		3448	0.214862	Oropharyngeal cancer || id:ieu-b-97	Oropharyngeal cancer	Oropharyngeal cancer || ||
179	rs3114045	C	T	C	T	-0.0221737	-0.041	0.866137	0.849	FALSE	FALSE	FALSE	ieu-b-97	4	100252560	0.081		3448	0.616456	Oropharyngeal cancer || id:ieu-b-97	Oropharyngeal cancer	Oropharyngeal cancer || ||
180	rs325393	T	G	T	G	-0.0136497	-0.02	0.278218	0.27	FALSE	FALSE	FALSE	ieu-b-97	15	100229260	0.062		3448	0.746323	Oropharyngeal cancer || id:ieu-b-97	Oropharyngeal cancer	Oropharyngeal cancer || ||
181	rs34186890	G	A	G	A	-0.0156853	-0.077	0.259637	0.254	FALSE	FALSE	FALSE	ieu-b-97	3	141720712	0.064		3448	0.232016	Oropharyngeal cancer || id:ieu-b-97	Oropharyngeal cancer	Oropharyngeal cancer || ||
182	rs34726834	T	C	T	C	0.014013	0.037	0.252209	0.253	FALSE	FALSE	FALSE	ieu-b-97	8	25889606	0.062		3448	0.547017	Oropharyngeal cancer || id:ieu-b-97	Oropharyngeal cancer	Oropharyngeal cancer || ||
183	rs35270497	T	C	T	C	0.0156723	-0.119	0.176236	0.171	FALSE	FALSE	FALSE	ieu-b-97	2	38259872	0.074		3448	0.109174	Oropharyngeal cancer || id:ieu-b-97	Oropharyngeal cancer	Oropharyngeal cancer || ||
184	rs35823191	C	T	C	T	-0.0232636	-0.037	0.342049	0.356	FALSE	FALSE	FALSE	ieu-b-97	1	17560123	0.058		3448	0.521314	Oropharyngeal cancer || id:ieu-b-97	Oropharyngeal cancer	Oropharyngeal cancer || ||
185	rs3732220	A	G	A	G	-0.0478406	-0.105	0.0852646	0.087	FALSE	FALSE	FALSE	ieu-b-97	2	234627048	0.099		3448	0.287377	Oropharyngeal cancer || id:ieu-b-97	Oropharyngeal cancer	Oropharyngeal cancer || ||
186	rs3829251	A	G	A	G	-0.114453	0.046	0.133277	0.152	FALSE	FALSE	FALSE	ieu-b-97	11	71194559	0.075		3448	0.5403	Oropharyngeal cancer || id:ieu-b-97	Oropharyngeal cancer	Oropharyngeal cancer || ||
187	rs4147536	C	A	C	A	-0.0148038	-0.108	0.788613	0.797	FALSE	FALSE	FALSE	ieu-b-97	4	100239112	0.068		3448	0.110085	Oropharyngeal cancer || id:ieu-b-97	Oropharyngeal cancer	Oropharyngeal cancer || ||
188	rs4348160	G	T	G	T	-0.0258401	0.105	0.326968	0.338	FALSE	FALSE	FALSE	ieu-b-97	4	70017531	0.058		3448	0.0717431	Oropharyngeal cancer || id:ieu-b-97	Oropharyngeal cancer	Oropharyngeal cancer || ||
189	rs4364259	A	G	A	G	0.0172408	0.028	0.198665	0.198	FALSE	FALSE	FALSE	ieu-b-97	4	15892159	0.072		3448	0.70275	Oropharyngeal cancer || id:ieu-b-97	Oropharyngeal cancer	Oropharyngeal cancer || ||
190	rs4420638	G	A	G	A	-0.0192973	-0.028	0.176831	0.171	FALSE	FALSE	FALSE	ieu-b-97	19	45422946	0.074		3430	0.6939	Oropharyngeal cancer || id:ieu-b-97	Oropharyngeal cancer	Oropharyngeal cancer || ||
191	rs4580037	C	A	C	A	-0.0135627	-0.084	0.285578	0.29	FALSE	FALSE	FALSE	ieu-b-97	13	55702646	0.061		3448	0.166016	Oropharyngeal cancer || id:ieu-b-97	Oropharyngeal cancer	Oropharyngeal cancer || ||
192	rs512083	C	T	C	T	0.0122172	0.056	0.462488	0.476	FALSE	FALSE	FALSE	ieu-b-97	1	46027355	0.055		3448	0.304928	Oropharyngeal cancer || id:ieu-b-97	Oropharyngeal cancer	Oropharyngeal cancer || ||
193	rs57601828	T	A	T	A	0.011542	-0.001	0.394508	0.377	FALSE	TRUE	FALSE	ieu-b-97	12	93250469	0.057		3448	0.9891	Oropharyngeal cancer || id:ieu-b-97	Oropharyngeal cancer	Oropharyngeal cancer || ||
194	rs5770794	T	C	T	C	-0.0133141	0	0.3143	0.314	FALSE	FALSE	FALSE	ieu-b-97	22	50880781	0.059		3448	0.997107	Oropharyngeal cancer || id:ieu-b-97	Oropharyngeal cancer	Oropharyngeal cancer || ||
195	rs6129648	G	A	G	A	0.0140631	-0.019	0.379837	0.357	FALSE	FALSE	FALSE	ieu-b-97	20	39231118	0.057		3448	0.728939	Oropharyngeal cancer || id:ieu-b-97	Oropharyngeal cancer	Oropharyngeal cancer || ||
196	rs61698755	C	T	C	T	-0.011465	-0.07	0.560041	0.548	FALSE	FALSE	FALSE	ieu-b-97	17	79257880	0.056		3448	0.205177	Oropharyngeal cancer || id:ieu-b-97	Oropharyngeal cancer	Oropharyngeal cancer || ||
197	rs61813875	G	C	G	C	0.0821291	-0.212	0.0248209	0.024	FALSE	TRUE	FALSE	ieu-b-97	1	152536650	0.196		3448	0.280008	Oropharyngeal cancer || id:ieu-b-97	Oropharyngeal cancer	Oropharyngeal cancer || ||
198	rs61887421	C	T	C	T	-0.036726	-0.129	0.0300871	0.032	FALSE	FALSE	FALSE	ieu-b-97	11	70949673	0.168		3448	0.445044	Oropharyngeal cancer || id:ieu-b-97	Oropharyngeal cancer	Oropharyngeal cancer || ||
199	rs62007299	A	G	A	G	-0.0124205	-0.063	0.712958	0.707	FALSE	FALSE	FALSE	ieu-b-97	15	77711719	0.06		3448	0.293997	Oropharyngeal cancer || id:ieu-b-97	Oropharyngeal cancer	Oropharyngeal cancer || ||
200	rs62129966	A	C	A	C	0.0611636	-0.09	0.160854	0.174	FALSE	FALSE	FALSE	ieu-b-97	19	48374950	0.075		3448	0.227975	Oropharyngeal cancer || id:ieu-b-97	Oropharyngeal cancer	Oropharyngeal cancer || ||
201	rs635634	T	C	T	C	-0.0150476	0.161	0.186573	0.19	FALSE	FALSE	FALSE	ieu-b-97	9	136155000	0.068		3448	0.0172723	Oropharyngeal cancer || id:ieu-b-97	Oropharyngeal cancer	Oropharyngeal cancer || ||
202	rs6438900	G	C	G	C	0.0150486	-0.067	0.256034	0.271	FALSE	TRUE	FALSE	ieu-b-97	3	125148287	0.062		3448	0.278911	Oropharyngeal cancer || id:ieu-b-97	Oropharyngeal cancer	Oropharyngeal cancer || ||
203	rs6672758	T	C	T	C	0.0162478	0.01	0.80023	0.795	FALSE	FALSE	FALSE	ieu-b-97	1	230303512	0.067		3448	0.887259	Oropharyngeal cancer || id:ieu-b-97	Oropharyngeal cancer	Oropharyngeal cancer || ||
204	rs6834488	T	C	T	C	-0.01445	0.047	0.422833	0.414	FALSE	FALSE	FALSE	ieu-b-97	4	88178919	0.057		3448	0.409845	Oropharyngeal cancer || id:ieu-b-97	Oropharyngeal cancer	Oropharyngeal cancer || ||
205	rs71599974	G	A	G	A	0.0257378	-0.022	0.148048	0.134	FALSE	FALSE	FALSE	ieu-b-97	4	71765339	0.088		3448	0.801213	Oropharyngeal cancer || id:ieu-b-97	Oropharyngeal cancer	Oropharyngeal cancer || ||
206	rs727857	A	G	A	G	-0.0120548	0.011	0.61181	0.606	FALSE	FALSE	FALSE	ieu-b-97	2	58981967	0.056		3448	0.840985	Oropharyngeal cancer || id:ieu-b-97	Oropharyngeal cancer	Oropharyngeal cancer || ||
207	rs733454	T	C	T	C	0.0188545	0.16	0.0991663	0.079	FALSE	FALSE	FALSE	ieu-b-97	11	76477721	0.096		3448	0.0962166	Oropharyngeal cancer || id:ieu-b-97	Oropharyngeal cancer	Oropharyngeal cancer || ||
208	rs73413596	C	T	C	T	0.0223468	-0.097	0.0740317	0.081	FALSE	FALSE	FALSE	ieu-b-97	12	111582630	0.101		3448	0.339202	Oropharyngeal cancer || id:ieu-b-97	Oropharyngeal cancer	Oropharyngeal cancer || ||
209	rs742493	C	T	C	T	0.0183528	0.048	0.112871	0.126	FALSE	FALSE	FALSE	ieu-b-97	6	40998167	0.082		3448	0.555437	Oropharyngeal cancer || id:ieu-b-97	Oropharyngeal cancer	Oropharyngeal cancer || ||
210	rs7528419	G	A	G	A	0.0215389	0.102	0.22441	0.21	FALSE	FALSE	FALSE	ieu-b-97	1	109817192	0.066		3435	0.1204	Oropharyngeal cancer || id:ieu-b-97	Oropharyngeal cancer	Oropharyngeal cancer || ||
211	rs7569755	A	G	A	G	0.0136395	-0.066	0.288618	0.286	FALSE	FALSE	FALSE	ieu-b-97	2	118648261	0.061		3448	0.282838	Oropharyngeal cancer || id:ieu-b-97	Oropharyngeal cancer	Oropharyngeal cancer || ||
212	rs7580771	T	G	T	G	-0.0165625	0.01	0.175983	0.198	FALSE	FALSE	FALSE	ieu-b-97	2	101428119	0.069		3448	0.886635	Oropharyngeal cancer || id:ieu-b-97	Oropharyngeal cancer	Oropharyngeal cancer || ||
213	rs7652808	G	T	G	T	-0.0212858	0.043	0.649492	0.611	FALSE	FALSE	FALSE	ieu-b-97	3	85604158	0.056		3448	0.444385	Oropharyngeal cancer || id:ieu-b-97	Oropharyngeal cancer	Oropharyngeal cancer || ||
214	rs7712001	G	T	G	T	0.011939	-0.123	0.440176	0.442	FALSE	FALSE	FALSE	ieu-b-97	5	148020950	0.057		3448	0.0315159	Oropharyngeal cancer || id:ieu-b-97	Oropharyngeal cancer	Oropharyngeal cancer || ||
215	rs7784802	T	A	T	A	0.0133202	-0.043	0.358913	0.29	FALSE	TRUE	FALSE	ieu-b-97	7	64020652	0.06		3448	0.476512	Oropharyngeal cancer || id:ieu-b-97	Oropharyngeal cancer	Oropharyngeal cancer || ||
216	rs77924615	A	G	A	G	-0.0152475	0.03	0.194374	0.198	FALSE	FALSE	FALSE	ieu-b-97	16	20392332	0.072		3448	0.684244	Oropharyngeal cancer || id:ieu-b-97	Oropharyngeal cancer	Oropharyngeal cancer || ||
217	rs77960347	G	A	G	A	-0.0525688	-0.36	0.0127013	0.015	FALSE	FALSE	FALSE	ieu-b-97	18	47109955	0.272		3448	0.186489	Oropharyngeal cancer || id:ieu-b-97	Oropharyngeal cancer	Oropharyngeal cancer || ||
218	rs78649910	A	T	A	T	-0.0191377	0.092	0.105728	0.111	FALSE	TRUE	FALSE	ieu-b-97	4	3482213	0.086		3448	0.284883	Oropharyngeal cancer || id:ieu-b-97	Oropharyngeal cancer	Oropharyngeal cancer || ||
219	rs7955128	T	A	T	A	0.0130617	0.017	0.52053	0.516	FALSE	TRUE	TRUE	ieu-b-97	12	38684121	0.056		3448	0.766595	Oropharyngeal cancer || id:ieu-b-97	Oropharyngeal cancer	Oropharyngeal cancer || ||
220	rs8018720	C	G	C	G	-0.0344962	-0.088	0.823508	0.831	FALSE	TRUE	FALSE	ieu-b-97	14	39556185	0.072		3448	0.222623	Oropharyngeal cancer || id:ieu-b-97	Oropharyngeal cancer	Oropharyngeal cancer || ||
221	rs804281	G	A	G	A	0.015895	-0.009	0.583498	0.573	FALSE	FALSE	FALSE	ieu-b-97	8	11611865	0.056		3448	0.867756	Oropharyngeal cancer || id:ieu-b-97	Oropharyngeal cancer	Oropharyngeal cancer || ||
222	rs8107974	T	A	T	A	0.0355672	0.001	0.0763013	0.073	FALSE	TRUE	FALSE	ieu-b-97	19	19388500	0.106		3448	0.992211	Oropharyngeal cancer || id:ieu-b-97	Oropharyngeal cancer	Oropharyngeal cancer || ||
223	rs8121940	G	C	G	C	-0.0435612	-0.023	0.19762	0.208	FALSE	TRUE	FALSE	ieu-b-97	20	52742306	0.076		3448	0.762491	Oropharyngeal cancer || id:ieu-b-97	Oropharyngeal cancer	Oropharyngeal cancer || ||
224	rs9375037	C	A	C	A	0.0117059	0.03	0.443168	0.429	FALSE	FALSE	FALSE	ieu-b-97	6	121856794	0.054		3448	0.587677	Oropharyngeal cancer || id:ieu-b-97	Oropharyngeal cancer	Oropharyngeal cancer || ||
225	rs9409266	A	G	A	G	-0.0167771	0.072	0.862222	0.863	FALSE	FALSE	FALSE	ieu-b-97	9	125824055	0.081		3448	0.372048	Oropharyngeal cancer || id:ieu-b-97	Oropharyngeal cancer	Oropharyngeal cancer || ||
226	rs964184	C	G	C	G	0.0406845	0.021	0.867234	0.857	FALSE	TRUE	FALSE	ieu-b-97	11	116648917	0.079		3448	0.7838	Oropharyngeal cancer || id:ieu-b-97	Oropharyngeal cancer	Oropharyngeal cancer || ||
227	rs9847248	A	G	A	G	-0.0123077	-0.045	0.712969	0.698	FALSE	FALSE	FALSE	ieu-b-97	3	18804655	0.059		3448	0.44999	Oropharyngeal cancer || id:ieu-b-97	Oropharyngeal cancer	Oropharyngeal cancer || ||
228	rs986649	G	A	G	A	0.0128637	-0.027	0.321628	0.328	FALSE	FALSE	FALSE	ieu-b-97	5	118668050	0.058		3448	0.6304	Oropharyngeal cancer || id:ieu-b-97	Oropharyngeal cancer	Oropharyngeal cancer || ||
229	rs9946771	T	C	T	C	-0.0233992	0.189	0.0663426	0.066	FALSE	FALSE	FALSE	ieu-b-97	18	28918628	0.105		3448	0.072129	Oropharyngeal cancer || id:ieu-b-97	Oropharyngeal cancer	Oropharyngeal cancer || ||
230	rs116970203	G	A	G	A	0.372	-0.07	0.98	0.979	FALSE	FALSE	FALSE	ieu-b-97	11	14876718	0.184		3448	0.704333	Oropharyngeal cancer || id:ieu-b-97	Oropharyngeal cancer	Oropharyngeal cancer || ||
231	rs13084927	C	A	C	A	0.055	-0.047	0.83	0.824	FALSE	FALSE	FALSE	ieu-b-97	3	72709792	0.073		3448	0.523026	Oropharyngeal cancer || id:ieu-b-97	Oropharyngeal cancer	Oropharyngeal cancer || ||
232	rs17216707	T	C	T	C	0.074	0.033	0.8	0.795	FALSE	FALSE	FALSE	ieu-b-97	20	52732362	0.077		3448	0.67326	Oropharyngeal cancer || id:ieu-b-97	Oropharyngeal cancer	Oropharyngeal cancer || ||
233	rs28364617	G	T	G	T	0.127	-0.12	0.71	0.743	FALSE	FALSE	FALSE	ieu-b-97	11	71159764	0.063		3448	0.0565913	Oropharyngeal cancer || id:ieu-b-97	Oropharyngeal cancer	Oropharyngeal cancer || ||
234	rs3819817	C	T	C	T	0.058	0.005	0.45	0.454	FALSE	FALSE	FALSE	ieu-b-97	12	96378771	0.056		3448	0.933661	Oropharyngeal cancer || id:ieu-b-97	Oropharyngeal cancer	Oropharyngeal cancer || ||
235	rs9304669	T	C	T	C	0.054	-0.113	0.16	0.18	FALSE	FALSE	FALSE	ieu-b-97	19	48384385	0.074		3448	0.128245	Oropharyngeal cancer || id:ieu-b-97	Oropharyngeal cancer	Oropharyngeal cancer || ||
236	rs11172066	T	A	T	A	0.166	-0.024	0.14	0.145	FALSE	TRUE	FALSE	ieu-b-97	12	57319491	0.081		3448	0.765366	Oropharyngeal cancer || id:ieu-b-97	Oropharyngeal cancer	Oropharyngeal cancer || ||
237	rs28364617	G	T	G	T	0.131	-0.12	0.72	0.743	FALSE	FALSE	FALSE	ieu-b-97	11	71159764	0.063		3448	0.0565913	Oropharyngeal cancer || id:ieu-b-97	Oropharyngeal cancer	Oropharyngeal cancer || ||
238	rs10277163	G	A	G	A	-0.0143336	0.02	0.254715	0.224	FALSE	FALSE	FALSE	ieu-b-98	7	21569089	0.137		932	0.886623	Oropharyngeal cancer || id:ieu-b-98	Oropharyngeal cancer	Oropharyngeal cancer || ||
239	rs1038165	T	C	T	C	0.0115149	0.06	0.579451	0.498	FALSE	FALSE	FALSE	ieu-b-98	12	68665940	0.119		932	0.617297	Oropharyngeal cancer || id:ieu-b-98	Oropharyngeal cancer	Oropharyngeal cancer || ||
240	rs1042034	T	C	T	C	-0.0151254	-0.103	0.792138	0.793	FALSE	FALSE	FALSE	ieu-b-98	2	21226560	0.141		932	0.467881	Oropharyngeal cancer || id:ieu-b-98	Oropharyngeal cancer	Oropharyngeal cancer || ||
241	rs10438978	C	T	C	T	-0.0172243	-0.245	0.820277	0.822	FALSE	FALSE	FALSE	ieu-b-98	18	47158186	0.151		932	0.106231	Oropharyngeal cancer || id:ieu-b-98	Oropharyngeal cancer	Oropharyngeal cancer || ||
242	rs1047891	A	C	A	C	-0.0133984	0.034	0.317078	0.302	FALSE	FALSE	FALSE	ieu-b-98	2	211540507	0.122		932	0.776	Oropharyngeal cancer || id:ieu-b-98	Oropharyngeal cancer	Oropharyngeal cancer || ||
243	rs1048328	A	G	A	G	0.0313497	0.135	0.0797967	0.08	FALSE	FALSE	FALSE	ieu-b-98	19	51527364	0.209		932	0.518601	Oropharyngeal cancer || id:ieu-b-98	Oropharyngeal cancer	Oropharyngeal cancer || ||
244	rs10859995	C	T	C	T	-0.0436264	0.015	0.579841	0.493	FALSE	FALSE	FALSE	ieu-b-98	12	96378771	0.122		932	0.902233	Oropharyngeal cancer || id:ieu-b-98	Oropharyngeal cancer	Oropharyngeal cancer || ||
245	rs11023159	C	T	C	T	0.0482117	0.18	0.0325143	0.022	FALSE	FALSE	FALSE	ieu-b-98	11	14262063	0.392		932	0.646766	Oropharyngeal cancer || id:ieu-b-98	Oropharyngeal cancer	Oropharyngeal cancer || ||
246	rs11076175	G	A	G	A	0.0229033	-0.031	0.175666	0.191	FALSE	FALSE	FALSE	ieu-b-98	16	57006378	0.154		932	0.837022	Oropharyngeal cancer || id:ieu-b-98	Oropharyngeal cancer	Oropharyngeal cancer || ||
247	rs11207969	G	A	G	A	0.0209396	-0.135	0.351365	0.328	FALSE	FALSE	FALSE	ieu-b-98	1	62911751	0.127		932	0.288596	Oropharyngeal cancer || id:ieu-b-98	Oropharyngeal cancer	Oropharyngeal cancer || ||
248	rs11264361	G	T	G	T	0.0174875	0.263	0.251408	0.256	FALSE	FALSE	FALSE	ieu-b-98	1	155289545	0.136		932	0.0521591	Oropharyngeal cancer || id:ieu-b-98	Oropharyngeal cancer	Oropharyngeal cancer || ||
249	rs1128535	T	C	T	C	0.0164131	-0.081	0.500049	0.471	FALSE	FALSE	FALSE	ieu-b-98	3	49866392	0.12		932	0.498	Oropharyngeal cancer || id:ieu-b-98	Oropharyngeal cancer	Oropharyngeal cancer || ||
250	rs115288876	A	G	A	G	0.0788065	0.812	0.0433211	0.014	FALSE	FALSE	FALSE	ieu-b-98	1	152000117	0.535		932	0.129318	Oropharyngeal cancer || id:ieu-b-98	Oropharyngeal cancer	Oropharyngeal cancer || ||
251	rs11542462	A	G	A	G	-0.0247803	0.025	0.133511	0.124	FALSE	FALSE	FALSE	ieu-b-98	16	82033810	0.173		932	0.8849	Oropharyngeal cancer || id:ieu-b-98	Oropharyngeal cancer	Oropharyngeal cancer || ||
252	rs11726886	A	C	A	C	-0.0536726	0.075	0.290783	0.227	FALSE	FALSE	FALSE	ieu-b-98	4	72822599	0.14		932	0.5897	Oropharyngeal cancer || id:ieu-b-98	Oropharyngeal cancer	Oropharyngeal cancer || ||
253	rs11791258	A	G	A	G	0.0140808	0.12	0.191072	0.185	FALSE	FALSE	FALSE	ieu-b-98	9	107632644	0.168		932	0.47295	Oropharyngeal cancer || id:ieu-b-98	Oropharyngeal cancer	Oropharyngeal cancer || ||
254	rs11867297	T	C	T	C	0.0135432	0.142	0.38533	0.411	FALSE	FALSE	FALSE	ieu-b-98	17	66433493	0.121		932	0.239528	Oropharyngeal cancer || id:ieu-b-98	Oropharyngeal cancer	Oropharyngeal cancer || ||
255	rs12056768	G	T	G	T	-0.0231961	0.031	0.584044	0.638	FALSE	FALSE	FALSE	ieu-b-98	8	116988527	0.123		932	0.802084	Oropharyngeal cancer || id:ieu-b-98	Oropharyngeal cancer	Oropharyngeal cancer || ||
256	rs12153819	T	C	T	C	-0.0178178	-0.121	0.123154	0.091	FALSE	FALSE	FALSE	ieu-b-98	6	83773049	0.212		932	0.56674	Oropharyngeal cancer || id:ieu-b-98	Oropharyngeal cancer	Oropharyngeal cancer || ||
257	rs12283049	G	A	G	A	-0.0564566	-0.205	0.234433	0.275	FALSE	FALSE	FALSE	ieu-b-98	11	14690192	0.138		932	0.1396	Oropharyngeal cancer || id:ieu-b-98	Oropharyngeal cancer	Oropharyngeal cancer || ||
258	rs12324720	A	G	A	G	-0.0149159	0.234	0.174629	0.158	FALSE	FALSE	FALSE	ieu-b-98	15	64092140	0.154		932	0.127999	Oropharyngeal cancer || id:ieu-b-98	Oropharyngeal cancer	Oropharyngeal cancer || ||
259	rs12462826	A	G	A	G	-0.0132119	0.126	0.369566	0.33	FALSE	FALSE	FALSE	ieu-b-98	19	11957001	0.126		932	0.320551	Oropharyngeal cancer || id:ieu-b-98	Oropharyngeal cancer	Oropharyngeal cancer || ||
260	rs12501515	A	G	A	G	-0.078957	-0.127	0.58966	0.54	FALSE	FALSE	FALSE	ieu-b-98	4	72592838	0.119		932	0.286168	Oropharyngeal cancer || id:ieu-b-98	Oropharyngeal cancer	Oropharyngeal cancer || ||
261	rs1260326	C	T	C	T	0.0197194	-0.021	0.603925	0.58	FALSE	FALSE	FALSE	ieu-b-98	2	27730940	0.12		932	0.860277	Oropharyngeal cancer || id:ieu-b-98	Oropharyngeal cancer	Oropharyngeal cancer || ||
262	rs12775091	T	C	T	C	0.0155618	-0.098	0.213441	0.191	FALSE	FALSE	FALSE	ieu-b-98	10	91543551	0.15		932	0.515409	Oropharyngeal cancer || id:ieu-b-98	Oropharyngeal cancer	Oropharyngeal cancer || ||
263	rs13076508	C	T	C	T	0.0250544	-0.545	0.0535034	0.064	FALSE	FALSE	FALSE	ieu-b-98	3	52407805	0.275		932	0.0476947	Oropharyngeal cancer || id:ieu-b-98	Oropharyngeal cancer	Oropharyngeal cancer || ||
264	rs13108245	G	A	G	A	-0.0122246	0.097	0.386601	0.395	FALSE	FALSE	FALSE	ieu-b-98	4	57790205	0.117		932	0.409219	Oropharyngeal cancer || id:ieu-b-98	Oropharyngeal cancer	Oropharyngeal cancer || ||
265	rs1321247	T	A	T	A	-0.0221847	0.359	0.101578	0.049	FALSE	TRUE	FALSE	ieu-b-98	6	25662873	0.252		932	0.15447	Oropharyngeal cancer || id:ieu-b-98	Oropharyngeal cancer	Oropharyngeal cancer || ||
266	rs13294734	T	C	T	C	0.012568	0.047	0.466128	0.438	FALSE	FALSE	FALSE	ieu-b-98	9	80710910	0.12		932	0.696583	Oropharyngeal cancer || id:ieu-b-98	Oropharyngeal cancer	Oropharyngeal cancer || ||
267	rs1343776	A	G	A	G	0.0180762	0.093	0.22133	0.189	FALSE	FALSE	FALSE	ieu-b-98	1	41757718	0.145		932	0.523277	Oropharyngeal cancer || id:ieu-b-98	Oropharyngeal cancer	Oropharyngeal cancer || ||
268	rs138335	G	C	G	C	-0.0137663	-0.069	0.658587	0.673	FALSE	TRUE	FALSE	ieu-b-98	22	41227086	0.122		932	0.566203	Oropharyngeal cancer || id:ieu-b-98	Oropharyngeal cancer	Oropharyngeal cancer || ||
269	rs1384687	A	G	A	G	-0.0168656	0.088	0.132271	0.174	FALSE	FALSE	FALSE	ieu-b-98	8	61525963	0.155		932	0.570435	Oropharyngeal cancer || id:ieu-b-98	Oropharyngeal cancer	Oropharyngeal cancer || ||
270	rs142004400	C	A	C	A	-0.0310034	0.343	0.0342066	0.021	FALSE	FALSE	FALSE	ieu-b-98	14	50829560	0.398		932	0.389528	Oropharyngeal cancer || id:ieu-b-98	Oropharyngeal cancer	Oropharyngeal cancer || ||
271	rs142158911	A	G	A	G	0.026284	0.002	0.111826	0.136	FALSE	FALSE	FALSE	ieu-b-98	19	11190534	0.165		932	0.989277	Oropharyngeal cancer || id:ieu-b-98	Oropharyngeal cancer	Oropharyngeal cancer || ||
272	rs144965707	A	G	A	G	-0.0348143	0.033	0.0618296	0.082	FALSE	FALSE	FALSE	ieu-b-98	11	14059511	0.214		932	0.874872	Oropharyngeal cancer || id:ieu-b-98	Oropharyngeal cancer	Oropharyngeal cancer || ||
273	rs1627043	C	G	C	G	-0.0486441	0.149	0.0332414	0.043	FALSE	TRUE	FALSE	ieu-b-98	11	71110175	0.299		932	0.618479	Oropharyngeal cancer || id:ieu-b-98	Oropharyngeal cancer	Oropharyngeal cancer || ||
274	rs1684600	T	C	T	C	-0.0125301	0.138	0.298695	0.37	FALSE	FALSE	FALSE	ieu-b-98	16	4594671	0.127		932	0.277985	Oropharyngeal cancer || id:ieu-b-98	Oropharyngeal cancer	Oropharyngeal cancer || ||
275	rs17207784	C	T	C	T	-0.0134939	0.048	0.324195	0.352	FALSE	FALSE	FALSE	ieu-b-98	6	22768668	0.119		932	0.689935	Oropharyngeal cancer || id:ieu-b-98	Oropharyngeal cancer	Oropharyngeal cancer || ||

276	rs17473257	A	G	A	G	-0.0611372	0.545	0.0172466	0.009	FALSE	FALSE	FALSE	ieu-b-98	11	14283186	0.563		932	0.333061	Oropharyngeal cancer ‖ id:ieu-b-98	Oropharyngeal cancer	Oropharyngeal cancer ‖ ‖
277	rs1800588	T	C	T	C	-0.0305021	-0.098	0.214964	0.289	FALSE	FALSE	FALSE	ieu-b-98	15	58730498	0.138		932	0.478903	Oropharyngeal cancer ‖ id:ieu-b-98	Oropharyngeal cancer	Oropharyngeal cancer ‖ ‖
278	rs1841850	C	A	C	A	0.0304395	0.245	0.117085	0.122	FALSE	FALSE	FALSE	ieu-b-98	20	52718555	0.176		932	0.165379	Oropharyngeal cancer ‖ id:ieu-b-98	Oropharyngeal cancer	Oropharyngeal cancer ‖ ‖
279	rs1858889	C	A	C	A	0.0134514	0.018	0.502532	0.478	FALSE	FALSE	FALSE	ieu-b-98	7	107117447	0.114		932	0.875503	Oropharyngeal cancer ‖ id:ieu-b-98	Oropharyngeal cancer	Oropharyngeal cancer ‖ ‖
280	rs1871395	G	A	G	A	-0.0203733	0.13	0.152679	0.155	FALSE	FALSE	FALSE	ieu-b-98	12	21352315	0.155		932	0.4011	Oropharyngeal cancer ‖ id:ieu-b-98	Oropharyngeal cancer	Oropharyngeal cancer ‖ ‖
281	rs1949633	C	T	C	T	0.0114162	-0.144	0.605759	0.629	FALSE	FALSE	FALSE	ieu-b-98	3	153758806	0.124		932	0.245503	Oropharyngeal cancer ‖ id:ieu-b-98	Oropharyngeal cancer	Oropharyngeal cancer ‖ ‖
282	rs2037511	A	G	A	G	0.0176624	0.187	0.165924	0.177	FALSE	FALSE	FALSE	ieu-b-98	18	61366207	0.151		932	0.2151	Oropharyngeal cancer ‖ id:ieu-b-98	Oropharyngeal cancer	Oropharyngeal cancer ‖ ‖
283	rs2074735	C	G	C	G	0.0292667	0.247	0.0648365	0.075	FALSE	TRUE	FALSE	ieu-b-98	22	31535872	0.214		932	0.2484	Oropharyngeal cancer ‖ id:ieu-b-98	Oropharyngeal cancer	Oropharyngeal cancer ‖ ‖
284	rs2171427	A	G	A	G	-0.0165489	-0.095	0.156491	0.228	FALSE	FALSE	FALSE	ieu-b-98	12	24822366	0.142		932	0.504089	Oropharyngeal cancer ‖ id:ieu-b-98	Oropharyngeal cancer	Oropharyngeal cancer ‖ ‖
285	rs2229742	C	G	C	G	-0.0249837	-0.054	0.104659	0.085	FALSE	TRUE	FALSE	ieu-b-98	21	16339172	0.22		932	0.804534	Oropharyngeal cancer ‖ id:ieu-b-98	Oropharyngeal cancer	Oropharyngeal cancer ‖ ‖
286	rs2245133	C	T	C	T	-0.0212917	-0.123	0.164339	0.163	FALSE	FALSE	FALSE	ieu-b-98	6	131931092	0.163		932	0.447281	Oropharyngeal cancer ‖ id:ieu-b-98	Oropharyngeal cancer	Oropharyngeal cancer ‖ ‖
287	rs2297991	C	T	C	T	0.0127547	-0.028	0.718478	0.672	FALSE	FALSE	FALSE	ieu-b-98	10	113913222	0.124		932	0.81607	Oropharyngeal cancer ‖ id:ieu-b-98	Oropharyngeal cancer	Oropharyngeal cancer ‖ ‖
288	rs2398113	G	A	G	A	-0.0117606	-0.07	0.423526	0.382	FALSE	FALSE	FALSE	ieu-b-98	10	10078742	0.139		932	0.615015	Oropharyngeal cancer ‖ id:ieu-b-98	Oropharyngeal cancer	Oropharyngeal cancer ‖ ‖
289	rs2494429	G	A	G	A	-0.0148459	0.034	0.822999	0.787	FALSE	FALSE	FALSE	ieu-b-98	1	2339395	0.153		932	0.824179	Oropharyngeal cancer ‖ id:ieu-b-98	Oropharyngeal cancer	Oropharyngeal cancer ‖ ‖
290	rs2511279	G	C	G	C	0.0981721	0.196	0.960373	0.861	FALSE	TRUE	FALSE	ieu-b-98	11	71130419	0.176		932	0.26688	Oropharyngeal cancer ‖ id:ieu-b-98	Oropharyngeal cancer	Oropharyngeal cancer ‖ ‖
291	rs2595644	T	G	T	G	-0.0122625	0.1	0.384889	0.428	FALSE	FALSE	FALSE	ieu-b-98	7	43980540	0.119		932	0.399091	Oropharyngeal cancer ‖ id:ieu-b-98	Oropharyngeal cancer	Oropharyngeal cancer ‖ ‖
292	rs2710651	A	G	A	G	-0.0115892	0.098	0.526101	0.512	FALSE	FALSE	FALSE	ieu-b-98	2	63166379	0.114		932	0.3912	Oropharyngeal cancer ‖ id:ieu-b-98	Oropharyngeal cancer	Oropharyngeal cancer ‖ ‖
293	rs2756119	A	G	A	G	0.0121434	-0.029	0.38143	0.39	FALSE	FALSE	FALSE	ieu-b-98	14	104001517	0.123		932	0.813887	Oropharyngeal cancer ‖ id:ieu-b-98	Oropharyngeal cancer	Oropharyngeal cancer ‖ ‖
294	rs2807834	G	T	G	T	-0.0150625	0.333	0.68514	0.719	FALSE	FALSE	FALSE	ieu-b-98	1	220970593	0.136		932	0.0144667	Oropharyngeal cancer ‖ id:ieu-b-98	Oropharyngeal cancer	Oropharyngeal cancer ‖ ‖
295	rs28435470	A	G	A	G	-0.0118696	-0.045	0.663204	0.689	FALSE	FALSE	FALSE	ieu-b-98	12	133076439	0.129		932	0.727647	Oropharyngeal cancer ‖ id:ieu-b-98	Oropharyngeal cancer	Oropharyngeal cancer ‖ ‖
296	rs2847500	A	G	A	G	-0.022548	0.115	0.123204	0.172	FALSE	FALSE	FALSE	ieu-b-98	11	120114421	0.159		932	0.471199	Oropharyngeal cancer ‖ id:ieu-b-98	Oropharyngeal cancer	Oropharyngeal cancer ‖ ‖
297	rs290400	A	G	A	G	-0.0130967	0.001	0.665472	0.706	FALSE	FALSE	FALSE	ieu-b-98	20	52698179	0.132		932	0.996652	Oropharyngeal cancer ‖ id:ieu-b-98	Oropharyngeal cancer	Oropharyngeal cancer ‖ ‖
298	rs3114045	C	T	C	T	-0.0221737	0.07	0.866137	0.814	FALSE	FALSE	FALSE	ieu-b-98	4	100252560	0.154		932	0.645523	Oropharyngeal cancer ‖ id:ieu-b-98	Oropharyngeal cancer	Oropharyngeal cancer ‖ ‖
299	rs325393	T	G	T	G	-0.0136497	-0.091	0.278218	0.259	FALSE	FALSE	FALSE	ieu-b-98	15	100229260	0.138		932	0.508155	Oropharyngeal cancer ‖ id:ieu-b-98	Oropharyngeal cancer	Oropharyngeal cancer ‖ ‖
300	rs34186890	G	A	G	A	-0.0156853	-0.027	0.259637	0.246	FALSE	FALSE	FALSE	ieu-b-98	3	141720712	0.138		932	0.841177	Oropharyngeal cancer ‖ id:ieu-b-98	Oropharyngeal cancer	Oropharyngeal cancer ‖ ‖
301	rs34726834	T	C	T	C	0.014013	-0.121	0.252209	0.251	FALSE	FALSE	FALSE	ieu-b-98	8	25889606	0.138		932	0.382174	Oropharyngeal cancer ‖ id:ieu-b-98	Oropharyngeal cancer	Oropharyngeal cancer ‖ ‖
302	rs35270497	T	C	T	C	0.0156723	0.31	0.176236	0.092	FALSE	FALSE	FALSE	ieu-b-98	2	38259872	0.196		932	0.114016	Oropharyngeal cancer ‖ id:ieu-b-98	Oropharyngeal cancer	Oropharyngeal cancer ‖ ‖
303	rs35823191	C	T	C	T	-0.0232636	0.08	0.342049	0.395	FALSE	FALSE	FALSE	ieu-b-98	1	17560123	0.12		932	0.505998	Oropharyngeal cancer ‖ id:ieu-b-98	Oropharyngeal cancer	Oropharyngeal cancer ‖ ‖
304	rs3732220	A	G	A	G	-0.0478406	-0.079	0.0852646	0.084	FALSE	FALSE	FALSE	ieu-b-98	2	234627048	0.21		932	0.706931	Oropharyngeal cancer ‖ id:ieu-b-98	Oropharyngeal cancer	Oropharyngeal cancer ‖ ‖
305	rs3829251	A	G	A	G	-0.114453	0.088	0.133277	0.225	FALSE	FALSE	FALSE	ieu-b-98	11	71194559	0.138		932	0.5247	Oropharyngeal cancer ‖ id:ieu-b-98	Oropharyngeal cancer	Oropharyngeal cancer ‖ ‖
306	rs4147536	C	A	C	A	-0.0148038	0.159	0.788613	0.772	FALSE	FALSE	FALSE	ieu-b-98	4	100239112	0.147		932	0.278245	Oropharyngeal cancer ‖ id:ieu-b-98	Oropharyngeal cancer	Oropharyngeal cancer ‖ ‖
307	rs4348160	G	T	G	T	-0.0258401	-0.029	0.326968	0.402	FALSE	FALSE	FALSE	ieu-b-98	4	70017531	0.117		932	0.797671	Oropharyngeal cancer ‖ id:ieu-b-98	Oropharyngeal cancer	Oropharyngeal cancer ‖ ‖
308	rs4364259	A	G	A	G	0.0172408	-0.42	0.198665	0.203	FALSE	FALSE	FALSE	ieu-b-98	4	15892159	0.179		932	0.0189186	Oropharyngeal cancer ‖ id:ieu-b-98	Oropharyngeal cancer	Oropharyngeal cancer ‖ ‖
309	rs4420638	G	A	G	A	-0.0192973	-0.431	0.176831	0.143	FALSE	FALSE	FALSE	ieu-b-98	19	45422946	0.194		928	0.0261102	Oropharyngeal cancer ‖ id:ieu-b-98	Oropharyngeal cancer	Oropharyngeal cancer ‖ ‖
310	rs4580037	C	A	C	A	-0.0135627	0.115	0.285578	0.279	FALSE	FALSE	FALSE	ieu-b-98	13	55702646	0.131		932	0.380167	Oropharyngeal cancer ‖ id:ieu-b-98	Oropharyngeal cancer	Oropharyngeal cancer ‖ ‖
311	rs512083	C	T	C	T	0.0122172	-0.145	0.462488	0.534	FALSE	FALSE	FALSE	ieu-b-98	1	46027355	0.116		932	0.210485	Oropharyngeal cancer ‖ id:ieu-b-98	Oropharyngeal cancer	Oropharyngeal cancer ‖ ‖
312	rs57601828	T	A	T	A	0.011542	-0.032	0.394508	0.333	FALSE	TRUE	FALSE	ieu-b-98	12	93204367	0.126		932	0.797815	Oropharyngeal cancer ‖ id:ieu-b-98	Oropharyngeal cancer	Oropharyngeal cancer ‖ ‖
313	rs5770794	T	C	T	C	-0.0133141	-0.087	0.3143	0.336	FALSE	FALSE	FALSE	ieu-b-98	22	50880781	0.127		932	0.492792	Oropharyngeal cancer ‖ id:ieu-b-98	Oropharyngeal cancer	Oropharyngeal cancer ‖ ‖
314	rs6129648	G	A	G	A	0.0140631	0.031	0.379837	0.348	FALSE	FALSE	FALSE	ieu-b-98	20	39231118	0.121		932	0.795663	Oropharyngeal cancer ‖ id:ieu-b-98	Oropharyngeal cancer	Oropharyngeal cancer ‖ ‖
315	rs61698755	C	T	C	T	-0.011465	0.198	0.560041	0.59	FALSE	FALSE	FALSE	ieu-b-98	17	79257880	0.119		932	0.0980325	Oropharyngeal cancer ‖ id:ieu-b-98	Oropharyngeal cancer	Oropharyngeal cancer ‖ ‖
316	rs61813875	G	C	G	C	0.0821291	-0.015	0.0248209	0.02	FALSE	TRUE	FALSE	ieu-b-98	1	152536650	0.486		932	0.97504	Oropharyngeal cancer ‖ id:ieu-b-98	Oropharyngeal cancer	Oropharyngeal cancer ‖ ‖
317	rs61887421	C	T	C	T	-0.036726	-0.685	0.0300871	0.039	FALSE	FALSE	FALSE	ieu-b-98	11	70949673	0.426		932	0.108058	Oropharyngeal cancer ‖ id:ieu-b-98	Oropharyngeal cancer	Oropharyngeal cancer ‖ ‖
318	rs62007299	A	G	A	G	-0.0124205	-0.074	0.712958	0.654	FALSE	FALSE	FALSE	ieu-b-98	15	77711719	0.121		932	0.543265	Oropharyngeal cancer ‖ id:ieu-b-98	Oropharyngeal cancer	Oropharyngeal cancer ‖ ‖
319	rs62129966	A	C	A	C	0.0611636	-0.152	0.160854	0.117	FALSE	FALSE	FALSE	ieu-b-98	19	48374950	0.194		932	0.433271	Oropharyngeal cancer ‖ id:ieu-b-98	Oropharyngeal cancer	Oropharyngeal cancer ‖ ‖
320	rs635634	T	C	T	C	-0.0150476	0.117	0.186573	0.195	FALSE	FALSE	FALSE	ieu-b-98	9	136155000	0.144		932	0.418418	Oropharyngeal cancer ‖ id:ieu-b-98	Oropharyngeal cancer	Oropharyngeal cancer ‖ ‖
321	rs6438900	G	C	G	C	0.0150486	-0.029	0.256034	0.29	FALSE	TRUE	FALSE	ieu-b-98	3	125148287	0.131		932	0.82311	Oropharyngeal cancer ‖ id:ieu-b-98	Oropharyngeal cancer	Oropharyngeal cancer ‖ ‖
322	rs6672758	T	C	T	C	0.0162478	0.094	0.80023	0.74	FALSE	FALSE	FALSE	ieu-b-98	1	230303512	0.14		932	0.50026	Oropharyngeal cancer ‖ id:ieu-b-98	Oropharyngeal cancer	Oropharyngeal cancer ‖ ‖
323	rs6834488	T	C	T	C	-0.01445	0.018	0.422833	0.426	FALSE	FALSE	FALSE	ieu-b-98	4	88178919	0.118		932	0.882348	Oropharyngeal cancer ‖ id:ieu-b-98	Oropharyngeal cancer	Oropharyngeal cancer ‖ ‖
324	rs71599974	G	A	G	A	0.0257378	0.173	0.148048	0.14	FALSE	FALSE	FALSE	ieu-b-98	4	71765339	0.184		932	0.345454	Oropharyngeal cancer ‖ id:ieu-b-98	Oropharyngeal cancer	Oropharyngeal cancer ‖ ‖
325	rs727857	A	G	A	G	-0.0120548	0.151	0.61181	0.557	FALSE	FALSE	FALSE	ieu-b-98	2	58981967	0.119		932	0.202844	Oropharyngeal cancer ‖ id:ieu-b-98	Oropharyngeal cancer	Oropharyngeal cancer ‖ ‖
326	rs733454	T	C	T	C	0.0188545	0.102	0.0991663	0.043	FALSE	FALSE	FALSE	ieu-b-98	11	76477721	0.278		932	0.714204	Oropharyngeal cancer ‖ id:ieu-b-98	Oropharyngeal cancer	Oropharyngeal cancer ‖ ‖
327	rs742493	C	T	C	T	0.0183528	-0.053	0.112871	0.108	FALSE	FALSE	FALSE	ieu-b-98	6	40998167	0.195		932	0.782862	Oropharyngeal cancer ‖ id:ieu-b-98	Oropharyngeal cancer	Oropharyngeal cancer ‖ ‖
328	rs7528419	G	A	G	A	0.0215389	-0.081	0.22441	0.219	FALSE	FALSE	FALSE	ieu-b-98	1	109817192	0.14		930	0.5634	Oropharyngeal cancer ‖ id:ieu-b-98	Oropharyngeal cancer	Oropharyngeal cancer ‖ ‖
329	rs7569755	A	G	A	G	0.0136395	-0.252	0.288618	0.294	FALSE	FALSE	FALSE	ieu-b-98	2	118648261	0.134		932	0.0597035	Oropharyngeal cancer ‖ id:ieu-b-98	Oropharyngeal cancer	Oropharyngeal cancer ‖ ‖
330	rs7580771	T	G	T	G	-0.0165625	0.075	0.175983	0.246	FALSE	FALSE	FALSE	ieu-b-98	2	101428119	0.131		932	0.566178	Oropharyngeal cancer ‖ id:ieu-b-98	Oropharyngeal cancer	Oropharyngeal cancer ‖ ‖
331	rs7652808	G	T	G	T	-0.0212858	0.107	0.649492	0.585	FALSE	FALSE	FALSE	ieu-b-98	3	85604320	0.122		932	0.382722	Oropharyngeal cancer ‖ id:ieu-b-98	Oropharyngeal cancer	Oropharyngeal cancer ‖ ‖
332	rs7712001	G	T	G	T	0.011939	0.079	0.440176	0.426	FALSE	FALSE	FALSE	ieu-b-98	5	148020950	0.121		932	0.517906	Oropharyngeal cancer ‖ id:ieu-b-98	Oropharyngeal cancer	Oropharyngeal cancer ‖ ‖
333	rs7784802	T	A	T	A	0.0133202	0.212	0.358913	0.269	FALSE	TRUE	FALSE	ieu-b-98	7	64020652	0.126		932	0.0935169	Oropharyngeal cancer ‖ id:ieu-b-98	Oropharyngeal cancer	Oropharyngeal cancer ‖ ‖
334	rs77924615	A	G	A	G	-0.0152475	0	0.194374	0.203	FALSE	FALSE	FALSE	ieu-b-98	16	20392332	0.157		932	0.999557	Oropharyngeal cancer ‖ id:ieu-b-98	Oropharyngeal cancer	Oropharyngeal cancer ‖ ‖
335	rs77960347	G	A	G	A	-0.0525688	0.086	0.0127013	0.017	FALSE	FALSE	FALSE	ieu-b-98	18	47109955	0.453		932	0.849265	Oropharyngeal cancer ‖ id:ieu-b-98	Oropharyngeal cancer	Oropharyngeal cancer ‖ ‖
336	rs78649910	A	T	A	T	-0.0191377	-0.074	0.105728	0.114	FALSE	TRUE	FALSE	ieu-b-98	4	3482213	0.199		932	0.711992	Oropharyngeal cancer ‖ id:ieu-b-98	Oropharyngeal cancer	Oropharyngeal cancer ‖ ‖
337	rs7955128	T	A	T	A	0.0130617	-0.098	0.52053	0.541	FALSE	TRUE	TRUE	ieu-b-98	12	38684121	0.121		932	0.417516	Oropharyngeal cancer ‖ id:ieu-b-98	Oropharyngeal cancer	Oropharyngeal cancer ‖ ‖
338	rs8018720	C	G	C	G	-0.0344962	0.005	0.823508	0.838	FALSE	TRUE	FALSE	ieu-b-98	14	39556185	0.154		932	0.9747	Oropharyngeal cancer ‖ id:ieu-b-98	Oropharyngeal cancer	Oropharyngeal cancer ‖ ‖
339	rs804281	G	A	G	A	0.015895	0.07	0.583498	0.63	FALSE	FALSE	FALSE	ieu-b-98	8	11611865	0.123		932	0.573455	Oropharyngeal cancer ‖ id:ieu-b-98	Oropharyngeal cancer	Oropharyngeal cancer ‖ ‖
340	rs8107974	T	A	T	A	0.0355672	0.066	0.0763013	0.068	FALSE	TRUE	FALSE	ieu-b-98	19	19388500	0.225		932	0.769607	Oropharyngeal cancer ‖ id:ieu-b-98	Oropharyngeal cancer	Oropharyngeal cancer ‖ ‖
341	rs8121940	G	C	G	C	-0.0435612	0.148	0.19762	0.246	FALSE	TRUE	FALSE	ieu-b-98	20	52742306	0.159		932	0.350143	Oropharyngeal cancer ‖ id:ieu-b-98	Oropharyngeal cancer	Oropharyngeal cancer ‖ ‖
342	rs9375037	C	A	C	A	0.0117059	-0.021	0.443168	0.44	FALSE	FALSE	FALSE	ieu-b-98	6	121856794	0.114		932	0.850392	Oropharyngeal cancer ‖ id:ieu-b-98	Oropharyngeal cancer	Oropharyngeal cancer ‖ ‖
343	rs9409266	A	G	A	G	-0.0167771	-0.02	0.862222	0.794	FALSE	FALSE	FALSE	ieu-b-98	9	125824055	0.147		932	0.888702	Oropharyngeal cancer ‖ id:ieu-b-98	Oropharyngeal cancer	Oropharyngeal cancer ‖ ‖
344	rs964184	C	G	C	G	0.0406845	0.078	0.867234	0.814	FALSE	TRUE	FALSE	ieu-b-98	11	116648917	0.152		932	0.6051	Oropharyngeal cancer ‖ id:ieu-b-98	Oropharyngeal cancer	Oropharyngeal cancer ‖ ‖
345	rs9847248	A	G	A	G	-0.0123077	-0.035	0.712969	0.704	FALSE	FALSE	FALSE	ieu-b-98	3	18804655	0.125		932	0.783266	Oropharyngeal cancer ‖ id:ieu-b-98	Oropharyngeal cancer	Oropharyngeal cancer ‖ ‖
346	rs986649	G	A	G	A	0.0128637	0.035	0.321628	0.27	FALSE	FALSE	FALSE	ieu-b-98	5	118668050	0.133		932	0.788199	Oropharyngeal cancer ‖ id:ieu-b-98	Oropharyngeal cancer	Oropharyngeal cancer ‖ ‖
347	rs9946771	T	C	T	C	-0.0233992	0.317	0.0663426	0.117	FALSE	FALSE	FALSE	ieu-b-98	18	28918628	0.17		932	0.0626874	Oropharyngeal cancer ‖ id:ieu-b-98	Oropharyngeal cancer	Oropharyngeal cancer ‖ ‖
348	rs11172066	T	A	T	A	0.166	-0.146	0.14	0.168	FALSE	TRUE	FALSE	ieu-b-98	12	57319491	0.173		932	0.399584	Oropharyngeal cancer ‖ id:ieu-b-98	Oropharyngeal cancer	Oropharyngeal cancer ‖ ‖
349	rs28364617	G	T	G	T	0.131	0.05	0.72	0.566	FALSE	FALSE	FALSE	ieu-b-98	11	71159764	0.121		932	0.683452	Oropharyngeal cancer ‖ id:ieu-b-98	Oropharyngeal cancer	Oropharyngeal cancer ‖ ‖
350	rs116970203	G	A	G	A	0.372	0.265	0.98	0.988	FALSE	FALSE	FALSE	ieu-b-98	11	14876718	0.59		932	0.653304	Oropharyngeal cancer ‖ id:ieu-b-98	Oropharyngeal cancer	Oropharyngeal cancer ‖ ‖
351	rs13084927	C	A	C	A	0.055	-0.25	0.83	0.85	FALSE	FALSE	FALSE	ieu-b-98	3	72709792	0.161		932	0.119846	Oropharyngeal cancer ‖ id:ieu-b-98	Oropharyngeal cancer	Oropharyngeal cancer ‖ ‖
352	rs17216707	T	C	T	C	0.074	-0.222	0.8	0.77	FALSE	FALSE	FALSE	ieu-b-98	20	52732362	0.16		932	0.165053	Oropharyngeal cancer ‖ id:ieu-b-98	Oropharyngeal cancer	Oropharyngeal cancer ‖ ‖
353	rs28364617	G	T	G	T	0.127	0.05	0.71	0.566	FALSE	FALSE	FALSE	ieu-b-98	11	71159764	0.121		932	0.683452	Oropharyngeal cancer ‖ id:ieu-b-98	Oropharyngeal cancer	Oropharyngeal cancer ‖ ‖
354	rs3819817	C	T	C	T	0.058	-0.015	0.45	0.507	FALSE	FALSE	FALSE	ieu-b-98	12	96378771	0.122		932	0.902233	Oropharyngeal cancer ‖ id:ieu-b-98	Oropharyngeal cancer	Oropharyngeal cancer ‖ ‖
355	rs9304669	T	C	T	C	0.054	-0.274	0.16	0.141	FALSE	FALSE	FALSE	ieu-b-98	19	48384385	0.187		932	0.141724	Oropharyngeal cancer ‖ id:ieu-b-98	Oropharyngeal cancer	Oropharyngeal cancer ‖ ‖

mr_keep.outcome	data_source.outcome	proxy.outcome	target_snp.outcome	proxy_snp.outcome	target_a1.outcome	target_a2.outcome	proxy_a1.outcome	proxy_a2.outcome	se.exposure	pval.exposure	exposure	mr_keep.exposure	pval_origin.exposure	id.exposure	data_source.exposure	action	mr_keep
TRUE	igd	NA	NA	NA	NA	NA	NA	NA	0.023	3.22E-60	25OHD3	TRUE	reported	20duX4	textfile	2	TRUE
TRUE	igd	NA	NA	NA	NA	NA	NA	NA	0.01	1.94E-08	25OHD3	TRUE	reported	20duX4	textfile	2	TRUE
TRUE	igd	NA	NA	NA	NA	NA	NA	NA	0.009	1.09E-15	25OHD3	TRUE	reported	20duX4	textfile	2	TRUE
TRUE	igd	NA	NA	NA	NA	NA	NA	NA	0.008	4.08E-59	25OHD3	TRUE	reported	20duX4	textfile	2	TRUE
TRUE	igd	NA	NA	NA	NA	NA	NA	NA	0.007	3.59E-16	25OHD3	TRUE	reported	20duX4	textfile	2	TRUE
TRUE	igd	NA	NA	NA	NA	NA	NA	NA	0.01	1.27E-08	25OHD3	TRUE	reported	20duX4	textfile	2	TRUE
TRUE	igd	NA	NA	NA	NA	NA	NA	NA	0.0023507	1.08E-09	Serum 25-Hydroxyvitamin D levels || id:ebi-a-GCST90000618	TRUE	reported	WO8j2s	textfile	2	TRUE
TRUE	igd	NA	NA	NA	NA	NA	NA	NA	0.00205629	2.15E-08	Serum 25-Hydroxyvitamin D levels || id:ebi-a-GCST90000618	TRUE	reported	WO8j2s	textfile	2	TRUE
TRUE	igd	TRUE	rs1042034	rs2678379	C	T	A	G	0.00250017	1.45E-09	Serum 25-Hydroxyvitamin D levels || id:ebi-a-GCST90000618	TRUE	reported	WO8j2s	textfile	2	TRUE
TRUE	igd	NA	NA	NA	NA	NA	NA	NA	0.00264437	7.34E-11	Serum 25-Hydroxyvitamin D levels || id:ebi-a-GCST90000618	TRUE	reported	WO8j2s	textfile	2	TRUE
TRUE	igd	NA	NA	NA	NA	NA	NA	NA	0.00218016	7.96E-10	Serum 25-Hydroxyvitamin D levels || id:ebi-a-GCST90000618	TRUE	reported	WO8j2s	textfile	2	TRUE
TRUE	igd	NA	NA	NA	NA	NA	NA	NA	0.00374376	5.58E-17	Serum 25-Hydroxyvitamin D levels || id:ebi-a-GCST90000618	TRUE	reported	WO8j2s	textfile	2	TRUE
TRUE	igd	TRUE	rs10859995	rs3819817	T	C	C	T	0.00205452	4.60E-100	Serum 25-Hydroxyvitamin D levels || id:ebi-a-GCST90000618	TRUE	reported	WO8j2s	textfile	2	TRUE
TRUE	igd	NA	NA	NA	NA	NA	NA	NA	0.00572507	3.73E-17	Serum 25-Hydroxyvitamin D levels || id:ebi-a-GCST90000618	TRUE	reported	WO8j2s	textfile	2	TRUE
TRUE	igd	NA	NA	NA	NA	NA	NA	NA	0.00266997	9.64E-18	Serum 25-Hydroxyvitamin D levels || id:ebi-a-GCST90000618	TRUE	reported	WO8j2s	textfile	2	TRUE
TRUE	igd	NA	NA	NA	NA	NA	NA	NA	0.00212672	7.14E-23	Serum 25-Hydroxyvitamin D levels || id:ebi-a-GCST90000618	TRUE	reported	WO8j2s	textfile	2	TRUE
TRUE	igd	NA	NA	NA	NA	NA	NA	NA	0.00234081	7.97E-14	Serum 25-Hydroxyvitamin D levels || id:ebi-a-GCST90000618	TRUE	reported	WO8j2s	textfile	2	TRUE
TRUE	igd	NA	NA	NA	NA	NA	NA	NA	0.0020293	6.06E-16	Serum 25-Hydroxyvitamin D levels || id:ebi-a-GCST90000618	TRUE	reported	WO8j2s	textfile	2	TRUE
TRUE	igd	NA	NA	NA	NA	NA	NA	NA	0.00498229	2.36E-56	Serum 25-Hydroxyvitamin D levels || id:ebi-a-GCST90000618	TRUE	reported	WO8j2s	textfile	2	TRUE
TRUE	igd	NA	NA	NA	NA	NA	NA	NA	0.00298265	9.72E-17	Serum 25-Hydroxyvitamin D levels || id:ebi-a-GCST90000618	TRUE	reported	WO8j2s	textfile	2	TRUE
TRUE	igd	NA	NA	NA	NA	NA	NA	NA	0.00225483	3.08E-125	Serum 25-Hydroxyvitamin D levels || id:ebi-a-GCST90000618	TRUE	reported	WO8j2s	textfile	2	TRUE
TRUE	igd	NA	NA	NA	NA	NA	NA	NA	0.00258052	4.85E-08	Serum 25-Hydroxyvitamin D levels || id:ebi-a-GCST90000618	TRUE	reported	WO8j2s	textfile	2	TRUE
TRUE	igd	NA	NA	NA	NA	NA	NA	NA	0.00209454	1.01E-10	Serum 25-Hydroxyvitamin D levels || id:ebi-a-GCST90000618	TRUE	reported	WO8j2s	textfile	2	TRUE
TRUE	igd	NA	NA	NA	NA	NA	NA	NA	0.00206406	2.65E-29	Serum 25-Hydroxyvitamin D levels || id:ebi-a-GCST90000618	TRUE	reported	WO8j2s	textfile	2	TRUE
TRUE	igd	NA	NA	NA	NA	NA	NA	NA	0.0030906	8.16E-09	Serum 25-Hydroxyvitamin D levels || id:ebi-a-GCST90000618	TRUE	reported	WO8j2s	textfile	2	TRUE
TRUE	igd	NA	NA	NA	NA	NA	NA	NA	0.00240614	9.62E-122	Serum 25-Hydroxyvitamin D levels || id:ebi-a-GCST90000618	TRUE	reported	WO8j2s	textfile	2	TRUE
TRUE	igd	NA	NA	NA	NA	NA	NA	NA	0.00267451	2.45E-08	Serum 25-Hydroxyvitamin D levels || id:ebi-a-GCST90000618	TRUE	reported	WO8j2s	textfile	2	TRUE
TRUE	igd	TRUE	rs12462826	rs2607454	A	G	T	G	0.0021149	4.18E-10	Serum 25-Hydroxyvitamin D levels || id:ebi-a-GCST90000618	TRUE	reported	WO8j2s	textfile	2	TRUE
TRUE	igd	NA	NA	NA	NA	NA	NA	NA	0.00206932	1.00E-200	Serum 25-Hydroxyvitamin D levels || id:ebi-a-GCST90000618	TRUE	reported	WO8j2s	textfile	2	TRUE
TRUE	igd	NA	NA	NA	NA	NA	NA	NA	0.00207421	1.96E-21	Serum 25-Hydroxyvitamin D levels || id:ebi-a-GCST90000618	TRUE	reported	WO8j2s	textfile	2	TRUE
TRUE	igd	TRUE	rs12775091	rs34408087	T	C	A	G	0.00247693	3.33E-10	Serum 25-Hydroxyvitamin D levels || id:ebi-a-GCST90000618	TRUE	reported	WO8j2s	textfile	2	TRUE
TRUE	igd	NA	NA	NA	NA	NA	NA	NA	0.00451036	2.78E-08	Serum 25-Hydroxyvitamin D levels || id:ebi-a-GCST90000618	TRUE	reported	WO8j2s	textfile	2	TRUE
TRUE	igd	NA	NA	NA	NA	NA	NA	NA	0.00208616	4.63E-09	Serum 25-Hydroxyvitamin D levels || id:ebi-a-GCST90000618	TRUE	reported	WO8j2s	textfile	2	TRUE
TRUE	igd	NA	NA	NA	NA	NA	NA	NA	0.00336578	4.36E-11	Serum 25-Hydroxyvitamin D levels || id:ebi-a-GCST90000618	TRUE	reported	WO8j2s	textfile	2	TRUE
TRUE	igd	NA	NA	NA	NA	NA	NA	NA	0.00205837	1.02E-09	Serum 25-Hydroxyvitamin D levels || id:ebi-a-GCST90000618	TRUE	reported	WO8j2s	textfile	2	TRUE
TRUE	igd	NA	NA	NA	NA	NA	NA	NA	0.00245028	1.62E-13	Serum 25-Hydroxyvitamin D levels || id:ebi-a-GCST90000618	TRUE	reported	WO8j2s	textfile	2	TRUE
TRUE	igd	NA	NA	NA	NA	NA	NA	NA	0.00215115	1.56E-10	Serum 25-Hydroxyvitamin D levels || id:ebi-a-GCST90000618	TRUE	reported	WO8j2s	textfile	2	TRUE
TRUE	igd	NA	NA	NA	NA	NA	NA	NA	0.00299675	1.82E-08	Serum 25-Hydroxyvitamin D levels || id:ebi-a-GCST90000618	TRUE	reported	WO8j2s	textfile	2	TRUE
TRUE	igd	NA	NA	NA	NA	NA	NA	NA	0.00559561	3.01E-08	Serum 25-Hydroxyvitamin D levels || id:ebi-a-GCST90000618	TRUE	reported	WO8j2s	textfile	2	TRUE
TRUE	igd	NA	NA	NA	NA	NA	NA	NA	0.00323446	4.43E-16	Serum 25-Hydroxyvitamin D levels || id:ebi-a-GCST90000618	TRUE	reported	WO8j2s	textfile	2	TRUE
TRUE	igd	NA	NA	NA	NA	NA	NA	NA	0.00421754	1.52E-16	Serum 25-Hydroxyvitamin D levels || id:ebi-a-GCST90000618	TRUE	reported	WO8j2s	textfile	2	TRUE
TRUE	igd	NA	NA	NA	NA	NA	NA	NA	0.00566106	8.49E-18	Serum 25-Hydroxyvitamin D levels || id:ebi-a-GCST90000618	TRUE	reported	WO8j2s	textfile	2	TRUE
TRUE	igd	NA	NA	NA	NA	NA	NA	NA	0.00221719	1.59E-08	Serum 25-Hydroxyvitamin D levels || id:ebi-a-GCST90000618	TRUE	reported	WO8j2s	textfile	2	TRUE
TRUE	igd	NA	NA	NA	NA	NA	NA	NA	0.0021713	5.14E-10	Serum 25-Hydroxyvitamin D levels || id:ebi-a-GCST90000618	TRUE	reported	WO8j2s	textfile	2	TRUE
TRUE	igd	NA	NA	NA	NA	NA	NA	NA	0.00780044	4.59E-15	Serum 25-Hydroxyvitamin D levels || id:ebi-a-GCST90000618	TRUE	reported	WO8j2s	textfile	2	TRUE
TRUE	igd	TRUE	rs1800588	rs588136	T	C	C	T	0.00246932	4.73E-35	Serum 25-Hydroxyvitamin D levels || id:ebi-a-GCST90000618	TRUE	reported	WO8j2s	textfile	2	TRUE
TRUE	igd	TRUE	rs1841850	rs8123293	C	A	G	A	0.00316491	6.73E-22	Serum 25-Hydroxyvitamin D levels || id:ebi-a-GCST90000618	TRUE	reported	WO8j2s	textfile	2	TRUE
TRUE	igd	NA	NA	NA	NA	NA	NA	NA	0.00203065	3.49E-11	Serum 25-Hydroxyvitamin D levels || id:ebi-a-GCST90000618	TRUE	reported	WO8j2s	textfile	2	TRUE
TRUE	igd	NA	NA	NA	NA	NA	NA	NA	0.00282689	5.72E-13	Serum 25-Hydroxyvitamin D levels || id:ebi-a-GCST90000618	TRUE	reported	WO8j2s	textfile	2	TRUE
TRUE	igd	NA	NA	NA	NA	NA	NA	NA	0.00208637	4.45E-08	Serum 25-Hydroxyvitamin D levels || id:ebi-a-GCST90000618	TRUE	reported	WO8j2s	textfile	2	TRUE
TRUE	igd	NA	NA	NA	NA	NA	NA	NA	0.00272732	9.41E-11	Serum 25-Hydroxyvitamin D levels || id:ebi-a-GCST90000618	TRUE	reported	WO8j2s	textfile	2	TRUE
TRUE	igd	NA	NA	NA	NA	NA	NA	NA	0.00412	1.22E-12	Serum 25-Hydroxyvitamin D levels || id:ebi-a-GCST90000618	TRUE	reported	WO8j2s	textfile	2	TRUE
TRUE	igd	TRUE	rs2171427	rs11047561	A	G	G	C	0.00281738	4.26E-09	Serum 25-Hydroxyvitamin D levels || id:ebi-a-GCST90000618	TRUE	reported	WO8j2s	textfile	2	TRUE
TRUE	igd	NA	NA	NA	NA	NA	NA	NA	0.00331409	4.75E-14	Serum 25-Hydroxyvitamin D levels || id:ebi-a-GCST90000618	TRUE	reported	WO8j2s	textfile	2	TRUE
TRUE	igd	NA	NA	NA	NA	NA	NA	NA	0.00273999	7.80E-15	Serum 25-Hydroxyvitamin D levels || id:ebi-a-GCST90000618	TRUE	reported	WO8j2s	textfile	2	TRUE
TRUE	igd	NA	NA	NA	NA	NA	NA	NA	0.00225578	1.57E-08	Serum 25-Hydroxyvitamin D levels || id:ebi-a-GCST90000618	TRUE	reported	WO8j2s	textfile	2	TRUE
TRUE	igd	TRUE	rs2398113	rs17147117	G	A	C	A	0.00205797	1.10E-08	Serum 25-Hydroxyvitamin D levels || id:ebi-a-GCST90000618	TRUE	reported	WO8j2s	textfile	2	TRUE
TRUE	igd	NA	NA	NA	NA	NA	NA	NA	0.00267333	2.80E-08	Serum 25-Hydroxyvitamin D levels || id:ebi-a-GCST90000618	TRUE	reported	WO8j2s	textfile	2	TRUE
TRUE	igd	NA	NA	NA	NA	NA	NA	NA	0.00520826	2.98E-79	Serum 25-Hydroxyvitamin D levels || id:ebi-a-GCST90000618	TRUE	reported	WO8j2s	textfile	2	TRUE
TRUE	igd	NA	NA	NA	NA	NA	NA	NA	0.00209685	4.97E-09	Serum 25-Hydroxyvitamin D levels || id:ebi-a-GCST90000618	TRUE	reported	WO8j2s	textfile	2	TRUE
TRUE	igd	NA	NA	NA	NA	NA	NA	NA	0.00203474	1.23E-08	Serum 25-Hydroxyvitamin D levels || id:ebi-a-GCST90000618	TRUE	reported	WO8j2s	textfile	2	TRUE
TRUE	igd	NA	NA	NA	NA	NA	NA	NA	0.0021104	8.71E-09	Serum 25-Hydroxyvitamin D levels || id:ebi-a-GCST90000618	TRUE	reported	WO8j2s	textfile	2	TRUE
TRUE	igd	NA	NA	NA	NA	NA	NA	NA	0.00218678	5.66E-12	Serum 25-Hydroxyvitamin D levels || id:ebi-a-GCST90000618	TRUE	reported	WO8j2s	textfile	2	TRUE
TRUE	igd	TRUE	rs28435470	rs61952071	G	A	A	C	0.00214831	3.29E-08	Serum 25-Hydroxyvitamin D levels || id:ebi-a-GCST90000618	TRUE	reported	WO8j2s	textfile	2	TRUE
TRUE	igd	NA	NA	NA	NA	NA	NA	NA	0.00308665	2.77E-13	Serum 25-Hydroxyvitamin D levels || id:ebi-a-GCST90000618	TRUE	reported	WO8j2s	textfile	2	TRUE
TRUE	igd	NA	NA	NA	NA	NA	NA	NA	0.00216338	1.41E-09	Serum 25-Hydroxyvitamin D levels || id:ebi-a-GCST90000618	TRUE	reported	WO8j2s	textfile	2	TRUE
TRUE	igd	NA	NA	NA	NA	NA	NA	NA	0.00298008	1.00E-13	Serum 25-Hydroxyvitamin D levels || id:ebi-a-GCST90000618	TRUE	reported	WO8j2s	textfile	2	TRUE
TRUE	igd	NA	NA	NA	NA	NA	NA	NA	0.00227676	2.03E-09	Serum 25-Hydroxyvitamin D levels || id:ebi-a-GCST90000618	TRUE	reported	WO8j2s	textfile	2	TRUE
TRUE	igd	NA	NA	NA	NA	NA	NA	NA	0.00231839	1.33E-11	Serum 25-Hydroxyvitamin D levels || id:ebi-a-GCST90000618	TRUE	reported	WO8j2s	textfile	2	TRUE
TRUE	igd	NA	NA	NA	NA	NA	NA	NA	0.00234858	2.42E-09	Serum 25-Hydroxyvitamin D levels || id:ebi-a-GCST90000618	TRUE	reported	WO8j2s	textfile	2	TRUE
TRUE	igd	NA	NA	NA	NA	NA	NA	NA	0.0026815	5.08E-09	Serum 25-Hydroxyvitamin D levels || id:ebi-a-GCST90000618	TRUE	reported	WO8j2s	textfile	2	TRUE
TRUE	igd	NA	NA	NA	NA	NA	NA	NA	0.00214072	1.65E-27	Serum 25-Hydroxyvitamin D levels || id:ebi-a-GCST90000618	TRUE	reported	WO8j2s	textfile	2	TRUE
TRUE	igd	NA	NA	NA	NA	NA	NA	NA	0.00363266	1.31E-39	Serum 25-Hydroxyvitamin D levels || id:ebi-a-GCST90000618	TRUE	reported	WO8j2s	textfile	2	TRUE
TRUE	igd	NA	NA	NA	NA	NA	NA	NA	0.00298056	1.00E-200	Serum 25-Hydroxyvitamin D levels || id:ebi-a-GCST90000618	TRUE	reported	WO8j2s	textfile	2	TRUE
TRUE	igd	NA	NA	NA	NA	NA	NA	NA	0.00249007	2.76E-09	Serum 25-Hydroxyvitamin D levels || id:ebi-a-GCST90000618	TRUE	reported	WO8j2s	textfile	2	TRUE
TRUE	igd	NA	NA	NA	NA	NA	NA	NA	0.00216263	6.62E-33	Serum 25-Hydroxyvitamin D levels || id:ebi-a-GCST90000618	TRUE	reported	WO8j2s	textfile	2	TRUE
TRUE	igd	NA	NA	NA	NA	NA	NA	NA	0.00256676	1.86E-11	Serum 25-Hydroxyvitamin D levels || id:ebi-a-GCST90000618	TRUE	reported	WO8j2s	textfile	2	TRUE
TRUE	igd	NA	NA	NA	NA	NA	NA	NA	0.00265905	3.95E-13	Serum 25-Hydroxyvitamin D levels || id:ebi-a-GCST90000618	TRUE	reported	WO8j2s	textfile	2	TRUE
TRUE	igd	NA	NA	NA	NA	NA	NA	NA	0.00225079	1.68E-09	Serum 25-Hydroxyvitamin D levels || id:ebi-a-GCST90000618	TRUE	reported	WO8j2s	textfile	2	TRUE
TRUE	igd	NA	NA	NA	NA	NA	NA	NA	0.00204286	2.23E-09	Serum 25-Hydroxyvitamin D levels || id:ebi-a-GCST90000618	TRUE	reported	WO8j2s	textfile	2	TRUE
TRUE	igd	TRUE	rs57601828	rs7977757	T	A	A	G	0.00208192	2.96E-08	Serum 25-Hydroxyvitamin D levels || id:ebi-a-GCST90000618	TRUE	reported	WO8j2s	textfile	2	TRUE
TRUE	igd	NA	NA	NA	NA	NA	NA	NA	0.00221143	1.74E-09	Serum 25-Hydroxyvitamin D levels || id:ebi-a-GCST90000618	TRUE	reported	WO8j2s	textfile	2	TRUE
TRUE	igd	NA	NA	NA	NA	NA	NA	NA	0.00210628	2.44E-11	Serum 25-Hydroxyvitamin D levels || id:ebi-a-GCST90000618	TRUE	reported	WO8j2s	textfile	2	TRUE
TRUE	igd	NA	NA	NA	NA	NA	NA	NA	0.0020505	2.25E-08	Serum 25-Hydroxyvitamin D levels || id:ebi-a-GCST90000618	TRUE	reported	WO8j2s	textfile	2	TRUE
TRUE	igd	NA	NA	NA	NA	NA	NA	NA	0.00658876	1.16E-35	Serum 25-Hydroxyvitamin D levels || id:ebi-a-GCST90000618	TRUE	reported	WO8j2s	textfile	2	TRUE
TRUE	igd	NA	NA	NA	NA	NA	NA	NA	0.00597754	8.05E-10	Serum 25-Hydroxyvitamin D levels || id:ebi-a-GCST90000618	TRUE	reported	WO8j2s	textfile	2	TRUE
TRUE	igd	NA	NA	NA	NA	NA	NA	NA	0.00224413	3.12E-08	Serum 25-Hydroxyvitamin D levels || id:ebi-a-GCST90000618	TRUE	reported	WO8j2s	textfile	2	TRUE
TRUE	igd	NA	NA	NA	NA	NA	NA	NA	0.00276373	1.60E-108	Serum 25-Hydroxyvitamin D levels || id:ebi-a-GCST90000618	TRUE	reported	WO8j2s	textfile	2	TRUE
TRUE	igd	NA	NA	NA	NA	NA	NA	NA	0.0026042	7.55E-09	Serum 25-Hydroxyvitamin D levels || id:ebi-a-GCST90000618	TRUE	reported	WO8j2s	textfile	2	TRUE
TRUE	igd	NA	NA	NA	NA	NA	NA	NA	0.00234031	1.27E-10	Serum 25-Hydroxyvitamin D levels || id:ebi-a-GCST90000618	TRUE	reported	WO8j2s	textfile	2	TRUE
TRUE	igd	NA	NA	NA	NA	NA	NA	NA	0.0025554	2.04E-10	Serum 25-Hydroxyvitamin D levels || id:ebi-a-GCST90000618	TRUE	reported	WO8j2s	textfile	2	TRUE
TRUE	igd	NA	NA	NA	NA	NA	NA	NA	0.00205911	2.26E-12	Serum 25-Hydroxyvitamin D levels || id:ebi-a-GCST90000618	TRUE	reported	WO8j2s	textfile	2	TRUE
TRUE	igd	NA	NA	NA	NA	NA	NA	NA	0.00286182	2.39E-19	Serum 25-Hydroxyvitamin D levels || id:ebi-a-GCST90000618	TRUE	reported	WO8j2s	textfile	2	TRUE
TRUE	igd	NA	NA	NA	NA	NA	NA	NA	0.00209882	9.27E-09	Serum 25-Hydroxyvitamin D levels || id:ebi-a-GCST90000618	TRUE	reported	WO8j2s	textfile	2	TRUE
TRUE	igd	NA	NA	NA	NA	NA	NA	NA	0.00340001	2.93E-08	Serum 25-Hydroxyvitamin D levels || id:ebi-a-GCST90000618	TRUE	reported	WO8j2s	textfile	2	TRUE
TRUE	igd	NA	NA	NA	NA	NA	NA	NA	0.00320607	1.04E-08	Serum 25-Hydroxyvitamin D levels || id:ebi-a-GCST90000618	TRUE	reported	WO8j2s	textfile	2	TRUE
TRUE	igd	NA	NA	NA	NA	NA	NA	NA	0.00243165	8.17E-19	Serum 25-Hydroxyvitamin D levels || id:ebi-a-GCST90000618	TRUE	reported	WO8j2s	textfile	2	TRUE
TRUE	igd	NA	NA	NA	NA	NA	NA	NA	0.00225626	1.49E-09	Serum 25-Hydroxyvitamin D levels || id:ebi-a-GCST90000618	TRUE	reported	WO8j2s	textfile	2	TRUE
TRUE	igd	NA	NA	NA	NA	NA	NA	NA	0.0026652	5.15E-10	Serum 25-Hydroxyvitamin D levels || id:ebi-a-GCST90000618	TRUE	reported	WO8j2s	textfile	2	TRUE
TRUE	igd	TRUE	rs7652808	rs1375545	T	G	G	T	0.00212614	1.36E-23	Serum 25-Hydroxyvitamin D levels || id:ebi-a-GCST90000618	TRUE	reported	WO8j2s	textfile	2	TRUE
TRUE	igd	NA	NA	NA	NA	NA	NA	NA	0.00206215	7.05E-09	Serum 25-Hydroxyvitamin D levels || id:ebi-a-GCST90000618	TRUE	reported	WO8j2s	textfile	2	TRUE
TRUE	igd	TRUE	rs7784802	rs10244141	T	A	T	C	0.0021157	3.06E-10	Serum 25-Hydroxyvitamin D levels || id:ebi-a-GCST90000618	TRUE	reported	WO8j2s	textfile	2	TRUE
TRUE	igd	NA	NA	NA	NA	NA	NA	NA	0.00259019	3.94E-09	Serum 25-Hydroxyvitamin D levels || id:ebi-a-GCST90000618	TRUE	reported	WO8j2s	textfile	2	TRUE
TRUE	igd	NA	NA	NA	NA	NA	NA	NA	0.00905963	6.53E-09	Serum 25-Hydroxyvitamin D levels || id:ebi-a-GCST90000618	TRUE	reported	WO8j2s	textfile	2	TRUE
TRUE	igd	NA	NA	NA	NA	NA	NA	NA	0.00332162	8.33E-09	Serum 25-Hydroxyvitamin D levels || id:ebi-a-GCST90000618	TRUE	reported	WO8j2s	textfile	2	TRUE
TRUE	igd	NA	NA	NA	NA	NA	NA	NA	0.00203856	1.48E-10	Serum 25-Hydroxyvitamin D levels || id:ebi-a-GCST90000618	TRUE	reported	WO8j2s	textfile	2	FALSE
TRUE	igd	NA	NA	NA	NA	NA	NA	NA	0.00266074	1.94E-38	Serum 25-Hydroxyvitamin D levels || id:ebi-a-GCST90000618	TRUE	reported	WO8j2s	textfile	2	TRUE
TRUE	igd	NA	NA	NA	NA	NA	NA	NA	0.00205997	1.20E-14	Serum 25-Hydroxyvitamin D levels || id:ebi-a-GCST90000618	TRUE	reported	WO8j2s	textfile	2	TRUE
TRUE	igd	NA	NA	NA	NA	NA	NA	NA	0.00382316	1.36E-20	Serum 25-Hydroxyvitamin D levels || id:ebi-a-GCST90000618	TRUE	reported	WO8j2s	textfile	2	TRUE
TRUE	igd	NA	NA	NA	NA	NA	NA	NA	0.00254898	1.77E-65	Serum 25-Hydroxyvitamin D levels || id:ebi-a-GCST90000618	TRUE	reported	WO8j2s	textfile	2	TRUE
TRUE	igd	NA	NA	NA	NA	NA	NA	NA	0.00205423	1.21E-08	Serum 25-Hydroxyvitamin D levels || id:ebi-a-GCST90000618	TRUE	reported	WO8j2s	textfile	2	TRUE
TRUE	igd	TRUE	rs9409266	rs700059	G	A	G	A	0.00294656	1.24E-08	Serum 25-Hydroxyvitamin D levels || id:ebi-a-GCST90000618	TRUE	reported	WO8j2s	textfile	2	TRUE
TRUE	igd	NA	NA	NA	NA	NA	NA	NA	0.00298936	3.50E-42	Serum 25-Hydroxyvitamin D levels || id:ebi-a-GCST90000618	TRUE	reported	WO8j2s	textfile	2	TRUE
TRUE	igd	NA	NA	NA	NA	NA	NA	NA	0.00224484	4.19E-08	Serum 25-Hydroxyvitamin D levels || id:ebi-a-GCST90000618	TRUE	reported	WO8j2s	textfile	2	TRUE
TRUE	igd	NA	NA	NA	NA	NA	NA	NA	0.00217811	3.51E-09	Serum 25-Hydroxyvitamin D levels || id:ebi-a-GCST90000618	TRUE	reported	WO8j2s	textfile	2	TRUE
TRUE	igd	NA	NA	NA	NA	NA	NA	NA	0.00407655	9.47E-09	Serum 25-Hydroxyvitamin D levels || id:ebi-a-GCST90000618	TRUE	reported	WO8j2s	textfile	2	TRUE
TRUE	igd	NA	NA	NA	NA	NA	NA	NA	0.021	8.08E-15	C3epi25OHD3	TRUE	reported	x0bgYq	textfile	2	TRUE
TRUE	igd	NA	NA	NA	NA	NA	NA	NA	0.017	1.95E-15	C3epi25OHD3	TRUE	reported	x0bgYq	textfile	2	TRUE
TRUE	igd	NA	NA	NA	NA	NA	NA	NA	0.0023507	1.08E-09	Serum 25-Hydroxyvitamin D levels || id:ebi-a-GCST90000618	TRUE	reported	WO8j2s	textfile	2	TRUE
TRUE	igd	NA	NA	NA	NA	NA	NA	NA	0.00205629	2.15E-08	Serum 25-Hydroxyvitamin D levels || id:ebi-a-GCST90000618	TRUE	reported	WO8j2s	textfile	2	TRUE
TRUE	igd	TRUE	rs1042034	rs2678379	C	T	A	G	0.00250017	1.45E-09	Serum 25-Hydroxyvitamin D levels || id:ebi-a-GCST90000618	TRUE	reported	WO8j2s	textfile	2	TRUE
TRUE	igd	NA	NA	NA	NA	NA	NA	NA	0.00264437	7.34E-11	Serum 25-Hydroxyvitamin D levels || id:ebi-a-GCST90000618	TRUE	reported	WO8j2s	textfile	2	TRUE
TRUE	igd	NA	NA	NA	NA	NA	NA	NA	0.00218016	7.96E-10	Serum 25-Hydroxyvitamin D levels || id:ebi-a-GCST90000618	TRUE	reported	WO8j2s	textfile	2	TRUE
TRUE	igd	NA	NA	NA	NA	NA	NA	NA	0.00374376	5.58E-17	Serum 25-Hydroxyvitamin D levels || id:ebi-a-GCST90000618	TRUE	reported	WO8j2s	textfile	2	TRUE
TRUE	igd	TRUE	rs10859995	rs3819817	T	C	C	T	0.00205452	4.60E-100	Serum 25-Hydroxyvitamin D levels || id:ebi-a-GCST90000618	TRUE	reported	WO8j2s	textfile	2	TRUE
TRUE	igd	NA	NA	NA	NA	NA	NA	NA	0.00572507	3.73E-17	Serum 25-Hydroxyvitamin D levels || id:ebi-a-GCST90000618	TRUE	reported	WO8j2s	textfile	2	TRUE
TRUE	igd	NA	NA	NA	NA	NA	NA	NA	0.00266997	9.64E-18	Serum 25-Hydroxyvitamin D levels || id:ebi-a-GCST90000618	TRUE	reported	WO8j2s	textfile	2	TRUE
TRUE	igd	NA	NA	NA	NA	NA	NA	NA	0.00212672	7.14E-23	Serum 25-Hydroxyvitamin D levels || id:ebi-a-GCST90000618	TRUE	reported	WO8j2s	textfile	2	TRUE
TRUE	igd	NA	NA	NA	NA	NA	NA	NA	0.00234081	7.97E-14	Serum 25-Hydroxyvitamin D levels || id:ebi-a-GCST90000618	TRUE	reported	WO8j2s	textfile	2	TRUE
TRUE	igd	NA	NA	NA	NA	NA	NA	NA	0.0020293	6.06E-16	Serum 25-Hydroxyvitamin D levels || id:ebi-a-GCST90000618	TRUE	reported	WO8j2s	textfile	2	TRUE
TRUE	igd	NA	NA	NA	NA	NA	NA	NA	0.00498229	2.36E-56	Serum 25-Hydroxyvitamin D levels || id:ebi-a-GCST90000618	TRUE	reported	WO8j2s	textfile	2	TRUE
TRUE	igd	NA	NA	NA	NA	NA	NA	NA	0.00298265	9.72E-17	Serum 25-Hydroxyvitamin D levels || id:ebi-a-GCST90000618	TRUE	reported	WO8j2s	textfile	2	TRUE
TRUE	igd	NA	NA	NA	NA	NA	NA	NA	0.00225483	3.08E-125	Serum 25-Hydroxyvitamin D levels || id:ebi-a-GCST90000618	TRUE	reported	WO8j2s	textfile	2	TRUE
TRUE	igd	NA	NA	NA	NA	NA	NA	NA	0.00258052	4.85E-08	Serum 25-Hydroxyvitamin D levels || id:ebi-a-GCST90000618	TRUE	reported	WO8j2s	textfile	2	TRUE
TRUE	igd	NA	NA	NA	NA	NA	NA	NA	0.00209454	1.01E-10	Serum 25-Hydroxyvitamin D levels || id:ebi-a-GCST90000618	TRUE	reported	WO8j2s	textfile	2	TRUE
TRUE	igd	NA	NA	NA	NA	NA	NA	NA	0.00206406	2.65E-29	Serum 25-Hydroxyvitamin D levels || id:ebi-a-GCST90000618	TRUE	reported	WO8j2s	textfile	2	TRUE

TRUE	igd	NA	NA	NA	NA	NA	NA	NA	0.0030906	8.16E-09	Serum 25-Hydroxyvitamin D levels || id:ebi-a-GCST90000618	TRUE	reported	WO8j2s	textfile	2	TRUE
TRUE	igd	NA	NA	NA	NA	NA	NA	NA	0.00240614	9.62E-122	Serum 25-Hydroxyvitamin D levels || id:ebi-a-GCST90000618	TRUE	reported	WO8j2s	textfile	2	TRUE
TRUE	igd	NA	NA	NA	NA	NA	NA	NA	0.00267451	2.45E-08	Serum 25-Hydroxyvitamin D levels || id:ebi-a-GCST90000618	TRUE	reported	WO8j2s	textfile	2	TRUE
TRUE	igd	TRUE	rs12462826	rs2607454	A	G	T	G	0.0021149	4.18E-10	Serum 25-Hydroxyvitamin D levels || id:ebi-a-GCST90000618	TRUE	reported	WO8j2s	textfile	2	TRUE
TRUE	igd	NA	NA	NA	NA	NA	NA	NA	0.00206932	1.00E-200	Serum 25-Hydroxyvitamin D levels || id:ebi-a-GCST90000618	TRUE	reported	WO8j2s	textfile	2	TRUE
TRUE	igd	NA	NA	NA	NA	NA	NA	NA	0.00207421	1.96E-21	Serum 25-Hydroxyvitamin D levels || id:ebi-a-GCST90000618	TRUE	reported	WO8j2s	textfile	2	TRUE
TRUE	igd	TRUE	rs12775091	rs34408087	T	C	A	G	0.00247693	3.33E-10	Serum 25-Hydroxyvitamin D levels || id:ebi-a-GCST90000618	TRUE	reported	WO8j2s	textfile	2	TRUE
TRUE	igd	NA	NA	NA	NA	NA	NA	NA	0.00451036	2.78E-08	Serum 25-Hydroxyvitamin D levels || id:ebi-a-GCST90000618	TRUE	reported	WO8j2s	textfile	2	TRUE
TRUE	igd	NA	NA	NA	NA	NA	NA	NA	0.00208616	4.63E-09	Serum 25-Hydroxyvitamin D levels || id:ebi-a-GCST90000618	TRUE	reported	WO8j2s	textfile	2	TRUE
TRUE	igd	NA	NA	NA	NA	NA	NA	NA	0.00336578	4.36E-11	Serum 25-Hydroxyvitamin D levels || id:ebi-a-GCST90000618	TRUE	reported	WO8j2s	textfile	2	TRUE
TRUE	igd	NA	NA	NA	NA	NA	NA	NA	0.00205837	1.02E-09	Serum 25-Hydroxyvitamin D levels || id:ebi-a-GCST90000618	TRUE	reported	WO8j2s	textfile	2	TRUE
TRUE	igd	NA	NA	NA	NA	NA	NA	NA	0.00245028	1.62E-13	Serum 25-Hydroxyvitamin D levels || id:ebi-a-GCST90000618	TRUE	reported	WO8j2s	textfile	2	TRUE
TRUE	igd	NA	NA	NA	NA	NA	NA	NA	0.00215115	1.56E-10	Serum 25-Hydroxyvitamin D levels || id:ebi-a-GCST90000618	TRUE	reported	WO8j2s	textfile	2	TRUE
TRUE	igd	NA	NA	NA	NA	NA	NA	NA	0.00299675	1.82E-08	Serum 25-Hydroxyvitamin D levels || id:ebi-a-GCST90000618	TRUE	reported	WO8j2s	textfile	2	TRUE
TRUE	igd	NA	NA	NA	NA	NA	NA	NA	0.00559561	3.01E-08	Serum 25-Hydroxyvitamin D levels || id:ebi-a-GCST90000618	TRUE	reported	WO8j2s	textfile	2	TRUE
TRUE	igd	NA	NA	NA	NA	NA	NA	NA	0.00323446	4.43E-16	Serum 25-Hydroxyvitamin D levels || id:ebi-a-GCST90000618	TRUE	reported	WO8j2s	textfile	2	TRUE
TRUE	igd	NA	NA	NA	NA	NA	NA	NA	0.00421754	1.52E-16	Serum 25-Hydroxyvitamin D levels || id:ebi-a-GCST90000618	TRUE	reported	WO8j2s	textfile	2	TRUE
TRUE	igd	NA	NA	NA	NA	NA	NA	NA	0.00566106	8.49E-18	Serum 25-Hydroxyvitamin D levels || id:ebi-a-GCST90000618	TRUE	reported	WO8j2s	textfile	2	TRUE
TRUE	igd	NA	NA	NA	NA	NA	NA	NA	0.00221719	1.59E-08	Serum 25-Hydroxyvitamin D levels || id:ebi-a-GCST90000618	TRUE	reported	WO8j2s	textfile	2	TRUE
TRUE	igd	NA	NA	NA	NA	NA	NA	NA	0.0021713	5.14E-10	Serum 25-Hydroxyvitamin D levels || id:ebi-a-GCST90000618	TRUE	reported	WO8j2s	textfile	2	TRUE
TRUE	igd	NA	NA	NA	NA	NA	NA	NA	0.00780044	4.59E-15	Serum 25-Hydroxyvitamin D levels || id:ebi-a-GCST90000618	TRUE	reported	WO8j2s	textfile	2	TRUE
TRUE	igd	TRUE	rs1800588	rs2070895	T	C	A	G	0.00246932	4.73E-35	Serum 25-Hydroxyvitamin D levels || id:ebi-a-GCST90000618	TRUE	reported	WO8j2s	textfile	2	TRUE
TRUE	igd	TRUE	rs1841850	rs8123293	C	A	G	A	0.00316491	6.73E-22	Serum 25-Hydroxyvitamin D levels || id:ebi-a-GCST90000618	TRUE	reported	WO8j2s	textfile	2	TRUE
TRUE	igd	NA	NA	NA	NA	NA	NA	NA	0.00203065	3.49E-11	Serum 25-Hydroxyvitamin D levels || id:ebi-a-GCST90000618	TRUE	reported	WO8j2s	textfile	2	TRUE
TRUE	igd	NA	NA	NA	NA	NA	NA	NA	0.00282689	5.72E-13	Serum 25-Hydroxyvitamin D levels || id:ebi-a-GCST90000618	TRUE	reported	WO8j2s	textfile	2	TRUE
TRUE	igd	NA	NA	NA	NA	NA	NA	NA	0.00208637	4.45E-08	Serum 25-Hydroxyvitamin D levels || id:ebi-a-GCST90000618	TRUE	reported	WO8j2s	textfile	2	TRUE
TRUE	igd	NA	NA	NA	NA	NA	NA	NA	0.00272732	9.41E-11	Serum 25-Hydroxyvitamin D levels || id:ebi-a-GCST90000618	TRUE	reported	WO8j2s	textfile	2	TRUE
TRUE	igd	NA	NA	NA	NA	NA	NA	NA	0.00412	1.22E-12	Serum 25-Hydroxyvitamin D levels || id:ebi-a-GCST90000618	TRUE	reported	WO8j2s	textfile	2	TRUE
TRUE	igd	TRUE	rs2171427	rs11047561	A	G	G	C	0.00281738	4.26E-09	Serum 25-Hydroxyvitamin D levels || id:ebi-a-GCST90000618	TRUE	reported	WO8j2s	textfile	2	TRUE
TRUE	igd	NA	NA	NA	NA	NA	NA	NA	0.00331409	4.75E-14	Serum 25-Hydroxyvitamin D levels || id:ebi-a-GCST90000618	TRUE	reported	WO8j2s	textfile	2	TRUE
TRUE	igd	NA	NA	NA	NA	NA	NA	NA	0.00273999	7.80E-15	Serum 25-Hydroxyvitamin D levels || id:ebi-a-GCST90000618	TRUE	reported	WO8j2s	textfile	2	TRUE
TRUE	igd	NA	NA	NA	NA	NA	NA	NA	0.00225578	1.57E-08	Serum 25-Hydroxyvitamin D levels || id:ebi-a-GCST90000618	TRUE	reported	WO8j2s	textfile	2	TRUE
TRUE	igd	TRUE	rs2398113	rs17147117	G	A	C	A	0.00205797	1.10E-08	Serum 25-Hydroxyvitamin D levels || id:ebi-a-GCST90000618	TRUE	reported	WO8j2s	textfile	2	TRUE
TRUE	igd	NA	NA	NA	NA	NA	NA	NA	0.00267333	2.80E-08	Serum 25-Hydroxyvitamin D levels || id:ebi-a-GCST90000618	TRUE	reported	WO8j2s	textfile	2	TRUE
TRUE	igd	NA	NA	NA	NA	NA	NA	NA	0.00520826	2.98E-79	Serum 25-Hydroxyvitamin D levels || id:ebi-a-GCST90000618	TRUE	reported	WO8j2s	textfile	2	TRUE
TRUE	igd	NA	NA	NA	NA	NA	NA	NA	0.00209685	4.97E-09	Serum 25-Hydroxyvitamin D levels || id:ebi-a-GCST90000618	TRUE	reported	WO8j2s	textfile	2	TRUE
TRUE	igd	NA	NA	NA	NA	NA	NA	NA	0.00203474	1.23E-08	Serum 25-Hydroxyvitamin D levels || id:ebi-a-GCST90000618	TRUE	reported	WO8j2s	textfile	2	TRUE
TRUE	igd	NA	NA	NA	NA	NA	NA	NA	0.0021104	8.71E-09	Serum 25-Hydroxyvitamin D levels || id:ebi-a-GCST90000618	TRUE	reported	WO8j2s	textfile	2	TRUE
TRUE	igd	NA	NA	NA	NA	NA	NA	NA	0.00218678	5.66E-12	Serum 25-Hydroxyvitamin D levels || id:ebi-a-GCST90000618	TRUE	reported	WO8j2s	textfile	2	TRUE
TRUE	igd	TRUE	rs28435470	rs80059826	G	A	T	C	0.00214831	3.29E-08	Serum 25-Hydroxyvitamin D levels || id:ebi-a-GCST90000618	TRUE	reported	WO8j2s	textfile	2	TRUE
TRUE	igd	NA	NA	NA	NA	NA	NA	NA	0.00308665	2.77E-13	Serum 25-Hydroxyvitamin D levels || id:ebi-a-GCST90000618	TRUE	reported	WO8j2s	textfile	2	TRUE
TRUE	igd	NA	NA	NA	NA	NA	NA	NA	0.00216338	1.41E-09	Serum 25-Hydroxyvitamin D levels || id:ebi-a-GCST90000618	TRUE	reported	WO8j2s	textfile	2	TRUE
TRUE	igd	NA	NA	NA	NA	NA	NA	NA	0.00298008	1.00E-13	Serum 25-Hydroxyvitamin D levels || id:ebi-a-GCST90000618	TRUE	reported	WO8j2s	textfile	2	TRUE
TRUE	igd	NA	NA	NA	NA	NA	NA	NA	0.00227676	2.03E-09	Serum 25-Hydroxyvitamin D levels || id:ebi-a-GCST90000618	TRUE	reported	WO8j2s	textfile	2	TRUE
TRUE	igd	NA	NA	NA	NA	NA	NA	NA	0.00231839	1.33E-11	Serum 25-Hydroxyvitamin D levels || id:ebi-a-GCST90000618	TRUE	reported	WO8j2s	textfile	2	TRUE
TRUE	igd	NA	NA	NA	NA	NA	NA	NA	0.00234858	2.42E-09	Serum 25-Hydroxyvitamin D levels || id:ebi-a-GCST90000618	TRUE	reported	WO8j2s	textfile	2	TRUE
TRUE	igd	NA	NA	NA	NA	NA	NA	NA	0.0026815	5.08E-09	Serum 25-Hydroxyvitamin D levels || id:ebi-a-GCST90000618	TRUE	reported	WO8j2s	textfile	2	TRUE
TRUE	igd	NA	NA	NA	NA	NA	NA	NA	0.00214072	1.65E-27	Serum 25-Hydroxyvitamin D levels || id:ebi-a-GCST90000618	TRUE	reported	WO8j2s	textfile	2	TRUE
TRUE	igd	NA	NA	NA	NA	NA	NA	NA	0.00363266	1.31E-39	Serum 25-Hydroxyvitamin D levels || id:ebi-a-GCST90000618	TRUE	reported	WO8j2s	textfile	2	TRUE
TRUE	igd	NA	NA	NA	NA	NA	NA	NA	0.00298056	1.00E-200	Serum 25-Hydroxyvitamin D levels || id:ebi-a-GCST90000618	TRUE	reported	WO8j2s	textfile	2	TRUE
TRUE	igd	NA	NA	NA	NA	NA	NA	NA	0.00249007	2.76E-09	Serum 25-Hydroxyvitamin D levels || id:ebi-a-GCST90000618	TRUE	reported	WO8j2s	textfile	2	TRUE
TRUE	igd	NA	NA	NA	NA	NA	NA	NA	0.00216263	6.62E-33	Serum 25-Hydroxyvitamin D levels || id:ebi-a-GCST90000618	TRUE	reported	WO8j2s	textfile	2	TRUE
TRUE	igd	NA	NA	NA	NA	NA	NA	NA	0.00256676	1.86E-11	Serum 25-Hydroxyvitamin D levels || id:ebi-a-GCST90000618	TRUE	reported	WO8j2s	textfile	2	TRUE
TRUE	igd	NA	NA	NA	NA	NA	NA	NA	0.00265905	3.95E-13	Serum 25-Hydroxyvitamin D levels || id:ebi-a-GCST90000618	TRUE	reported	WO8j2s	textfile	2	TRUE
TRUE	igd	NA	NA	NA	NA	NA	NA	NA	0.00225079	1.68E-09	Serum 25-Hydroxyvitamin D levels || id:ebi-a-GCST90000618	TRUE	reported	WO8j2s	textfile	2	TRUE
TRUE	igd	NA	NA	NA	NA	NA	NA	NA	0.00204286	2.23E-09	Serum 25-Hydroxyvitamin D levels || id:ebi-a-GCST90000618	TRUE	reported	WO8j2s	textfile	2	TRUE
TRUE	igd	TRUE	rs57601828	rs8181776	T	A	C	A	0.00208192	2.96E-08	Serum 25-Hydroxyvitamin D levels || id:ebi-a-GCST90000618	TRUE	reported	WO8j2s	textfile	2	TRUE
TRUE	igd	NA	NA	NA	NA	NA	NA	NA	0.00221143	1.74E-09	Serum 25-Hydroxyvitamin D levels || id:ebi-a-GCST90000618	TRUE	reported	WO8j2s	textfile	2	TRUE
TRUE	igd	NA	NA	NA	NA	NA	NA	NA	0.00210628	2.44E-11	Serum 25-Hydroxyvitamin D levels || id:ebi-a-GCST90000618	TRUE	reported	WO8j2s	textfile	2	TRUE
TRUE	igd	NA	NA	NA	NA	NA	NA	NA	0.0020505	2.25E-08	Serum 25-Hydroxyvitamin D levels || id:ebi-a-GCST90000618	TRUE	reported	WO8j2s	textfile	2	TRUE
TRUE	igd	NA	NA	NA	NA	NA	NA	NA	0.00658876	1.16E-35	Serum 25-Hydroxyvitamin D levels || id:ebi-a-GCST90000618	TRUE	reported	WO8j2s	textfile	2	TRUE
TRUE	igd	NA	NA	NA	NA	NA	NA	NA	0.00597754	8.05E-10	Serum 25-Hydroxyvitamin D levels || id:ebi-a-GCST90000618	TRUE	reported	WO8j2s	textfile	2	TRUE
TRUE	igd	NA	NA	NA	NA	NA	NA	NA	0.00224413	3.12E-08	Serum 25-Hydroxyvitamin D levels || id:ebi-a-GCST90000618	TRUE	reported	WO8j2s	textfile	2	TRUE
TRUE	igd	NA	NA	NA	NA	NA	NA	NA	0.00276373	1.60E-108	Serum 25-Hydroxyvitamin D levels || id:ebi-a-GCST90000618	TRUE	reported	WO8j2s	textfile	2	TRUE
TRUE	igd	NA	NA	NA	NA	NA	NA	NA	0.0026042	7.55E-09	Serum 25-Hydroxyvitamin D levels || id:ebi-a-GCST90000618	TRUE	reported	WO8j2s	textfile	2	TRUE
TRUE	igd	NA	NA	NA	NA	NA	NA	NA	0.00234031	1.27E-10	Serum 25-Hydroxyvitamin D levels || id:ebi-a-GCST90000618	TRUE	reported	WO8j2s	textfile	2	TRUE
TRUE	igd	NA	NA	NA	NA	NA	NA	NA	0.0025554	2.04E-10	Serum 25-Hydroxyvitamin D levels || id:ebi-a-GCST90000618	TRUE	reported	WO8j2s	textfile	2	TRUE
TRUE	igd	NA	NA	NA	NA	NA	NA	NA	0.00205911	2.26E-12	Serum 25-Hydroxyvitamin D levels || id:ebi-a-GCST90000618	TRUE	reported	WO8j2s	textfile	2	TRUE
TRUE	igd	NA	NA	NA	NA	NA	NA	NA	0.00286182	2.39E-19	Serum 25-Hydroxyvitamin D levels || id:ebi-a-GCST90000618	TRUE	reported	WO8j2s	textfile	2	TRUE
TRUE	igd	NA	NA	NA	NA	NA	NA	NA	0.00209882	9.27E-09	Serum 25-Hydroxyvitamin D levels || id:ebi-a-GCST90000618	TRUE	reported	WO8j2s	textfile	2	TRUE
TRUE	igd	NA	NA	NA	NA	NA	NA	NA	0.00340001	2.93E-08	Serum 25-Hydroxyvitamin D levels || id:ebi-a-GCST90000618	TRUE	reported	WO8j2s	textfile	2	TRUE
TRUE	igd	NA	NA	NA	NA	NA	NA	NA	0.00388928	9.15E-09	Serum 25-Hydroxyvitamin D levels || id:ebi-a-GCST90000618	TRUE	reported	WO8j2s	textfile	2	TRUE
TRUE	igd	NA	NA	NA	NA	NA	NA	NA	0.00320607	1.04E-08	Serum 25-Hydroxyvitamin D levels || id:ebi-a-GCST90000618	TRUE	reported	WO8j2s	textfile	2	TRUE
TRUE	igd	NA	NA	NA	NA	NA	NA	NA	0.00243165	8.17E-19	Serum 25-Hydroxyvitamin D levels || id:ebi-a-GCST90000618	TRUE	reported	WO8j2s	textfile	2	TRUE
TRUE	igd	NA	NA	NA	NA	NA	NA	NA	0.00225626	1.49E-09	Serum 25-Hydroxyvitamin D levels || id:ebi-a-GCST90000618	TRUE	reported	WO8j2s	textfile	2	TRUE
TRUE	igd	NA	NA	NA	NA	NA	NA	NA	0.0026652	5.15E-10	Serum 25-Hydroxyvitamin D levels || id:ebi-a-GCST90000618	TRUE	reported	WO8j2s	textfile	2	TRUE
TRUE	igd	TRUE	rs7652808	rs1375544	T	G	A	G	0.00212614	1.36E-23	Serum 25-Hydroxyvitamin D levels || id:ebi-a-GCST90000618	TRUE	reported	WO8j2s	textfile	2	TRUE
TRUE	igd	NA	NA	NA	NA	NA	NA	NA	0.00206215	7.05E-09	Serum 25-Hydroxyvitamin D levels || id:ebi-a-GCST90000618	TRUE	reported	WO8j2s	textfile	2	TRUE
TRUE	igd	TRUE	rs7784802	rs10244141	T	A	T	C	0.0021157	3.06E-10	Serum 25-Hydroxyvitamin D levels || id:ebi-a-GCST90000618	TRUE	reported	WO8j2s	textfile	2	TRUE
TRUE	igd	NA	NA	NA	NA	NA	NA	NA	0.00259019	3.94E-09	Serum 25-Hydroxyvitamin D levels || id:ebi-a-GCST90000618	TRUE	reported	WO8j2s	textfile	2	TRUE
TRUE	igd	NA	NA	NA	NA	NA	NA	NA	0.00905963	6.53E-09	Serum 25-Hydroxyvitamin D levels || id:ebi-a-GCST90000618	TRUE	reported	WO8j2s	textfile	2	TRUE
TRUE	igd	NA	NA	NA	NA	NA	NA	NA	0.00332162	8.33E-09	Serum 25-Hydroxyvitamin D levels || id:ebi-a-GCST90000618	TRUE	reported	WO8j2s	textfile	2	TRUE
TRUE	igd	NA	NA	NA	NA	NA	NA	NA	0.00203856	1.48E-10	Serum 25-Hydroxyvitamin D levels || id:ebi-a-GCST90000618	TRUE	reported	WO8j2s	textfile	2	FALSE
TRUE	igd	NA	NA	NA	NA	NA	NA	NA	0.00266074	1.94E-38	Serum 25-Hydroxyvitamin D levels || id:ebi-a-GCST90000618	TRUE	reported	WO8j2s	textfile	2	TRUE
TRUE	igd	NA	NA	NA	NA	NA	NA	NA	0.00205997	1.20E-14	Serum 25-Hydroxyvitamin D levels || id:ebi-a-GCST90000618	TRUE	reported	WO8j2s	textfile	2	TRUE
TRUE	igd	NA	NA	NA	NA	NA	NA	NA	0.00382316	1.36E-20	Serum 25-Hydroxyvitamin D levels || id:ebi-a-GCST90000618	TRUE	reported	WO8j2s	textfile	2	TRUE
TRUE	igd	NA	NA	NA	NA	NA	NA	NA	0.00254898	1.77E-65	Serum 25-Hydroxyvitamin D levels || id:ebi-a-GCST90000618	TRUE	reported	WO8j2s	textfile	2	TRUE
TRUE	igd	NA	NA	NA	NA	NA	NA	NA	0.00205423	1.21E-08	Serum 25-Hydroxyvitamin D levels || id:ebi-a-GCST90000618	TRUE	reported	WO8j2s	textfile	2	TRUE
TRUE	igd	TRUE	rs9409266	rs700059	G	A	G	A	0.00294656	1.24E-08	Serum 25-Hydroxyvitamin D levels || id:ebi-a-GCST90000618	TRUE	reported	WO8j2s	textfile	2	TRUE
TRUE	igd	NA	NA	NA	NA	NA	NA	NA	0.00298936	3.50E-42	Serum 25-Hydroxyvitamin D levels || id:ebi-a-GCST90000618	TRUE	reported	WO8j2s	textfile	2	TRUE
TRUE	igd	NA	NA	NA	NA	NA	NA	NA	0.00224484	4.19E-08	Serum 25-Hydroxyvitamin D levels || id:ebi-a-GCST90000618	TRUE	reported	WO8j2s	textfile	2	TRUE
TRUE	igd	NA	NA	NA	NA	NA	NA	NA	0.00217811	3.51E-09	Serum 25-Hydroxyvitamin D levels || id:ebi-a-GCST90000618	TRUE	reported	WO8j2s	textfile	2	TRUE
TRUE	igd	NA	NA	NA	NA	NA	NA	NA	0.00407655	9.47E-09	Serum 25-Hydroxyvitamin D levels || id:ebi-a-GCST90000618	TRUE	reported	WO8j2s	textfile	2	TRUE
TRUE	igd	NA	NA	NA	NA	NA	NA	NA	0.023	3.22E-60	25OHD3	TRUE	reported	20duX4	textfile	2	TRUE
TRUE	igd	NA	NA	NA	NA	NA	NA	NA	0.01	1.94E-08	25OHD3	TRUE	reported	20duX4	textfile	2	TRUE
TRUE	igd	NA	NA	NA	NA	NA	NA	NA	0.009	1.09E-15	25OHD3	TRUE	reported	20duX4	textfile	2	TRUE
TRUE	igd	NA	NA	NA	NA	NA	NA	NA	0.008	4.08E-59	25OHD3	TRUE	reported	20duX4	textfile	2	TRUE
TRUE	igd	NA	NA	NA	NA	NA	NA	NA	0.007	3.59E-16	25OHD3	TRUE	reported	20duX4	textfile	2	TRUE
TRUE	igd	NA	NA	NA	NA	NA	NA	NA	0.01	1.27E-08	25OHD3	TRUE	reported	20duX4	textfile	2	TRUE
TRUE	igd	NA	NA	NA	NA	NA	NA	NA	0.021	8.08E-15	C3epi25OHD3	TRUE	reported	x0bgYq	textfile	2	TRUE
TRUE	igd	NA	NA	NA	NA	NA	NA	NA	0.017	1.95E-15	C3epi25OHD3	TRUE	reported	x0bgYq	textfile	2	TRUE
TRUE	igd	NA	NA	NA	NA	NA	NA	NA	0.0023507	1.08E-09	Serum 25-Hydroxyvitamin D levels || id:ebi-a-GCST90000618	TRUE	reported	WO8j2s	textfile	2	TRUE
TRUE	igd	NA	NA	NA	NA	NA	NA	NA	0.00205629	2.15E-08	Serum 25-Hydroxyvitamin D levels || id:ebi-a-GCST90000618	TRUE	reported	WO8j2s	textfile	2	TRUE
TRUE	igd	TRUE	rs1042034	rs2678379	C	T	A	G	0.00250017	1.45E-09	Serum 25-Hydroxyvitamin D levels || id:ebi-a-GCST90000618	TRUE	reported	WO8j2s	textfile	2	TRUE
TRUE	igd	NA	NA	NA	NA	NA	NA	NA	0.00264437	7.34E-11	Serum 25-Hydroxyvitamin D levels || id:ebi-a-GCST90000618	TRUE	reported	WO8j2s	textfile	2	TRUE
TRUE	igd	NA	NA	NA	NA	NA	NA	NA	0.00218016	7.96E-10	Serum 25-Hydroxyvitamin D levels || id:ebi-a-GCST90000618	TRUE	reported	WO8j2s	textfile	2	TRUE
TRUE	igd	NA	NA	NA	NA	NA	NA	NA	0.00374376	5.58E-17	Serum 25-Hydroxyvitamin D levels || id:ebi-a-GCST90000618	TRUE	reported	WO8j2s	textfile	2	TRUE
TRUE	igd	TRUE	rs10859995	rs3819817	T	C	C	T	0.00205452	4.60E-100	Serum 25-Hydroxyvitamin D levels || id:ebi-a-GCST90000618	TRUE	reported	WO8j2s	textfile	2	TRUE
TRUE	igd	NA	NA	NA	NA	NA	NA	NA	0.00572507	3.73E-17	Serum 25-Hydroxyvitamin D levels || id:ebi-a-GCST90000618	TRUE	reported	WO8j2s	textfile	2	TRUE
TRUE	igd	NA	NA	NA	NA	NA	NA	NA	0.00266997	9.64E-18	Serum 25-Hydroxyvitamin D levels || id:ebi-a-GCST90000618	TRUE	reported	WO8j2s	textfile	2	TRUE
TRUE	igd	NA	NA	NA	NA	NA	NA	NA	0.00212672	7.14E-23	Serum 25-Hydroxyvitamin D levels || id:ebi-a-GCST90000618	TRUE	reported	WO8j2s	textfile	2	TRUE
TRUE	igd	NA	NA	NA	NA	NA	NA	NA	0.00234081	7.97E-14	Serum 25-Hydroxyvitamin D levels || id:ebi-a-GCST90000618	TRUE	reported	WO8j2s	textfile	2	TRUE
TRUE	igd	NA	NA	NA	NA	NA	NA	NA	0.0020293	6.06E-16	Serum 25-Hydroxyvitamin D levels || id:ebi-a-GCST90000618	TRUE	reported	WO8j2s	textfile	2	TRUE
TRUE	igd	NA	NA	NA	NA	NA	NA	NA	0.00498229	2.36E-56	Serum 25-Hydroxyvitamin D levels || id:ebi-a-GCST90000618	TRUE	reported	WO8j2s	textfile	2	TRUE
TRUE	igd	NA	NA	NA	NA	NA	NA	NA	0.00298265	9.72E-17	Serum 25-Hydroxyvitamin D levels || id:ebi-a-GCST90000618	TRUE	reported	WO8j2s	textfile	2	TRUE
TRUE	igd	NA	NA	NA	NA	NA	NA	NA	0.00225483	3.08E-125	Serum 25-Hydroxyvitamin D levels || id:ebi-a-GCST90000618	TRUE	reported	WO8j2s	textfile	2	TRUE
TRUE	igd	NA	NA	NA	NA	NA	NA	NA	0.00258052	4.85E-08	Serum 25-Hydroxyvitamin D levels || id:ebi-a-GCST90000618	TRUE	reported	WO8j2s	textfile	2	TRUE
TRUE	igd	NA	NA	NA	NA	NA	NA	NA	0.00209454	1.01E-10	Serum 25-Hydroxyvitamin D levels || id:ebi-a-GCST90000618	TRUE	reported	WO8j2s	textfile	2	TRUE
TRUE	igd	NA	NA	NA	NA	NA	NA	NA	0.00206406	2.65E-29	Serum 25-Hydroxyvitamin D levels || id:ebi-a-GCST90000618	TRUE	reported	WO8j2s	textfile	2	TRUE
TRUE	igd	NA	NA	NA	NA	NA	NA	NA	0.0030906	8.16E-09	Serum 25-Hydroxyvitamin D levels || id:ebi-a-GCST90000618	TRUE	reported	WO8j2s	textfile	2	TRUE
TRUE	igd	NA	NA	NA	NA	NA	NA	NA	0.00240614	9.62E-122	Serum 25-Hydroxyvitamin D levels || id:ebi-a-GCST90000618	TRUE	reported	WO8j2s	textfile	2	TRUE
TRUE	igd	NA	NA	NA	NA	NA	NA	NA	0.00267451	2.45E-08	Serum 25-Hydroxyvitamin D levels || id:ebi-a-GCST90000618	TRUE	reported	WO8j2s	textfile	2	TRUE
TRUE	igd	TRUE	rs12462826	rs2607454	A	G	T	G	0.0021149	4.18E-10	Serum 25-Hydroxyvitamin D levels || id:ebi-a-GCST90000618	TRUE	reported	WO8j2s	textfile	2	TRUE
TRUE	igd	NA	NA	NA	NA	NA	NA	NA	0.00206932	1.00E-200	Serum 25-Hydroxyvitamin D levels || id:ebi-a-GCST90000618	TRUE	reported	WO8j2s	textfile	2	TRUE
TRUE	igd	NA	NA	NA	NA	NA	NA	NA	0.00207421	1.96E-21	Serum 25-Hydroxyvitamin D levels || id:ebi-a-GCST90000618	TRUE	reported	WO8j2s	textfile	2	TRUE
TRUE	igd	TRUE	rs12775091	rs34408087	T	C	A	G	0.00247693	3.33E-10	Serum 25-Hydroxyvitamin D levels || id:ebi-a-GCST90000618	TRUE	reported	WO8j2s	textfile	2	TRUE
TRUE	igd	NA	NA	NA	NA	NA	NA	NA	0.00451036	2.78E-08	Serum 25-Hydroxyvitamin D levels || id:ebi-a-GCST90000618	TRUE	reported	WO8j2s	textfile	2	TRUE
TRUE	igd	NA	NA	NA	NA	NA	NA	NA	0.00208616	4.63E-09	Serum 25-Hydroxyvitamin D levels || id:ebi-a-GCST90000618	TRUE	reported	WO8j2s	textfile	2	TRUE
TRUE	igd	NA	NA	NA	NA	NA	NA	NA	0.00336578	4.36E-11	Serum 25-Hydroxyvitamin D levels || id:ebi-a-GCST90000618	TRUE	reported	WO8j2s	textfile	2	TRUE
TRUE	igd	NA	NA	NA	NA	NA	NA	NA	0.00205837	1.02E-09	Serum 25-Hydroxyvitamin D levels || id:ebi-a-GCST90000618	TRUE	reported	WO8j2s	textfile	2	TRUE
TRUE	igd	NA	NA	NA	NA	NA	NA	NA	0.00245028	1.62E-13	Serum 25-Hydroxyvitamin D levels || id:ebi-a-GCST90000618	TRUE	reported	WO8j2s	textfile	2	TRUE
TRUE	igd	NA	NA	NA	NA	NA	NA	NA	0.00215115	1.56E-10	Serum 25-Hydroxyvitamin D levels || id:ebi-a-GCST90000618	TRUE	reported	WO8j2s	textfile	2	TRUE
TRUE	igd	NA	NA	NA	NA	NA	NA	NA	0.00299675	1.82E-08	Serum 25-Hydroxyvitamin D levels || id:ebi-a-GCST90000618	TRUE	reported	WO8j2s	textfile	2	TRUE
TRUE	igd	NA	NA	NA	NA	NA	NA	NA	0.00559561	3.01E-08	Serum 25-Hydroxyvitamin D levels || id:ebi-a-GCST90000618	TRUE	reported	WO8j2s	textfile	2	TRUE
TRUE	igd	NA	NA	NA	NA	NA	NA	NA	0.00323446	4.43E-16	Serum 25-Hydroxyvitamin D levels || id:ebi-a-GCST90000618	TRUE	reported	WO8j2s	textfile	2	TRUE
TRUE	igd	NA	NA	NA	NA	NA	NA	NA	0.00421754	1.52E-16	Serum 25-Hydroxyvitamin D levels || id:ebi-a-GCST90000618	TRUE	reported	WO8j2s	textfile	2	TRUE
TRUE	igd	NA	NA	NA	NA	NA	NA	NA	0.00566106	8.49E-18	Serum 25-Hydroxyvitamin D levels || id:ebi-a-GCST90000618	TRUE	reported	WO8j2s	textfile	2	TRUE
TRUE	igd	NA	NA	NA	NA	NA	NA	NA	0.00221719	1.59E-08	Serum 25-Hydroxyvitamin D levels || id:ebi-a-GCST90000618	TRUE	reported	WO8j2s	textfile	2	TRUE
TRUE	igd	NA	NA	NA	NA	NA	NA	NA	0.0021713	5.14E-10	Serum 25-Hydroxyvitamin D levels || id:ebi-a-GCST90000618	TRUE	reported	WO8j2s	textfile	2	TRUE

TRUE	igd	NA	NA	NA	NA	NA	NA	NA	0.00780044	4.59E-15	Serum 25-Hydroxyvitamin D levels || id:ebi-a-GCST90000618	TRUE	reported	WO8j2s	textfile	2	TRUE
TRUE	igd	TRUE	rs1800588	rs588136	T	C	C	T	0.00246932	4.73E-35	Serum 25-Hydroxyvitamin D levels || id:ebi-a-GCST90000618	TRUE	reported	WO8j2s	textfile	2	TRUE
TRUE	igd	TRUE	rs1841850	rs8123293	C	A	G	A	0.00316491	6.73E-22	Serum 25-Hydroxyvitamin D levels || id:ebi-a-GCST90000618	TRUE	reported	WO8j2s	textfile	2	TRUE
TRUE	igd	NA	NA	NA	NA	NA	NA	NA	0.00203065	3.49E-11	Serum 25-Hydroxyvitamin D levels || id:ebi-a-GCST90000618	TRUE	reported	WO8j2s	textfile	2	TRUE
TRUE	igd	NA	NA	NA	NA	NA	NA	NA	0.00282689	5.72E-13	Serum 25-Hydroxyvitamin D levels || id:ebi-a-GCST90000618	TRUE	reported	WO8j2s	textfile	2	TRUE
TRUE	igd	NA	NA	NA	NA	NA	NA	NA	0.00208637	4.45E-08	Serum 25-Hydroxyvitamin D levels || id:ebi-a-GCST90000618	TRUE	reported	WO8j2s	textfile	2	TRUE
TRUE	igd	NA	NA	NA	NA	NA	NA	NA	0.00272732	9.41E-11	Serum 25-Hydroxyvitamin D levels || id:ebi-a-GCST90000618	TRUE	reported	WO8j2s	textfile	2	TRUE
TRUE	igd	NA	NA	NA	NA	NA	NA	NA	0.00412	1.22E-12	Serum 25-Hydroxyvitamin D levels || id:ebi-a-GCST90000618	TRUE	reported	WO8j2s	textfile	2	TRUE
TRUE	igd	TRUE	rs2171427	rs11047561	A	G	G	C	0.00281738	4.26E-09	Serum 25-Hydroxyvitamin D levels || id:ebi-a-GCST90000618	TRUE	reported	WO8j2s	textfile	2	TRUE
TRUE	igd	NA	NA	NA	NA	NA	NA	NA	0.00331409	4.75E-14	Serum 25-Hydroxyvitamin D levels || id:ebi-a-GCST90000618	TRUE	reported	WO8j2s	textfile	2	TRUE
TRUE	igd	NA	NA	NA	NA	NA	NA	NA	0.00273999	7.80E-15	Serum 25-Hydroxyvitamin D levels || id:ebi-a-GCST90000618	TRUE	reported	WO8j2s	textfile	2	TRUE
TRUE	igd	NA	NA	NA	NA	NA	NA	NA	0.00225578	1.57E-08	Serum 25-Hydroxyvitamin D levels || id:ebi-a-GCST90000618	TRUE	reported	WO8j2s	textfile	2	TRUE
TRUE	igd	TRUE	rs2398113	rs17147117	G	A	C	A	0.00205797	1.10E-08	Serum 25-Hydroxyvitamin D levels || id:ebi-a-GCST90000618	TRUE	reported	WO8j2s	textfile	2	TRUE
TRUE	igd	NA	NA	NA	NA	NA	NA	NA	0.00267333	2.80E-08	Serum 25-Hydroxyvitamin D levels || id:ebi-a-GCST90000618	TRUE	reported	WO8j2s	textfile	2	TRUE
TRUE	igd	NA	NA	NA	NA	NA	NA	NA	0.00520826	2.98E-79	Serum 25-Hydroxyvitamin D levels || id:ebi-a-GCST90000618	TRUE	reported	WO8j2s	textfile	2	TRUE
TRUE	igd	NA	NA	NA	NA	NA	NA	NA	0.00209685	4.97E-09	Serum 25-Hydroxyvitamin D levels || id:ebi-a-GCST90000618	TRUE	reported	WO8j2s	textfile	2	TRUE
TRUE	igd	NA	NA	NA	NA	NA	NA	NA	0.00203474	1.23E-08	Serum 25-Hydroxyvitamin D levels || id:ebi-a-GCST90000618	TRUE	reported	WO8j2s	textfile	2	TRUE
TRUE	igd	NA	NA	NA	NA	NA	NA	NA	0.0021104	8.71E-09	Serum 25-Hydroxyvitamin D levels || id:ebi-a-GCST90000618	TRUE	reported	WO8j2s	textfile	2	TRUE
TRUE	igd	NA	NA	NA	NA	NA	NA	NA	0.00218678	5.66E-12	Serum 25-Hydroxyvitamin D levels || id:ebi-a-GCST90000618	TRUE	reported	WO8j2s	textfile	2	TRUE
TRUE	igd	TRUE	rs28435470	rs61952071	G	A	A	C	0.00214831	3.29E-08	Serum 25-Hydroxyvitamin D levels || id:ebi-a-GCST90000618	TRUE	reported	WO8j2s	textfile	2	TRUE
TRUE	igd	NA	NA	NA	NA	NA	NA	NA	0.00308665	2.77E-13	Serum 25-Hydroxyvitamin D levels || id:ebi-a-GCST90000618	TRUE	reported	WO8j2s	textfile	2	TRUE
TRUE	igd	NA	NA	NA	NA	NA	NA	NA	0.00216338	1.41E-09	Serum 25-Hydroxyvitamin D levels || id:ebi-a-GCST90000618	TRUE	reported	WO8j2s	textfile	2	TRUE
TRUE	igd	NA	NA	NA	NA	NA	NA	NA	0.00298008	1.00E-13	Serum 25-Hydroxyvitamin D levels || id:ebi-a-GCST90000618	TRUE	reported	WO8j2s	textfile	2	TRUE
TRUE	igd	NA	NA	NA	NA	NA	NA	NA	0.00227676	2.03E-09	Serum 25-Hydroxyvitamin D levels || id:ebi-a-GCST90000618	TRUE	reported	WO8j2s	textfile	2	TRUE
TRUE	igd	NA	NA	NA	NA	NA	NA	NA	0.00231839	1.33E-11	Serum 25-Hydroxyvitamin D levels || id:ebi-a-GCST90000618	TRUE	reported	WO8j2s	textfile	2	TRUE
TRUE	igd	NA	NA	NA	NA	NA	NA	NA	0.00234858	2.42E-09	Serum 25-Hydroxyvitamin D levels || id:ebi-a-GCST90000618	TRUE	reported	WO8j2s	textfile	2	TRUE
TRUE	igd	NA	NA	NA	NA	NA	NA	NA	0.0026815	5.08E-09	Serum 25-Hydroxyvitamin D levels || id:ebi-a-GCST90000618	TRUE	reported	WO8j2s	textfile	2	TRUE
TRUE	igd	NA	NA	NA	NA	NA	NA	NA	0.00214072	1.65E-27	Serum 25-Hydroxyvitamin D levels || id:ebi-a-GCST90000618	TRUE	reported	WO8j2s	textfile	2	TRUE
TRUE	igd	NA	NA	NA	NA	NA	NA	NA	0.00363266	1.31E-39	Serum 25-Hydroxyvitamin D levels || id:ebi-a-GCST90000618	TRUE	reported	WO8j2s	textfile	2	TRUE
TRUE	igd	NA	NA	NA	NA	NA	NA	NA	0.00298056	1.00E-200	Serum 25-Hydroxyvitamin D levels || id:ebi-a-GCST90000618	TRUE	reported	WO8j2s	textfile	2	TRUE
TRUE	igd	NA	NA	NA	NA	NA	NA	NA	0.00249007	2.76E-09	Serum 25-Hydroxyvitamin D levels || id:ebi-a-GCST90000618	TRUE	reported	WO8j2s	textfile	2	TRUE
TRUE	igd	NA	NA	NA	NA	NA	NA	NA	0.00216263	6.62E-33	Serum 25-Hydroxyvitamin D levels || id:ebi-a-GCST90000618	TRUE	reported	WO8j2s	textfile	2	TRUE
TRUE	igd	NA	NA	NA	NA	NA	NA	NA	0.00256676	1.86E-11	Serum 25-Hydroxyvitamin D levels || id:ebi-a-GCST90000618	TRUE	reported	WO8j2s	textfile	2	TRUE
TRUE	igd	NA	NA	NA	NA	NA	NA	NA	0.00265905	3.95E-13	Serum 25-Hydroxyvitamin D levels || id:ebi-a-GCST90000618	TRUE	reported	WO8j2s	textfile	2	TRUE
TRUE	igd	NA	NA	NA	NA	NA	NA	NA	0.00225079	1.68E-09	Serum 25-Hydroxyvitamin D levels || id:ebi-a-GCST90000618	TRUE	reported	WO8j2s	textfile	2	TRUE
TRUE	igd	NA	NA	NA	NA	NA	NA	NA	0.00204286	2.23E-09	Serum 25-Hydroxyvitamin D levels || id:ebi-a-GCST90000618	TRUE	reported	WO8j2s	textfile	2	TRUE
TRUE	igd	TRUE	rs57601828	rs7977757	T	A	A	G	0.00208192	2.96E-08	Serum 25-Hydroxyvitamin D levels || id:ebi-a-GCST90000618	TRUE	reported	WO8j2s	textfile	2	TRUE
TRUE	igd	NA	NA	NA	NA	NA	NA	NA	0.00221143	1.74E-09	Serum 25-Hydroxyvitamin D levels || id:ebi-a-GCST90000618	TRUE	reported	WO8j2s	textfile	2	TRUE
TRUE	igd	NA	NA	NA	NA	NA	NA	NA	0.00210628	2.44E-11	Serum 25-Hydroxyvitamin D levels || id:ebi-a-GCST90000618	TRUE	reported	WO8j2s	textfile	2	TRUE
TRUE	igd	NA	NA	NA	NA	NA	NA	NA	0.0020505	2.25E-08	Serum 25-Hydroxyvitamin D levels || id:ebi-a-GCST90000618	TRUE	reported	WO8j2s	textfile	2	TRUE
TRUE	igd	NA	NA	NA	NA	NA	NA	NA	0.00658876	1.16E-35	Serum 25-Hydroxyvitamin D levels || id:ebi-a-GCST90000618	TRUE	reported	WO8j2s	textfile	2	TRUE
TRUE	igd	NA	NA	NA	NA	NA	NA	NA	0.00597754	8.05E-10	Serum 25-Hydroxyvitamin D levels || id:ebi-a-GCST90000618	TRUE	reported	WO8j2s	textfile	2	TRUE
TRUE	igd	NA	NA	NA	NA	NA	NA	NA	0.00224413	3.12E-08	Serum 25-Hydroxyvitamin D levels || id:ebi-a-GCST90000618	TRUE	reported	WO8j2s	textfile	2	TRUE
TRUE	igd	NA	NA	NA	NA	NA	NA	NA	0.00276373	1.60E-108	Serum 25-Hydroxyvitamin D levels || id:ebi-a-GCST90000618	TRUE	reported	WO8j2s	textfile	2	TRUE
TRUE	igd	NA	NA	NA	NA	NA	NA	NA	0.0026042	7.55E-09	Serum 25-Hydroxyvitamin D levels || id:ebi-a-GCST90000618	TRUE	reported	WO8j2s	textfile	2	TRUE
TRUE	igd	NA	NA	NA	NA	NA	NA	NA	0.00234031	1.27E-10	Serum 25-Hydroxyvitamin D levels || id:ebi-a-GCST90000618	TRUE	reported	WO8j2s	textfile	2	TRUE
TRUE	igd	NA	NA	NA	NA	NA	NA	NA	0.0025554	2.04E-10	Serum 25-Hydroxyvitamin D levels || id:ebi-a-GCST90000618	TRUE	reported	WO8j2s	textfile	2	TRUE
TRUE	igd	NA	NA	NA	NA	NA	NA	NA	0.00205911	2.26E-12	Serum 25-Hydroxyvitamin D levels || id:ebi-a-GCST90000618	TRUE	reported	WO8j2s	textfile	2	TRUE
TRUE	igd	NA	NA	NA	NA	NA	NA	NA	0.00286182	2.39E-19	Serum 25-Hydroxyvitamin D levels || id:ebi-a-GCST90000618	TRUE	reported	WO8j2s	textfile	2	TRUE
TRUE	igd	NA	NA	NA	NA	NA	NA	NA	0.00209882	9.27E-09	Serum 25-Hydroxyvitamin D levels || id:ebi-a-GCST90000618	TRUE	reported	WO8j2s	textfile	2	TRUE
TRUE	igd	NA	NA	NA	NA	NA	NA	NA	0.00340001	2.93E-08	Serum 25-Hydroxyvitamin D levels || id:ebi-a-GCST90000618	TRUE	reported	WO8j2s	textfile	2	TRUE
TRUE	igd	NA	NA	NA	NA	NA	NA	NA	0.00320607	1.04E-08	Serum 25-Hydroxyvitamin D levels || id:ebi-a-GCST90000618	TRUE	reported	WO8j2s	textfile	2	TRUE
TRUE	igd	NA	NA	NA	NA	NA	NA	NA	0.00243165	8.17E-19	Serum 25-Hydroxyvitamin D levels || id:ebi-a-GCST90000618	TRUE	reported	WO8j2s	textfile	2	TRUE
TRUE	igd	NA	NA	NA	NA	NA	NA	NA	0.00225626	1.49E-09	Serum 25-Hydroxyvitamin D levels || id:ebi-a-GCST90000618	TRUE	reported	WO8j2s	textfile	2	TRUE
TRUE	igd	NA	NA	NA	NA	NA	NA	NA	0.0026652	5.15E-10	Serum 25-Hydroxyvitamin D levels || id:ebi-a-GCST90000618	TRUE	reported	WO8j2s	textfile	2	TRUE
TRUE	igd	TRUE	rs7652808	rs1375545	T	G	G	T	0.00212614	1.36E-23	Serum 25-Hydroxyvitamin D levels || id:ebi-a-GCST90000618	TRUE	reported	WO8j2s	textfile	2	TRUE
TRUE	igd	NA	NA	NA	NA	NA	NA	NA	0.00206215	7.05E-09	Serum 25-Hydroxyvitamin D levels || id:ebi-a-GCST90000618	TRUE	reported	WO8j2s	textfile	2	TRUE
TRUE	igd	TRUE	rs7784802	rs10244141	T	A	T	C	0.0021157	3.06E-10	Serum 25-Hydroxyvitamin D levels || id:ebi-a-GCST90000618	TRUE	reported	WO8j2s	textfile	2	TRUE
TRUE	igd	NA	NA	NA	NA	NA	NA	NA	0.00259019	3.94E-09	Serum 25-Hydroxyvitamin D levels || id:ebi-a-GCST90000618	TRUE	reported	WO8j2s	textfile	2	TRUE
TRUE	igd	NA	NA	NA	NA	NA	NA	NA	0.00905963	6.53E-09	Serum 25-Hydroxyvitamin D levels || id:ebi-a-GCST90000618	TRUE	reported	WO8j2s	textfile	2	TRUE
TRUE	igd	NA	NA	NA	NA	NA	NA	NA	0.00332162	8.33E-09	Serum 25-Hydroxyvitamin D levels || id:ebi-a-GCST90000618	TRUE	reported	WO8j2s	textfile	2	TRUE
TRUE	igd	NA	NA	NA	NA	NA	NA	NA	0.00203856	1.48E-10	Serum 25-Hydroxyvitamin D levels || id:ebi-a-GCST90000618	TRUE	reported	WO8j2s	textfile	2	FALSE
TRUE	igd	NA	NA	NA	NA	NA	NA	NA	0.00266074	1.94E-38	Serum 25-Hydroxyvitamin D levels || id:ebi-a-GCST90000618	TRUE	reported	WO8j2s	textfile	2	TRUE
TRUE	igd	NA	NA	NA	NA	NA	NA	NA	0.00205997	1.20E-14	Serum 25-Hydroxyvitamin D levels || id:ebi-a-GCST90000618	TRUE	reported	WO8j2s	textfile	2	TRUE
TRUE	igd	NA	NA	NA	NA	NA	NA	NA	0.00382316	1.36E-20	Serum 25-Hydroxyvitamin D levels || id:ebi-a-GCST90000618	TRUE	reported	WO8j2s	textfile	2	TRUE
TRUE	igd	NA	NA	NA	NA	NA	NA	NA	0.00254898	1.77E-65	Serum 25-Hydroxyvitamin D levels || id:ebi-a-GCST90000618	TRUE	reported	WO8j2s	textfile	2	TRUE
TRUE	igd	NA	NA	NA	NA	NA	NA	NA	0.00205423	1.21E-08	Serum 25-Hydroxyvitamin D levels || id:ebi-a-GCST90000618	TRUE	reported	WO8j2s	textfile	2	TRUE
TRUE	igd	TRUE	rs9409266	rs700059	G	A	G	A	0.00294656	1.24E-08	Serum 25-Hydroxyvitamin D levels || id:ebi-a-GCST90000618	TRUE	reported	WO8j2s	textfile	2	TRUE
TRUE	igd	NA	NA	NA	NA	NA	NA	NA	0.00298936	3.50E-42	Serum 25-Hydroxyvitamin D levels || id:ebi-a-GCST90000618	TRUE	reported	WO8j2s	textfile	2	TRUE
TRUE	igd	NA	NA	NA	NA	NA	NA	NA	0.00224484	4.19E-08	Serum 25-Hydroxyvitamin D levels || id:ebi-a-GCST90000618	TRUE	reported	WO8j2s	textfile	2	TRUE
TRUE	igd	NA	NA	NA	NA	NA	NA	NA	0.00217811	3.51E-09	Serum 25-Hydroxyvitamin D levels || id:ebi-a-GCST90000618	TRUE	reported	WO8j2s	textfile	2	TRUE
TRUE	igd	NA	NA	NA	NA	NA	NA	NA	0.00407655	9.47E-09	Serum 25-Hydroxyvitamin D levels || id:ebi-a-GCST90000618	TRUE	reported	WO8j2s	textfile	2	TRUE
TRUE	igd	NA	NA	NA	NA	NA	NA	NA	0.021	8.08E-15	C3epi25OHD3	TRUE	reported	x0bgYq	textfile	2	TRUE
TRUE	igd	NA	NA	NA	NA	NA	NA	NA	0.017	1.95E-15	C3epi25OHD3	TRUE	reported	x0bgYq	textfile	2	TRUE
TRUE	igd	NA	NA	NA	NA	NA	NA	NA	0.023	3.22E-60	25OHD3	TRUE	reported	20duX4	textfile	2	TRUE
TRUE	igd	NA	NA	NA	NA	NA	NA	NA	0.01	1.94E-08	25OHD3	TRUE	reported	20duX4	textfile	2	TRUE
TRUE	igd	NA	NA	NA	NA	NA	NA	NA	0.009	1.09E-15	25OHD3	TRUE	reported	20duX4	textfile	2	TRUE
TRUE	igd	NA	NA	NA	NA	NA	NA	NA	0.008	4.08E-59	25OHD3	TRUE	reported	20duX4	textfile	2	TRUE
TRUE	igd	NA	NA	NA	NA	NA	NA	NA	0.007	3.59E-16	25OHD3	TRUE	reported	20duX4	textfile	2	TRUE
TRUE	igd	NA	NA	NA	NA	NA	NA	NA	0.01	1.27E-08	25OHD3	TRUE	reported	20duX4	textfile	2	TRUE
