# Supplementary material for: The causal associations of 25(OH)D and its metabolites with oropharyngeal cancer risk: a Mendelian randomization study
Source: Acta Odontol Scand. 2025 Jul 17;84:44053. doi: 10.2340/aos.v84.44053 (PMC12362939; doi:10.2340/aos.v84.44053)
Supplement: Supplementary file 2 [file AOS-84-44053-s2.pdf]

Supplementary material has been published as submitted. It has not been copyedited or typeset by Acta Odontologica Scandinavica.

Supplementary+Data+2

SNP	CHR	POS	EA	OA	EAF	BETA	SE	P	EXP	N	R2	F
rs11207969	1	62911751	G	A	0.351365	0.0209396	0.00212672	7.14E-23	Serum 25-Hydroxyvitamin D levels || id:ebi-a-GCST90000618	496946	0.000195039	96.94253769
rs11264361	1	155289545	G	T	0.251408	0.0174875	0.00234081	7.97E-14	Serum 25-Hydroxyvitamin D levels || id:ebi-a-GCST90000618	496946	0.000112296	55.81120238
rs61747728	1	179526214	T	C	0.0385611	0.0303061	0.00526894	8.83E-09	Serum 25-Hydroxyvitamin D levels || id:ebi-a-GCST90000618	496946	6.65695E-05	33.08353057
rs2807834	1	220970593	G	T	0.68514	-0.0150625	0.00218678	5.66E-12	Serum 25-Hydroxyvitamin D levels || id:ebi-a-GCST90000618	496946	9.54626E-05	47.44409704
rs512083	1	46027355	C	T	0.462488	0.0122172	0.00204286	2.23E-09	Serum 25-Hydroxyvitamin D levels || id:ebi-a-GCST90000618	496946	7.19657E-05	35.76550894
rs6672758	1	230303512	T	C	0.80023	0.0162478	0.0025554	2.04E-10	Serum 25-Hydroxyvitamin D levels || id:ebi-a-GCST90000618	496946	8.13442E-05	40.42682182
rs2494429	1	2339395	G	A	0.822999	-0.0148459	0.00267333	2.80E-08	Serum 25-Hydroxyvitamin D levels || id:ebi-a-GCST90000618	496946	6.20543E-05	30.83941774
rs1343776	1	41757718	A	G	0.22133	0.0180762	0.00245028	1.62E-13	Serum 25-Hydroxyvitamin D levels || id:ebi-a-GCST90000618	496946	0.000109503	54.42282699
rs7528419	1	109817192	G	A	0.22441	0.0215389	0.00243165	8.17E-19	Serum 25-Hydroxyvitamin D levels || id:ebi-a-GCST90000618	496946	0.000157858	78.45907115
rs115288876	1	152000117	A	G	0.0433211	0.0788065	0.00498229	2.36E-56	Serum 25-Hydroxyvitamin D levels || id:ebi-a-GCST90000618	496946	0.000503197	250.1867621
rs35823191	1	17560123	C	T	0.342049	-0.0232636	0.00214072	1.65E-27	Serum 25-Hydroxyvitamin D levels || id:ebi-a-GCST90000618	496946	0.000237586	118.0952318
rs61813875	1	152536650	G	C	0.0248209	0.0821291	0.00658876	1.16E-35	Serum 25-Hydroxyvitamin D levels || id:ebi-a-GCST90000618	496946	0.000312566	155.3763814
rs1042034	2	21225281	T	C	0.792138	-0.0151254	0.00250017	1.45E-09	Serum 25-Hydroxyvitamin D levels || id:ebi-a-GCST90000618	496946	7.36433E-05	36.59931103
rs1260326	2	27730940	C	T	0.603925	0.0197194	0.00207421	1.96E-21	Serum 25-Hydroxyvitamin D levels || id:ebi-a-GCST90000618	496946	0.000181842	90.38163592
rs35270497	2	38259872	T	C	0.176236	0.0156723	0.0026815	5.08E-09	Serum 25-Hydroxyvitamin D levels || id:ebi-a-GCST90000618	496946	6.87339E-05	34.15923391
rs7569755	2	118648261	A	G	0.288618	0.0136395	0.00225626	1.49E-09	Serum 25-Hydroxyvitamin D levels || id:ebi-a-GCST90000618	496946	7.35322E-05	36.5440658
rs2710651	2	63166379	A	G	0.526101	-0.0115892	0.00203474	1.23E-08	Serum 25-Hydroxyvitamin D levels || id:ebi-a-GCST90000618	496946	6.52757E-05	32.44048378
rs3732220	2	234627048	A	G	0.0852646	-0.0478406	0.00363266	1.31E-39	Serum 25-Hydroxyvitamin D levels || id:ebi-a-GCST90000618	496946	0.000348886	173.4370924
rs727857	2	58981967	A	G	0.61181	-0.0120548	0.00209882	9.27E-09	Serum 25-Hydroxyvitamin D levels || id:ebi-a-GCST90000618	496946	6.63791E-05	32.98890369
rs7580771	2	101428119	T	G	0.175983	-0.0165625	0.0026652	5.15E-10	Serum 25-Hydroxyvitamin D levels || id:ebi-a-GCST90000618	496946	7.7705E-05	38.61805756
rs1047891	2	211540507	A	C	0.317078	-0.0133984	0.00218016	7.96E-10	Serum 25-Hydroxyvitamin D levels || id:ebi-a-GCST90000618	496946	7.59953E-05	37.76829643
rs7652808	3	85603643	G	T	0.649492	-0.0212858	0.00212614	1.36E-23	Serum 25-Hydroxyvitamin D levels || id:ebi-a-GCST90000618	496946	0.000201651	100.2292522
rs9847248	3	18804655	A	G	0.712969	-0.0123077	0.00224484	4.19E-08	Serum 25-Hydroxyvitamin D levels || id:ebi-a-GCST90000618	496946	6.0485E-05	30.05946669
rs13076508	3	52407805	C	T	0.0535034	0.0250544	0.00451036	2.78E-08	Serum 25-Hydroxyvitamin D levels || id:ebi-a-GCST90000618	496946	6.20883E-05	30.85630024
rs1128535	3	49866392	T	C	0.500049	0.0164131	0.0020293	6.06E-16	Serum 25-Hydroxyvitamin D levels || id:ebi-a-GCST90000618	496946	0.00013162	65.41645003
rs6438900	3	125148287	G	C	0.256034	0.0150486	0.00234031	1.27E-10	Serum 25-Hydroxyvitamin D levels || id:ebi-a-GCST90000618	496946	8.31955E-05	41.34696844
rs34186890	3	141720712	G	A	0.259637	-0.0156853	0.00231839	1.33E-11	Serum 25-Hydroxyvitamin D levels || id:ebi-a-GCST90000618	496946	9.21008E-05	45.77316274
rs1949633	3	153758806	C	T	0.605759	0.0114162	0.00208637	4.45E-08	Serum 25-Hydroxyvitamin D levels || id:ebi-a-GCST90000618	496946	6.02456E-05	29.94047807
rs6834488	4	88178919	T	C	0.422833	-0.01445	0.00205911	2.26E-12	Serum 25-Hydroxyvitamin D levels || id:ebi-a-GCST90000618	496946	9.90888E-05	49.24644111
rs13108245	4	57790205	G	A	0.386601	-0.0122246	0.00208616	4.63E-09	Serum 25-Hydroxyvitamin D levels || id:ebi-a-GCST90000618	496946	6.90931E-05	34.33778991
rs3114045	4	100252560	C	T	0.866137	-0.0221737	0.00298008	1.00E-13	Serum 25-Hydroxyvitamin D levels || id:ebi-a-GCST90000618	496946	0.000111394	55.36288856
rs78649910	4	3482213	A	T	0.105728	-0.0191377	0.00332162	8.33E-09	Serum 25-Hydroxyvitamin D levels || id:ebi-a-GCST90000618	496946	6.67946E-05	33.19539519
rs4348160	4	70017531	G	T	0.326968	-0.0258401	0.00216263	6.62E-33	Serum 25-Hydroxyvitamin D levels || id:ebi-a-GCST90000618	496946	0.000287204	142.7651433
rs4147536	4	100239112	C	A	0.788613	-0.0148038	0.00249007	2.76E-09	Serum 25-Hydroxyvitamin D levels || id:ebi-a-GCST90000618	496946	7.11186E-05	35.34447689
rs4364259	4	15892159	A	G	0.198665	0.0172408	0.00256676	1.86E-11	Serum 25-Hydroxyvitamin D levels || id:ebi-a-GCST90000618	496946	9.07811E-05	45.11724315
rs71599974	4	71765339	G	A	0.148048	0.0257378	0.00286182	2.39E-19	Serum 25-Hydroxyvitamin D levels || id:ebi-a-GCST90000618	496946	0.000162734	80.88285393
rs12501515	4	72592838	A	G	0.58966	-0.078957	0.00206932	1.00E-200	Serum 25-Hydroxyvitamin D levels || id:ebi-a-GCST90000618	496946	0.002921099	1455.875438
rs11726886	4	72822599	A	C	0.290783	-0.0536726	0.00225483	3.08E-125	Serum 25-Hydroxyvitamin D levels || id:ebi-a-GCST90000618	496946	0.001138868	566.5991401
rs7712001	5	148020950	G	T	0.440176	0.011939	0.00206215	7.05E-09	Serum 25-Hydroxyvitamin D levels || id:ebi-a-GCST90000618	496946	6.74461E-05	33.51920052
rs986649	5	118668050	G	A	0.321628	0.0128637	0.00217811	3.51E-09	Serum 25-Hydroxyvitamin D levels || id:ebi-a-GCST90000618	496946	7.01831E-05	34.87951525
rs17207784	6	22768668	C	T	0.324195	-0.0134939	0.0021713	5.14E-10	Serum 25-Hydroxyvitamin D levels || id:ebi-a-GCST90000618	496946	7.77128E-05	38.62189352
rs1321247	6	25662873	T	A	0.101578	-0.0221847	0.00336578	4.36E-11	Serum 25-Hydroxyvitamin D levels || id:ebi-a-GCST90000618	496946	8.74155E-05	43.4444118
rs12153819	6	83773049	T	C	0.123154	-0.0178178	0.0030906	8.16E-09	Serum 25-Hydroxyvitamin D levels || id:ebi-a-GCST90000618	496946	6.68782E-05	33.23692302
rs742493	6	40998167	C	T	0.112871	0.0183528	0.00320607	1.04E-08	Serum 25-Hydroxyvitamin D levels || id:ebi-a-GCST90000618	496946	6.59357E-05	32.76852668
rs2245133	6	131931092	C	T	0.164339	-0.0212917	0.00273999	7.80E-15	Serum 25-Hydroxyvitamin D levels || id:ebi-a-GCST90000618	496946	0.000121496	60.38387413
rs9375037	6	121856794	C	A	0.443168	0.0117059	0.00205423	1.21E-08	Serum 25-Hydroxyvitamin D levels || id:ebi-a-GCST90000618	496946	6.53392E-05	32.47205223
rs1858889	7	107117447	C	A	0.502532	0.0134514	0.00203065	3.49E-11	Serum 25-Hydroxyvitamin D levels || id:ebi-a-GCST90000618	496946	8.82912E-05	43.87964203
rs2595644	7	43980540	T	G	0.384889	-0.0122625	0.00209685	4.97E-09	Serum 25-Hydroxyvitamin D levels || id:ebi-a-GCST90000618	496946	6.88152E-05	34.1996424
rs7784802	7	64015379	T	A	0.358913	0.0133202	0.0021157	3.06E-10	Serum 25-Hydroxyvitamin D levels || id:ebi-a-GCST90000618	496946	7.97571E-05	39.63798523
rs10277163	7	21569089	G	A	0.254715	-0.0143336	0.0023507	1.08E-09	Serum 25-Hydroxyvitamin D levels || id:ebi-a-GCST90000618	496946	7.48125E-05	37.1804292
rs804281	8	11611865	G	A	0.583498	0.015895	0.00205997	1.20E-14	Serum 25-Hydroxyvitamin D levels || id:ebi-a-GCST90000618	496946	0.000119795	59.53845016
rs1384687	8	61525963	A	G	0.132271	-0.0168656	0.00299675	1.82E-08	Serum 25-Hydroxyvitamin D levels || id:ebi-a-GCST90000618	496946	6.37332E-05	31.67384712
rs12056768	8	116988527	G	T	0.584044	-0.0231961	0.00206406	2.65E-29	Serum 25-Hydroxyvitamin D levels || id:ebi-a-GCST90000618	496946	0.000254077	126.2942452
rs34726834	8	25889606	T	C	0.252209	0.014013	0.00234858	2.42E-09	Serum 25-Hydroxyvitamin D levels || id:ebi-a-GCST90000618	496946	7.16327E-05	35.59998211
rs9409266	9	125745042	A	G	0.862222	-0.0167771	0.00294656	1.24E-08	Serum 25-Hydroxyvitamin D levels || id:ebi-a-GCST90000618	496946	6.52327E-05	32.41913781
rs13294734	9	80710910	T	C	0.466128	0.012568	0.00205837	1.02E-09	Serum 25-Hydroxyvitamin D levels || id:ebi-a-GCST90000618	496946	7.50142E-05	37.28067002
rs635634	9	136155000	T	C	0.186573	-0.0150476	0.0026042	7.55E-09	Serum 25-Hydroxyvitamin D levels || id:ebi-a-GCST90000618	496946	6.71811E-05	33.38751217
rs11791258	9	107632644	A	G	0.191072	0.0140808	0.00258052	4.85E-08	Serum 25-Hydroxyvitamin D levels || id:ebi-a-GCST90000618	496946	5.99108E-05	29.77408702
rs2398113	10	10076429	G	A	0.423526	-0.0117606	0.00205797	1.10E-08	Serum 25-Hydroxyvitamin D levels || id:ebi-a-GCST90000618	496946	6.57118E-05	32.65721392
rs12775091	10	91524012	T	C	0.213441	0.0155618	0.00247693	3.33E-10	Serum 25-Hydroxyvitamin D levels || id:ebi-a-GCST90000618	496946	7.94234E-05	39.47211928
rs2297991	10	113913222	C	T	0.718478	0.0127547	0.00225578	1.57E-08	Serum 25-Hydroxyvitamin D levels || id:ebi-a-GCST90000618	496946	6.43295E-05	31.97019349
rs77532868	10	88081438	T	C	0.0521528	0.0259557	0.00456301	1.28E-08	Serum 25-Hydroxyvitamin D levels || id:ebi-a-GCST90000618	496946	6.51066E-05	32.35645268
rs144965707	11	14059511	A	G	0.0618296	-0.0348143	0.00421754	1.52E-16	Serum 25-Hydroxyvitamin D levels || id:ebi-a-GCST90000618	496946	0.000137097	68.13890756
rs1627043	11	71110175	C	G	0.0332414	-0.0486441	0.00566106	8.49E-18	Serum 25-Hydroxyvitamin D levels || id:ebi-a-GCST90000618	496946	0.000148556	73.83513641
rs2847500	11	120114421	A	G	0.123204	-0.022548	0.00308665	2.77E-13	Serum 25-Hydroxyvitamin D levels || id:ebi-a-GCST90000618	496946	0.000107371	53.36291318
rs17473257	11	14283186	A	G	0.0172466	-0.0611372	0.00780044	4.59E-15	Serum 25-Hydroxyvitamin D levels || id:ebi-a-GCST90000618	496946	0.000123598	61.42867394
rs117300835	11	15118975	A	G	0.0133061	-0.334985	0.00886005	1.00E-200	Serum 25-Hydroxyvitamin D levels || id:ebi-a-GCST90000618	496946	0.002868281	1429.475209

**rs2511279**	11	71130419	G	C	0.960373	0.0981721	0.00520826	2.98E-79	Serum 25-Hydroxyvitamin D levels || id:ebi-a-GCST90000618	496946	0.000714449	355.2950047
**rs3829251**	11	71194559	A	G	0.133277	-0.114453	0.00298056	1.00E-200	Serum 25-Hydroxyvitamin D levels || id:ebi-a-GCST90000618	496946	0.002958439	1474.541079
**rs11023159**	11	14262063	C	T	0.0325143	0.0482117	0.00572507	3.73E-17	Serum 25-Hydroxyvitamin D levels || id:ebi-a-GCST90000618	496946	0.000142683	70.91556067
**rs733454**	11	76477721	T	C	0.0991663	0.0188545	0.00340001	2.93E-08	Serum 25-Hydroxyvitamin D levels || id:ebi-a-GCST90000618	496946	6.18776E-05	30.75161319
**rs111515741**	11	14370944	A	G	0.0173081	-0.0487364	0.00779044	3.95E-10	Serum 25-Hydroxyvitamin D levels || id:ebi-a-GCST90000618	496946	7.8748E-05	39.13642668
**rs12283049**	11	14690192	G	A	0.234433	-0.0564566	0.00240614	9.62E-122	Serum 25-Hydroxyvitamin D levels || id:ebi-a-GCST90000618	496946	0.001106618	550.5362321
**rs11600054**	11	14690511	A	G	0.0100965	0.0681747	0.0101478	1.84E-11	Serum 25-Hydroxyvitamin D levels || id:ebi-a-GCST90000618	496946	9.08143E-05	45.13369863
**rs964184**	11	116648917	C	G	0.867234	0.0406845	0.00298936	3.50E-42	Serum 25-Hydroxyvitamin D levels || id:ebi-a-GCST90000618	496946	0.000372589	185.2250754
**rs61887421**	11	70949673	C	T	0.0300871	-0.036726	0.00597754	8.05E-10	Serum 25-Hydroxyvitamin D levels || id:ebi-a-GCST90000618	496946	7.59557E-05	37.74857224
**rs7955128**	12	38684121	T	A	0.52053	0.0130617	0.00203856	1.48E-10	Serum 25-Hydroxyvitamin D levels || id:ebi-a-GCST90000618	496946	8.26052E-05	41.05354501
**rs1038165**	12	68665940	T	C	0.579451	0.0115149	0.00205629	2.15E-08	Serum 25-Hydroxyvitamin D levels || id:ebi-a-GCST90000618	496946	6.30979E-05	31.35810909
**rs73413596**	12	111582630	C	T	0.0740317	0.0223468	0.00388928	9.15E-09	Serum 25-Hydroxyvitamin D levels || id:ebi-a-GCST90000618	496946	6.64285E-05	33.0134199
**rs28435470**	12	133067473	A	G	0.663204	-0.0118696	0.00214831	3.29E-08	Serum 25-Hydroxyvitamin D levels || id:ebi-a-GCST90000618	496946	6.14246E-05	30.52646529
**rs57601828**	12	93192127	T	A	0.394508	0.011542	0.00208192	2.96E-08	Serum 25-Hydroxyvitamin D levels || id:ebi-a-GCST90000618	496946	6.18441E-05	30.73493631
**rs1871395**	12	21352315	G	A	0.152679	-0.0203733	0.00282689	5.72E-13	Serum 25-Hydroxyvitamin D levels || id:ebi-a-GCST90000618	496946	0.000104508	51.9401493
**rs2171427**	12	24822154	A	G	0.156491	-0.0165489	0.00281738	4.26E-09	Serum 25-Hydroxyvitamin D levels || id:ebi-a-GCST90000618	496946	6.94237E-05	34.50211043
**rs10859995**	12	96375682	C	T	0.579841	-0.0436264	0.00205452	4.60E-100	Serum 25-Hydroxyvitamin D levels || id:ebi-a-GCST90000618	496946	0.000906515	450.8958722
**rs4580037**	13	55702646	C	A	0.285578	-0.0135627	0.00225079	1.68E-09	Serum 25-Hydroxyvitamin D levels || id:ebi-a-GCST90000618	496946	7.30603E-05	36.30952848
**rs8018720**	14	39556185	C	G	0.823508	-0.0344962	0.00266074	1.94E-38	Serum 25-Hydroxyvitamin D levels || id:ebi-a-GCST90000618	496946	0.000338128	168.0876824
**rs2756119**	14	104001517	A	G	0.38143	0.0121434	0.0021104	8.71E-09	Serum 25-Hydroxyvitamin D levels || id:ebi-a-GCST90000618	496946	6.66213E-05	33.10924651
**rs142004400**	14	50829560	C	A	0.0342066	-0.0310034	0.00559561	3.01E-08	Serum 25-Hydroxyvitamin D levels || id:ebi-a-GCST90000618	496946	6.17714E-05	30.69884414
**rs1532085**	15	58683366	G	A	0.616672	0.0252805	0.00208639	8.60E-34	Serum 25-Hydroxyvitamin D levels || id:ebi-a-GCST90000618	496946	0.000295354	146.8177572
**rs1800588**	15	58723675	T	C	0.214964	-0.0305021	0.00246932	4.73E-35	Serum 25-Hydroxyvitamin D levels || id:ebi-a-GCST90000618	496946	0.000306946	152.5818883
**rs62007299**	15	77711719	A	G	0.712958	-0.0124205	0.00224413	3.12E-08	Serum 25-Hydroxyvitamin D levels || id:ebi-a-GCST90000618	496946	6.16377E-05	30.63235507
**rs325393**	15	100229260	T	G	0.278218	-0.0136497	0.00227676	2.03E-09	Serum 25-Hydroxyvitamin D levels || id:ebi-a-GCST90000618	496946	7.23221E-05	35.94263886
**rs12324720**	15	64092140	A	G	0.174629	-0.0149159	0.00267451	2.45E-08	Serum 25-Hydroxyvitamin D levels || id:ebi-a-GCST90000618	496946	6.25856E-05	31.10346163
**rs1684600**	16	4594671	T	C	0.298695	-0.0125301	0.00221719	1.59E-08	Serum 25-Hydroxyvitamin D levels || id:ebi-a-GCST90000618	496946	6.42638E-05	31.93754312
**rs11542462**	16	82033810	A	G	0.133511	-0.0247803	0.00298265	9.72E-17	Serum 25-Hydroxyvitamin D levels || id:ebi-a-GCST90000618	496946	0.00013888	69.02505858
**rs77924615**	16	20392332	A	G	0.194374	-0.0152475	0.00259019	3.94E-09	Serum 25-Hydroxyvitamin D levels || id:ebi-a-GCST90000618	496946	6.9726E-05	34.65231802
**rs11076175**	16	57006378	G	A	0.175666	0.0229033	0.00266997	9.64E-18	Serum 25-Hydroxyvitamin D levels || id:ebi-a-GCST90000618	496946	0.00014805	73.58369844
**rs11867297**	17	66433493	T	C	0.38533	0.0135432	0.00209454	1.01E-10	Serum 25-Hydroxyvitamin D levels || id:ebi-a-GCST90000618	496946	8.41239E-05	41.80839724
**rs61698755**	17	79257880	C	T	0.560041	-0.011465	0.0020505	2.25E-08	Serum 25-Hydroxyvitamin D levels || id:ebi-a-GCST90000618	496946	6.2906E-05	31.26272447
**rs9946771**	18	28918628	T	C	0.0663426	-0.0233992	0.00407655	9.47E-09	Serum 25-Hydroxyvitamin D levels || id:ebi-a-GCST90000618	496946	6.62946E-05	32.94691263
**rs2037511**	18	61366207	A	G	0.165924	0.0176624	0.00272732	9.41E-11	Serum 25-Hydroxyvitamin D levels || id:ebi-a-GCST90000618	496946	8.43881E-05	41.93971642
**rs77960347**	18	47109955	G	A	0.0127013	-0.0525688	0.00905963	6.53E-09	Serum 25-Hydroxyvitamin D levels || id:ebi-a-GCST90000618	496946	6.7748E-05	33.66925109
**rs10438978**	18	47158186	C	T	0.820277	-0.0172243	0.00264437	7.34E-11	Serum 25-Hydroxyvitamin D levels || id:ebi-a-GCST90000618	496946	8.53675E-05	42.42647709
**rs1048328**	19	51527364	A	G	0.0797967	0.0313497	0.00374376	5.58E-17	Serum 25-Hydroxyvitamin D levels || id:ebi-a-GCST90000618	496946	0.000141085	70.12115016
**rs142158911**	19	11190534	A	G	0.111826	0.026284	0.00323446	4.43E-16	Serum 25-Hydroxyvitamin D levels || id:ebi-a-GCST90000618	496946	0.000132866	66.03551966
**rs12462826**	19	11955767	A	G	0.369566	-0.0132119	0.0021149	4.18E-10	Serum 25-Hydroxyvitamin D levels || id:ebi-a-GCST90000618	496946	7.85249E-05	39.02555908
**rs4420638**	19	45422946	G	A	0.176831	-0.0192973	0.00265905	3.95E-13	Serum 25-Hydroxyvitamin D levels || id:ebi-a-GCST90000618	496946	0.00010597	52.66697099
**rs8107974**	19	19388500	T	A	0.0763013	0.0355672	0.00382316	1.36E-20	Serum 25-Hydroxyvitamin D levels || id:ebi-a-GCST90000618	496946	0.000174128	86.54712762
**rs62129966**	19	48374950	A	C	0.160854	0.0611636	0.00276373	1.60E-108	Serum 25-Hydroxyvitamin D levels || id:ebi-a-GCST90000618	496946	0.000984595	489.771044
**rs1841850**	20	52718179	C	A	0.117085	0.0304395	0.00316491	6.73E-22	Serum 25-Hydroxyvitamin D levels || id:ebi-a-GCST90000618	496946	0.000186107	92.50187839
**rs8121940**	20	52742306	G	C	0.19762	-0.0435612	0.00254898	1.77E-65	Serum 25-Hydroxyvitamin D levels || id:ebi-a-GCST90000618	496946	0.000587357	292.0552828
**rs6129648**	20	39231118	G	A	0.379837	0.0140631	0.00210628	2.44E-11	Serum 25-Hydroxyvitamin D levels || id:ebi-a-GCST90000618	496946	8.96978E-05	44.57877951
**rs290400**	20	52698179	A	G	0.665472	-0.0130967	0.00216338	1.41E-09	Serum 25-Hydroxyvitamin D levels || id:ebi-a-GCST90000618	496946	7.37423E-05	36.64851572
**rs2229742**	21	16339172	C	G	0.104659	-0.0249837	0.00331409	4.75E-14	Serum 25-Hydroxyvitamin D levels || id:ebi-a-GCST90000618	496946	0.000114347	56.83072131
**rs138335**	22	41227086	G	C	0.658587	-0.0137663	0.00215115	1.56E-10	Serum 25-Hydroxyvitamin D levels || id:ebi-a-GCST90000618	496946	8.2404E-05	40.9535281
**rs2074735**	22	31535872	C	G	0.0648365	0.0292667	0.00412	1.22E-12	Serum 25-Hydroxyvitamin D levels || id:ebi-a-GCST90000618	496946	0.000101531	50.4604747
**rs5770794**	22	50880781	T	C	0.3143	-0.0133141	0.00221143	1.74E-09	Serum 25-Hydroxyvitamin D levels || id:ebi-a-GCST90000618	496946	7.29351E-05	36.24728541
**rs13084927**	3	72709792	C	A	0.83	0.055	0.01	1.94E-08	25OHD3	40562	0.000745216	30.24850846
**rs4588**	4	72618323	G	T	0.71	0.266	0.008	6.55E-266	25OHD3	40562	0.02653293	1105.507988
**rs116970203**	11	14876718	G	A	0.98	0.372	0.023	3.22E-60	25OHD3	40562	0.006407948	261.5825646
**rs28364617**	11	71159764	G	T	0.71	0.127	0.008	4.08E-59	25OHD3	40562	0.006174732	252.0031988
**rs3819817**	12	96378771	C	T	0.45	0.058	0.007	3.59E-16	25OHD3	40562	0.001689686	68.64967613
**rs9304669**	19	48384385	T	C	0.16	0.054	0.01	1.27E-08	25OHD3	40562	0.000718383	29.1585622
**rs17216707**	20	52732362	T	C	0.8	0.074	0.009	1.09E-15	25OHD3	40562	0.001663933	67.60160486
**rs4588**	4	72618323	G	T	0.71	0.194	0.016	2.48E-32	C3epi25OHD3	40562	0.003611378	147.0083761
**rs28364617**	11	71159764	G	T	0.72	0.131	0.017	1.95E-15	C3epi25OHD3	40562	0.001461807	59.37769494
**rs11172066**	12	57319491	T	A	0.14	0.166	0.021	8.08E-15	C3epi25OHD3	40562	0.001538118	62.4821798
